# Common and rare genetic variant associations with cognitive performance across development in British birth cohorts

**DOI:** 10.1038/s41562-026-02491-8

**Published:** 2026-07-10

**Authors:** Daniel S. Malawsky, Mahmoud Koko, Petr Danacek, Wei Huang, Olivia Wootton, Qin Qin Huang, Emma E. Wade, Sarah J. Lindsay, Rosalind Arden, Matthew E. Hurles, Hilary C. Martin

**Affiliations:** 1 Human Genetics Programme, Wellcome Sanger Institute, Hinxton, UK; 2 Bioinformatics Interdepartmental Program, University of California, Los Angeles, USA; 3 Centre for Philosophy of Natural and Social Science, London School of Economics and Political Science, London, UK

**Keywords:** Behavioural genetics, Development, Neurodevelopmental disorders

## Abstract

Genetic variants that correlate with adult cognitive performance are also associated with rare neurodevelopmental conditions involving cognitive deficits in children. However, their association with cognition across early life remains poorly understood. Using 6,495 children from the Avon Longitudinal Study of Parents and Children, we show that, as children age, the association between cognitive ability and polygenic indices for educational attainment and its cognitive component increases (PGI_EA_ × age interaction per 10 years: 0.033 [0.020, 0.047], PGI_Cog_ × age: 0.0232 [0.0096, 0.0367]). Conversely, negative associations with deleterious rare variant burden attenuate with age (RVB_pLoF_ × age: 0.0239 [0.0110, 0.0368]). Using trio analyses, we show that these age-related trends are consistent with contributions from direct genetic effects. We find that the increasing associations with polygenic indices are stronger in individuals at the upper end of the phenotype distribution, whereas the attenuating associations with rare variant burden are stronger in those at the lower end. Our findings may help explain the apparent incomplete penetrance of rare damaging variants associated with neurodevelopmental conditions.

## Abstract (web summary)

Associations between polygenic indices and cognitive ability in children increase with age while rare damaging variant associations attenuate, with differential effects across the cognitive ability distribution.

## Main

Cognitive ability is an important predictor of life outcomes such as health and education^1–6^, and is associated with both by genetic and environmental factors^7^. Results from twin studies show that the heritability of cognitive ability increases throughout development, from ~0.2 in infancy to ~0.6 in adulthood^8^. The heritability of cognitive performance attributable to common single-nucleotide polymorphisms (the ‘SNP heritability’) has been estimated to be 0.20 in adults^9^. SNP heritability is likely to be explained both by direct genetic effects^9,10^ (the associations of genetic variants in an individual on that individual’s own phenotype) and other factors. These other factors are thought to include the indirect effects of ‘genetic nurture’ (the effects of genetically influenced behaviours of family members on the index individual’s phenotype), parental assortment (phenotypic correlation between partners, commonly known as ‘assortative mating’) and uncontrolled population stratification in genome-wide association studies (GWASs)^11–14^. The relative contribution of these factors to genetic associations is still a matter of debate^11,15–18^. However, the role of direct versus indirect genetic effects on cognitive ability across different timepoints in development is understudied, and we explore this in this work.

Genetic factors correlating with cognitive ability in the general population also correlate with risk of rare neurodevelopmental conditions (NDCs) involving cognitive impairment. While Mendelian-acting rare coding variants play a large role in NDCs^19–21^, common variants explain ~10% of variation in risk on the liability scale^22,23^. This common variant risk is negatively genetically correlated with educational attainment (EA) and adult cognitive performance in the general population^22,23^. In addition, rare damaging coding variants in NDC-associated genes are associated with lower fluid intelligence and lower EA in UK Biobank^24–26^. Estimates suggest that rare coding variants exome-wide explain 1% of the variance in adult cognitive performance^27^.

Findings from both clinical^28,29^ and population cohorts^26,30^ suggest that some rare variants conferring risk of NDCs are often shared between affected children and their seemingly unaffected parents. This so-called ‘incomplete penetrance’ is seen for large copy-number variants^29^ as well as predicted loss-of-function variants (pLoFs) and damaging missense variants in ‘constrained genes’^31^ (that is, genes in which such variants have been under negative selection throughout recent human evolution). It is frequently assumed that multiple genetic and environmental factors contribute to an underlying liability for NDCs, and children are diagnosed if they cross some phenotypic threshold^32^. Under this model, incomplete penetrance of rare variants is expected if they increase the mean of the liability distribution in carriers to a point at which some but not all cross the threshold for diagnosis. It could also occur if the variants increase the variance in liability even without a change in mean liability of carriers, by pushing more individuals to the distribution extremes. Another potential contributor to apparent incomplete penetrance could be time-varying effects; that is, rare variants (or at least a subset of them) are associated with cognitive ability in childhood more than they do in adulthood. We investigate this here.

In this work, we sought to dissect the contribution of polygenic indices for educational attainment and its cognitive and non-cognitive components and of rare variant burden to cognitive performance across childhood and adolescence, using genotype data and new exome sequence data (*N* = 16,103 children and 10,140 parents) from two British birth cohorts, the Avon Longitudinal Study of Parents and Children (ALSPAC)^33^ and the Millenium Cohort Study (MCS)^34^. Our first aim was to examine the associations of genetic measures with average cognitive performance and academic achievement as children age, then test whether these change across time and the extent to which they are due to direct genetic effects. Our second aim was to explore whether these genetic measures have differential associations at the tails of the phenotype distribution (in other words, whether they are associated with the variance in cognitive ability). Finally, our third aim was to compare these genetic associations to those of parental educational attainment and perinatal exposures, and to explore whether they are robust to controlling for these other measures. Our results illuminate the dynamic genetic architecture of cognitive performance across development and shed light on the factors that best predict cognitive impairment in childhood as opposed to average cognitive performance in adulthood.

## Results

**Extended Data Fig. 1:**
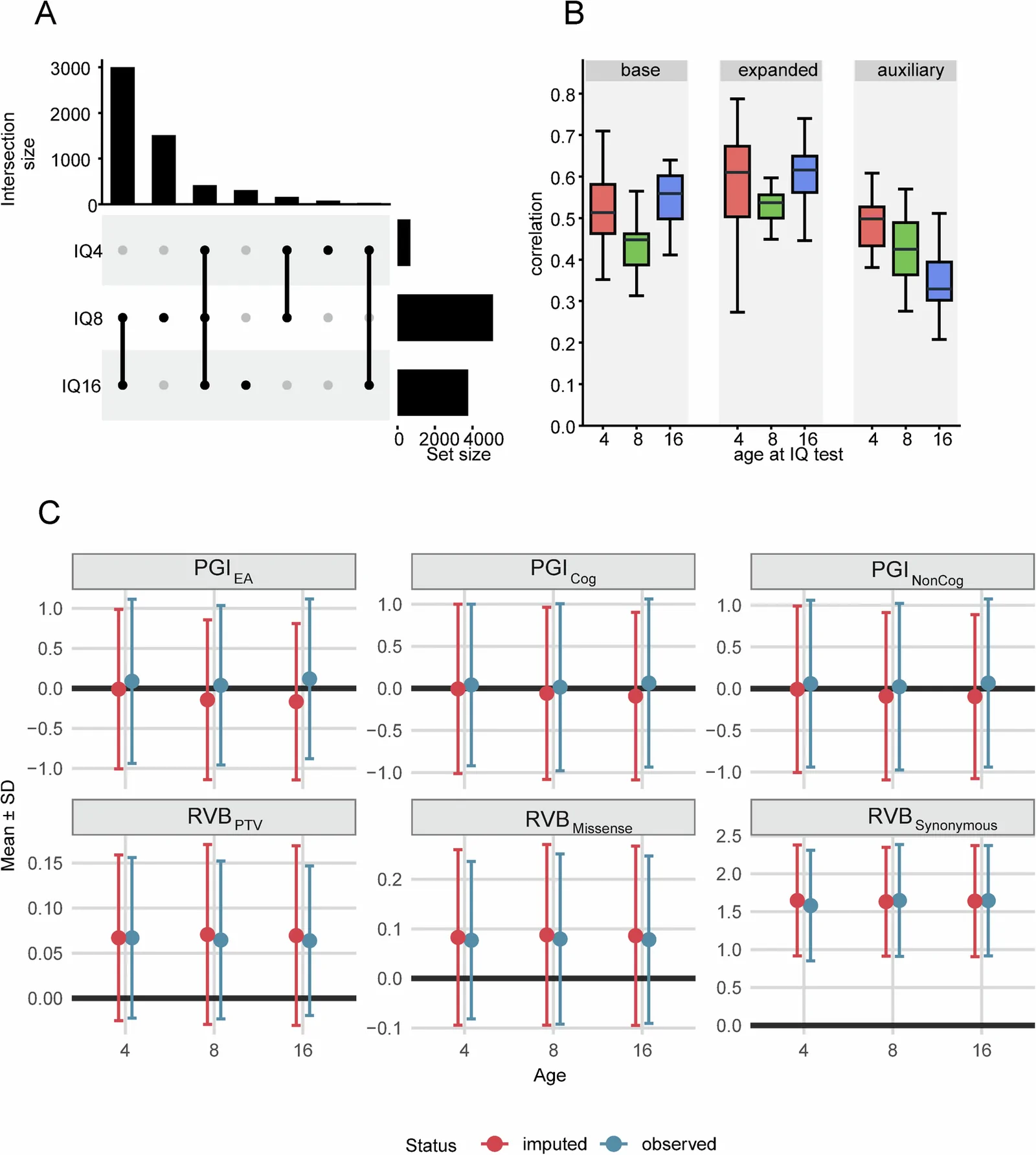
Assessing quality of IQ imputation across ages and sets of variables and genetic profile of individuals with measured IQ in ALSPAC. **A**) Upset plot indicating number of individuals with a given set of measured IQ values. **B**) Correlations between masked measured and imputed values for 100 random individuals for 100 trials for three different combinations of variables (base, expanded and auxiliary variables, as described in Methods) used in the SoftImpute imputation for IQ measured at age 4, 8, and 16. Box plots show the minimum, 1st quartile, median, third quartile, and maximum values. **C**) Mean and standard deviation of genetic scores for individuals where an IQ measurement was either observed or imputed at ages 4 (n observed = 701, imputed = 5794), 8 (5134, 1361), and 16 (3774, 2721) among those with genetic data.

In this work, we used measurements of cognitive performance and/or school achievement from three cohorts: ALSPAC, MCS and the UK Biobank. Our primary analyses were based on measures of IQ from ALSPAC that were collected at ages 4 (*n* = 1,012), 8 (*n* = 7,347) and 16 (*n* = 5,270). These IQ tests have good psychometric properties^35^ and are longitudinally invariant, meaning that they measure the same latent construct across ages^36^, making them suitable for longitudinal analyses. We observed that IQ missingness was non-random with respect to individuals’ polygenic indices (PGIs) for educational attainment, particularly among individuals with measures at age 4 and 16 (Supplementary Note 1 and Extended Data Fig. 1c). This suggests subtle biases in which individuals did the IQ tests at the different ages, addressed further below. Since phenotypic missingness can induce ascertainment biases and reduce power, we imputed missing IQ values across ages among participants who had at least one IQ measure at any timepoint (*n* = 8,207) (Supplementary Note 1 and Extended Data Fig. 1). Briefly, we imputed missing IQ measures using SoftImpute^37^, which leverages correlations with other variables. These included cognitive and behavioural tests, birth weight and demographic variables (see Methods). We carried out analyses on both the observed IQ values (‘pre-imputation’) and on the observed and imputed IQ values combined (‘post-imputation’). Results were largely concordant between these unless specifically noted otherwise.

### Association between common variants and cognitive performance across development

We first considered the heritabilities and genetic correlations of IQ measured at different ages in ALSPAC, using 6,495 unrelated children with SNP genotype data, genetically inferred European ancestry and observed or imputed IQ values. Using GREML-LDMS^38^, we estimated that the heritability of IQ increased between ages 4 to 16 from 0.46 to 0.56 (post-imputation), although the increase was not significant (*β* = 0.0086, *t*_(1)_ = 6.93, *P* = 0.091, 95% confidence interval (CI) [−0.007, 0.024]; inverse-variance weighted regression) (Supplementary Table 2). We found no evidence that the pairwise genetic correlations between IQ measures from different ages were distinguishable from 1 (Supplementary Table 2), suggesting that the common variant genetic architecture is largely stable across development, consistent with previous studies^39,40^.

**Fig. 1:**
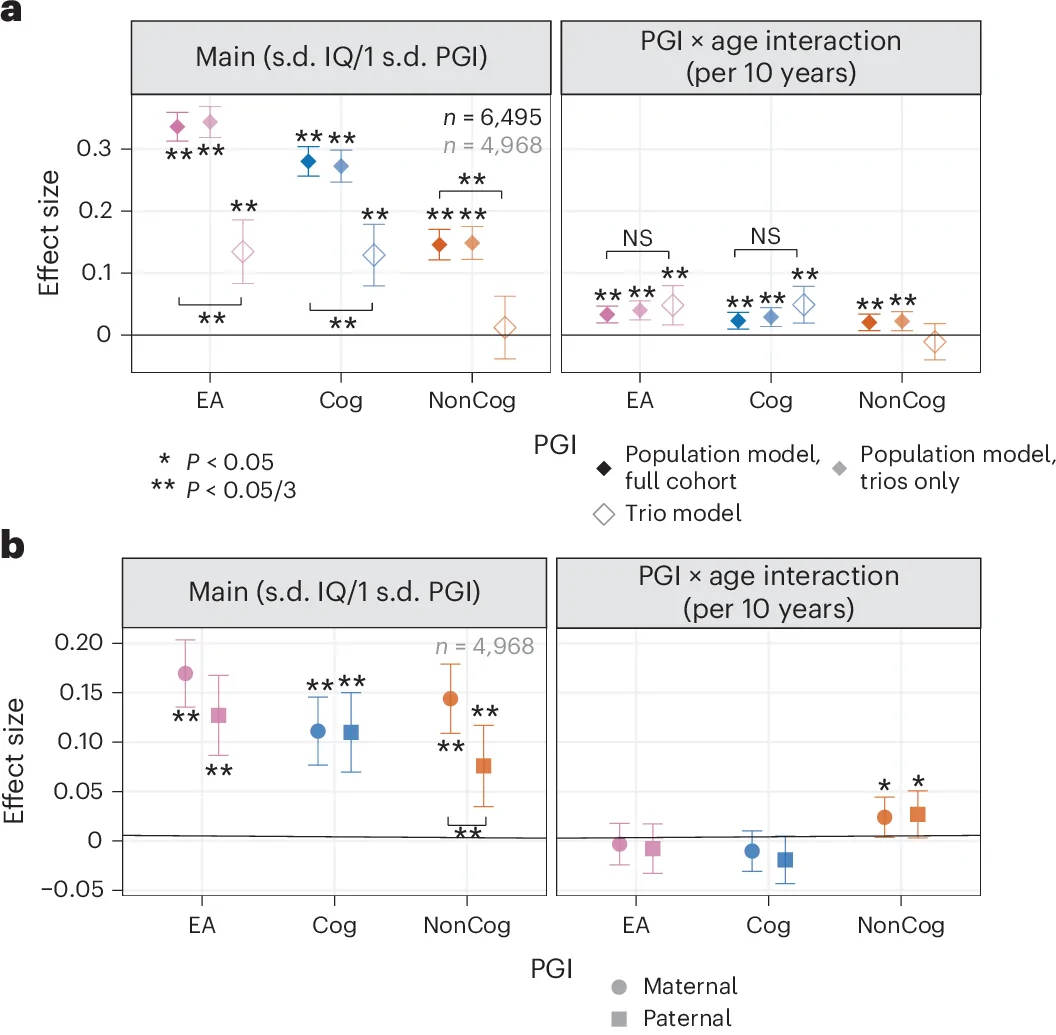
Association between PGIs for educational attainment and its cognitive/non-cognitive components and IQ across ages. Standardized effect sizes and 95% CIs estimated for the main effects (left) and PGI × age interaction effects (right) for each PGI post-imputation of IQ (see Supplementary Fig. 1 for pre-imputation results). **a**, Results for the children’s PGIs not controlling (that is, ‘population model’) and controlling (that is, ‘trio model’) for parental PGIs. Population effect sizes are shown estimated in the full sample (opaque; *n* = 6,495) and in the subset of children with parental PGIs (translucent; *n* = 4,968). **b**, Standardized effect sizes and 95% CIs for the parental PGIs from the trio model (*n* = 4,968 trios). Effect sizes and *P* values were estimated using mixed-effects linear regression (two-sided Wald test). PGI, polygenic index; EA, educational attainment; Cog, cognitive component of EA from ref. ^16^; NonCog, non-cognitive component of EA from ref. ^16^. The square brackets indicate significant comparisons highlighted in the text (two-tailed *Z*-test). The *P* values for the difference in population and direct main effect sizes are *P* = 2.2 × 10^−12^, 7.35 × 10^−8^ and 2.69 × 10^−8^ for PGI_EA_, PGI_Cog_ and PGI_NonCog_, respectively). The *P* values for the difference in population and direct interaction effect sizes are *P* = 0.91 and 0.58 for PGI_EA_ and PGI_Cog_, respectively). The *P* values for the difference in paternal and maternal PGI_NonCog_ effects is 0.014. Point estimates, 95% CIs and *P* values are provided in Supplementary Table 4.

We then considered the associations between IQ and several PGIs, first considering a PGI for EA (PGI_EA_). EA has a cognitive component but also a non-cognitive component which may capture factors such as personality traits and socioeconomic status^41^. Genetic associations with these two components have been previously derived from GWASs in adults using genomic structural equation modelling^41^, so we also included PGIs representing each of them (PGI_Cog_ and PGI_NonCog_). For each PGI, we modelled IQ across all ages as the outcome of individual-specific random effects and several fixed effects, namely, age (relative to age 4), the PGI and their interaction, with sex and genetic principal components as covariates. With this mixed-effects linear model, we estimated two parameters of interest: (1) the association between the phenotype and the PGI at baseline, that is, at age 4 (the ‘main effect’, estimated leveraging the data at all ages) and (2) whether the association changed with age (the ‘interaction effect’; see Box 1). We detected significant (*P* < 0.05/3) positive main effects for all PGIs, and significant positive PGI × age interaction effects for all PGIs post-imputation and for PGI_EA_ pre-imputation (‘population model’ in Fig. 1a, and Supplementary Fig. 1a and Table 4). These findings suggest that the PGIs for education attainment and its cognitive and non-cognitive components explain increasingly more variance in IQ as children age.

These associations between children’s PGIs and IQ, which we term ‘population effects’, probably reflect a combination of direct genetic effects, genetic nurture and potential confounding due to population stratification and parental assortment^11–14^. We next sought to specifically estimate the direct genetic effects of PGIs on IQ across development. We repeated the above analyses adding parental PGIs as covariates in a subset of 4,968 unrelated parent–offspring trios for which we measured parental genotypes or could infer them via Mendelian imputation^17^ (‘trio model’, see Methods). In this model, the coefficient on the child’s PGI represents the direct genetic effects, while the coefficients on the parents’ PGIs represent the association between the child’s phenotype and alleles in the parents that are not transmitted to the child^11,17^. Our results for PGI_NonCog_ suggested that its population effect on IQ reflects genetic nurture or potential confounding, since we did not find evidence that the direct effect was significantly different from 0 (Fig. 1a, and Supplementary Fig. 1a and Table 4). In contrast, PGI_EA_ and PGI_Cog_ showed significant direct main effects on IQ, although weaker than the population effects (PGI_EA_ Δ*β* = 0.2090, *Z* = 7.19, *P* = 6.67 × 10^−^^13^, 95% CI [0.1520, 0.2660]; *Z*-test for difference between direct and population main effects; PGI_Cog_ Δ*β* = 0.1510, *Z* = 5.38, *P* = 7.35 × 10^−^^8^, 95% CI [0.0960, 0.2059]) (Fig. 1a and Supplementary Fig. 1a). The increasing associations of PGI_EA_ and PGI_Cog_ with age that we detected in the population model appear to reflect direct genetic effects, since the PGI × age interaction effects were significant and positive in the trio model and showed no evidence of statistical difference from the population effects (PGI_EA_ Δ*β* = 0.0148, *Z* = 0.843, *P* = 0.399, 95% CI [−0.049, 0.020]; *Z*-test for difference between direct and population interaction effects; PGI_Cog_ Δ*β* = −0.0258, *Z* = 1.554, *P* = 0.120, 95% CI [−0.058, 0.007]) (Fig. 1a and Supplementary Fig. 1a). The cognitive component of EA fully captured the direct genetic effect of PGI_EA_ on IQ: we found no evidence of significant differences between the direct effects for PGI_EA_ and PGI_Cog_ (Δ*β* = 0.0055, *Z* = 0.15, *P* = 0.881, 95% CI [−0.066, 0.077]; two-sided *Z*-test).

**Extended Data Fig. 2:**
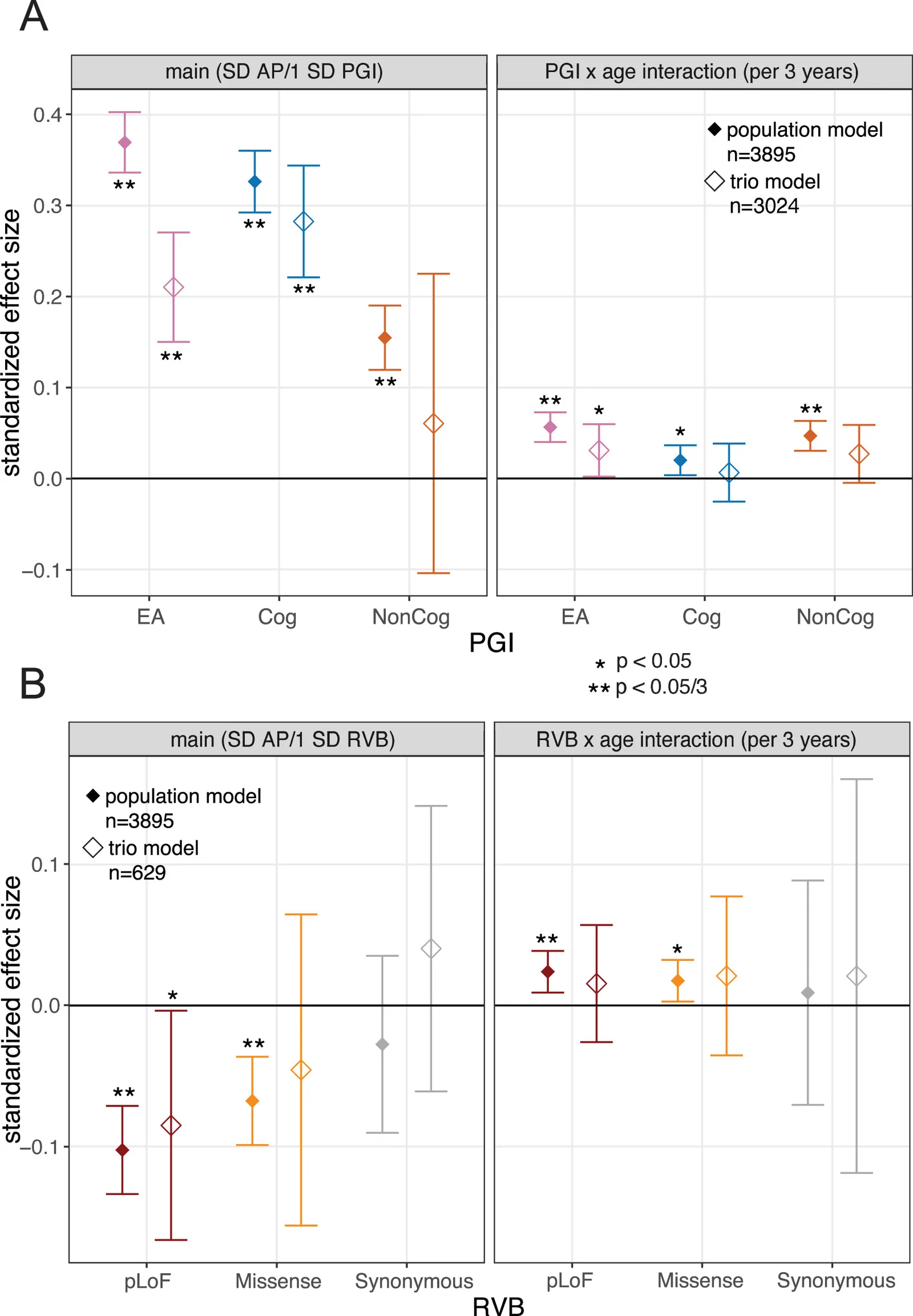
Association between PGIs and RVBs and academic performance in ALSPAC. The points show the Standardized effect sizes at age 11 (main effect, left panel) and the difference in effects between age 14 and 11 (genetic measure x age interaction, right panel) and error bars show 95% confidence intervals for **A**) PGIs and **B**) RVBs. Results are shown for the children’s genetic measures not controlling (‘population model’) and controlling (‘trio model’) for parental scores. Population effect sizes are shown estimated in the full sample. Effect sizes and p values were estimated using mixed-effects linear regression (two-sided Wald tests). Point estimates, 95% confidence intervals, and p values are provided in Supplementary Table 5.

In the trio model, all parental PGIs had significant main effects, indicating that genetic nurture and/or confounding captured by the parental PGIs are associated with childhood IQ (Fig. 1b, and Supplementary Fig. 1b and Table 4). Repeating these PGI analyses using a measure of academic performance in ALSPAC gave similar results (Supplementary Note 2, Extended Data Fig. 2a and Supplementary Table 5). When using a PGI constructed from an independent GWAS of cognitive performance^9^, the results yielded identical statistical interpretations (Supplementary Table 13 and Discussion). Notably, the genetic correlation between the cognitive component and a GWAS meta-analysis of cognitive performance^9^ was not statistically different from 1 (*r*_g_ = 1.02, *Z* = 30.76, *P* = 9.88 × 10^−^^208^, 95% CI [0.96, 1.09]; Linkage Disequilibrium Score Regression, test for *r*_g_ ≠ 1: *Z* = 0.60, *P* = 0.550).

Box 1 Terms used to describe genetic associations estimated in this study We estimate a variety of genetic associations from mixed-effects models (see ‘Associations between genetic measures and traits’ in Methods), including either main or interaction effects combined with either population effects, direct effects or effects of non-transmitted parental alleles. We note that our use of ‘effect’ in these terms does not necessarily imply causality, but we have used these terms in the strict statistical sense to be consistent with previous papers in the field.

**Population main effect**: the association between a child’s genetic score and the phenotype at the first measurement timepoint (that is, age 4 in the ALSPAC IQ analysis), but leveraging information from all timepoints to estimate this (see Methods). This association is estimated without adjusting for parental genetic scores in the regression and reflects the combined association of the child’s genetic score with their own phenotype, genetic nurture, parental assortment and uncontrolled population stratification.

**Population interaction effect**: the ‘change’ in the association between a child’s genetic score and their phenotype with age. For example, if the main effect is positive and the interaction effect is positive (as we found to be the case for PGIs on IQ), this indicates that the association with the genetic score increases with age, whereas if the main effect is negative and the interaction effect is positive (as we found for rare variant burden (RVB)), this indicates that the association attenuates with age.

**Direct main effect**: the association between a child’s genetic score and the phenotype at the first measurement timepoint, adjusting for the parental genetic scores (that is, a trio model). This association quantifies within-family prediction of the score, independently of genetic nurture, parental assortment and uncontrolled population stratification. Under simplifying assumptions, the trio model effect is equivalent to a within-sibship effect estimate.^17^

**Direct interaction effect**: the ‘change’ in the association between the child’s genetic score and their phenotype with age, while conditioning on parental genetic scores (that is, a trio model). The interpretation otherwise mirrors that of the population interaction effect.

**Non-transmitted coefficient:** the association between the parental genetic scores and the phenotype, estimated in the trio model; in this model, the direct effect of transmitted alleles is captured by the coefficient on the child’s score, whereas the coefficients on the parents’ scores are mathematically equivalent to the effect of the non-transmitted alleles in the parents.^17^ These associations reflect a combination of genetic nurture, parental assortment and uncontrolled population stratification.

**Fig. 2:**
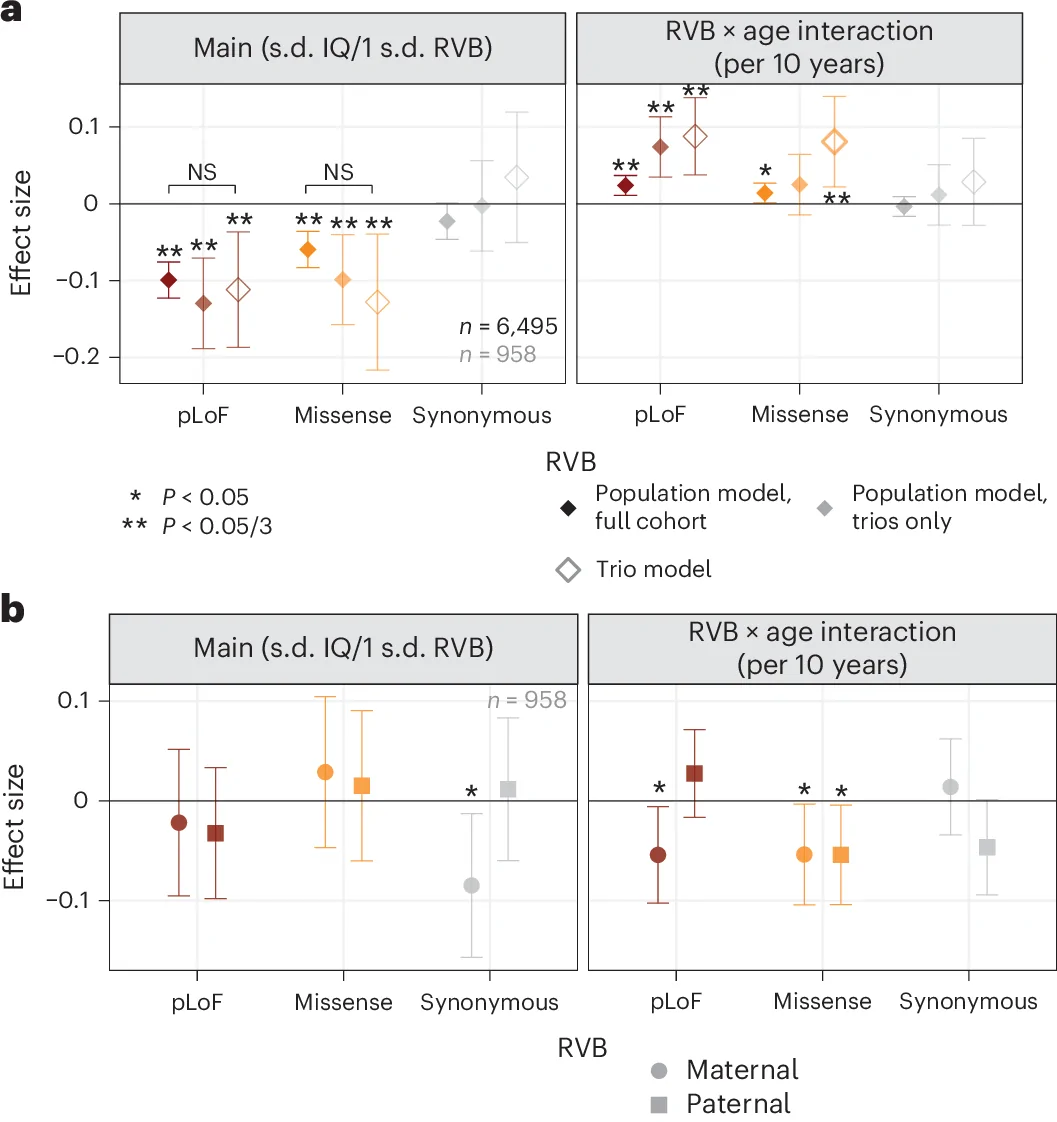
Association between rare variant burden (RVB) and IQ across ages. **a**, Standardized effect sizes and 95% CIs estimated for the main effects and RVB × age interaction effects for RVB on IQ (post-imputation), calculated with three different consequence classes (pLoF, missense or synonymous) (see Supplementary Fig. 2 for pre-imputation results.) Results for the children’s RVBs not controlling (that is, ‘population model’) and controlling (that is, ‘trio model’) for parental RVBs. Population effect sizes are shown estimated in the full sample (opaque; *n* = 6,495) and in the subset of children with parental RVBs (translucent; *n* = 958). **b**, Standardized effect sizes and 95% CIs for the parental RVBs from the trio model. Effect sizes and *P* values were estimated using mixed-effects linear regression (two-sided Wald test). The square brackets indicate comparisons highlighted in the text (two-tailed *Z-*test). The *P* values for the difference in population and direct main effect sizes are *P* = 0.758 and 0.108 for RVB_pLoF_ and RVB_Missense_, respectively. Point estimates, 95% CIs and *P* values are provided in Supplementary Table 6.

**Fig. 3:**
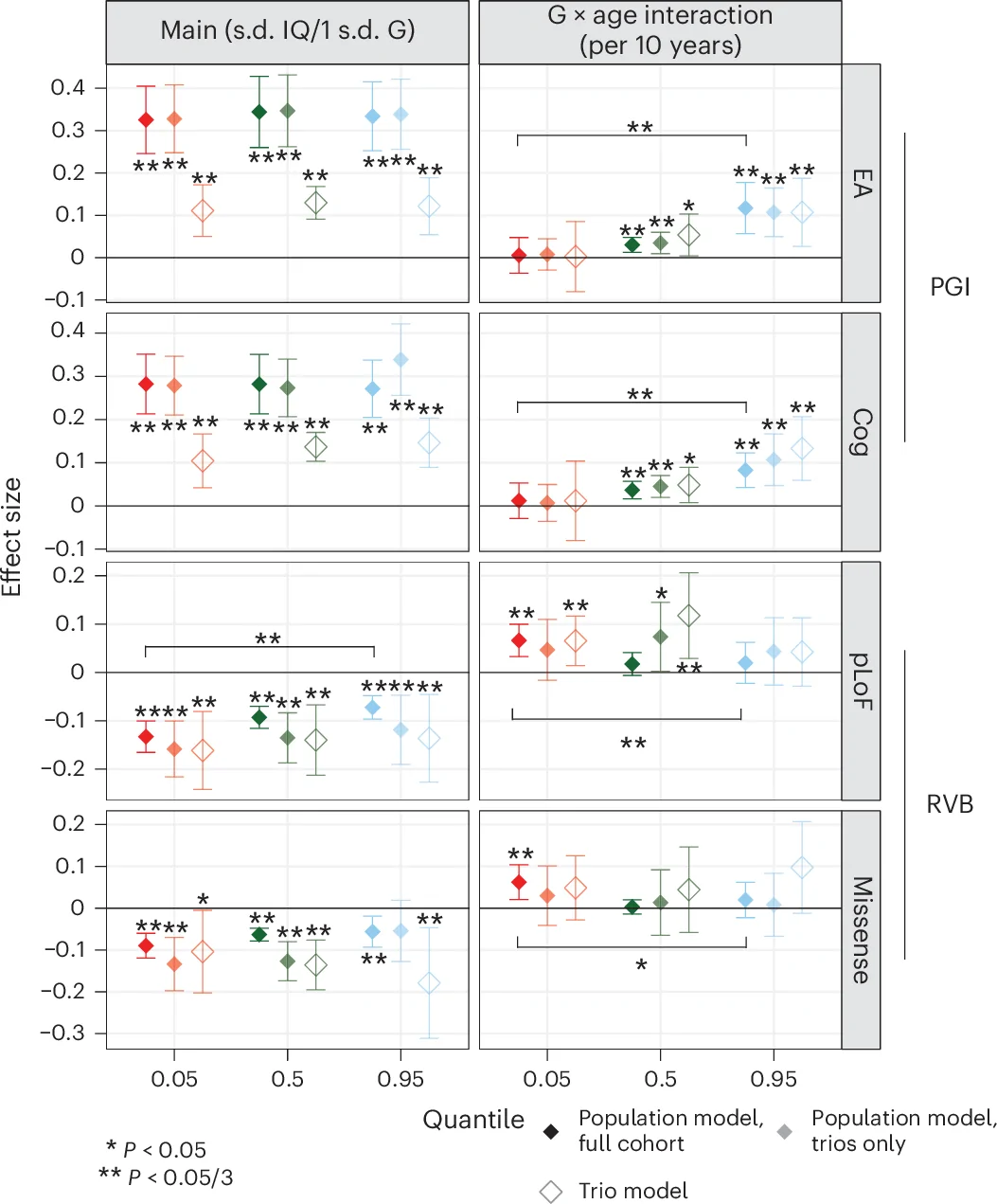
Association between common and rare variant measures and the tails of the IQ distribution. Standardized effect sizes and 95% CIs for quantile regression of the 5th, 50th and 95th percentiles estimated from mixed-effects modelling with post-imputation IQ at ages 4, 8 and 16 for PGI_EA_, PGI_Cog_, RVB_pLoF_ and RVB_Missense_ before and after controlling for parental genetic measures in the trio model (*n* (population models) = 6,495; *n* (trio model, PGIs) = 4,968; *n* (trio model, RVBs) = 958). See Extended Data Fig. 6 for a schematic motivating the effect estimates produced by quantile regression. See Supplementary Fig. 6 for results from this mixed-effects model pre-imputation, and Supplementary Figs. 5 and 7 for cross-sectional estimates at each age. Effect sizes and *P* values were estimated using mixed-effects quantile regression with bootstrapped standard errors (500 replicates, two-sided test). The square brackets indicate significant comparisons highlighted in the text (two-tailed *Z-*test). The *P* values for the difference in 5th and 95th percentile age interaction effect sizes are *P* = 0.003, 0.016, 0.012 and 0.043 for PGI_EA_, PGI_Cog_, RVB_pLoF_ and RVB_Missense_, respectively. The *P* value for the difference in 5th and 95th percentile main effect sizes for RVB_pLoF_ is *P* = 0.003. Point estimates, 95% CIs and *P* values are provided in Supplementary Table 8. Parental non-transmitted coefficients are shown in Extended Data Fig. 8.

**Fig. 4:**
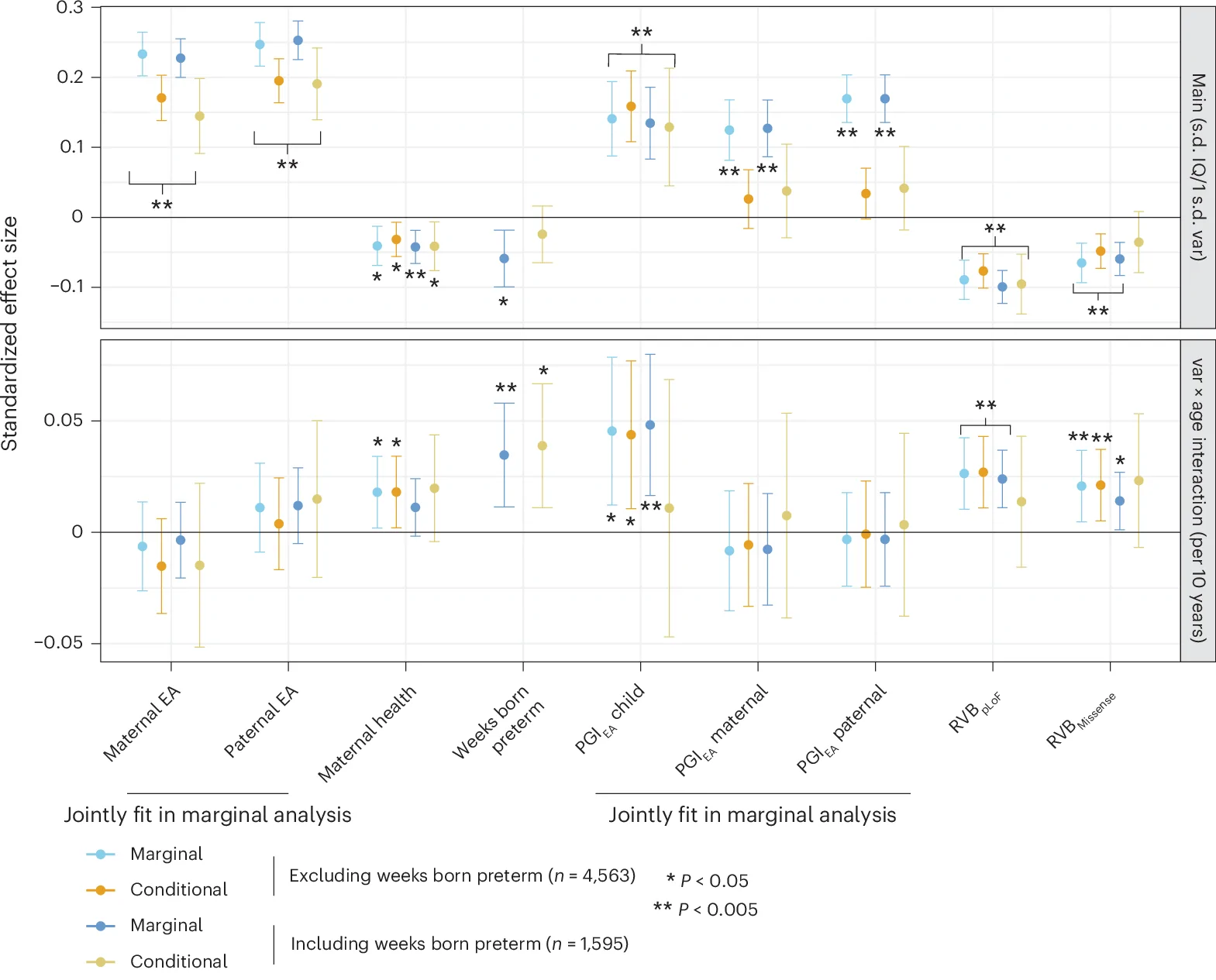
Associations between genetic and other factors and IQ across ages. Standardized effect sizes and 95% CIs for the main effects (top) and measure × age interaction effects (bottom) obtained from mixed-effects models fitted for IQ post-imputation. These were obtained from four models, as indicated in the key: marginal associations from models in which only the indicated variable(s) were included in the model (in addition to standard covariates), and conditional associations from models in which all the variables were included in the same model, allowing for direct comparison of associations e.g., the standardized main effects for the perinatal exposures were weaker than those for rare variants, with maternal illness and weeks born preterm explaining 0.18% and 0.35% of the variance in IQ at age 4 respectively, versus 1.40% for RVB_pLoF_ and RVB_Missense_ collectively (*P* = 5.2 × 10^−4^ and 0.027 *Z-*test, respectively). Note that we estimated the marginal and conditional effects either using only individuals who had gestational age information (*n* = 1,595) or all individuals (*n* = 4,563), dropping the ‘weeks born preterm’ variable in the latter case. Effect sizes and *P* values were estimated using mixed-effects linear regression (two-sided Wald test). See Methods for further details. Asterisks indicate whether the estimate is significantly different from 0 after Bonferroni correction for 10 tests (*P* < .005); note that square brackets with asterisks indicate that the estimates spanned by the bracket are all significant. See Supplementary Fig. 10 for an equivalent figure with results from quantile regression. Point estimates, 95% CIs and *P* values are provided in Supplementary Table 15.

Note that all of these associations are estimated both on the mean of the phenotype with linear regression (Figs. 1, 2 and 4), and also on quantiles of the phenotype (for example, the 5th percentile) with quantile regression (Fig. 3).

### Association between deleterious rare variant burden and cognitive performance across development

**Extended Data Fig. 3:**
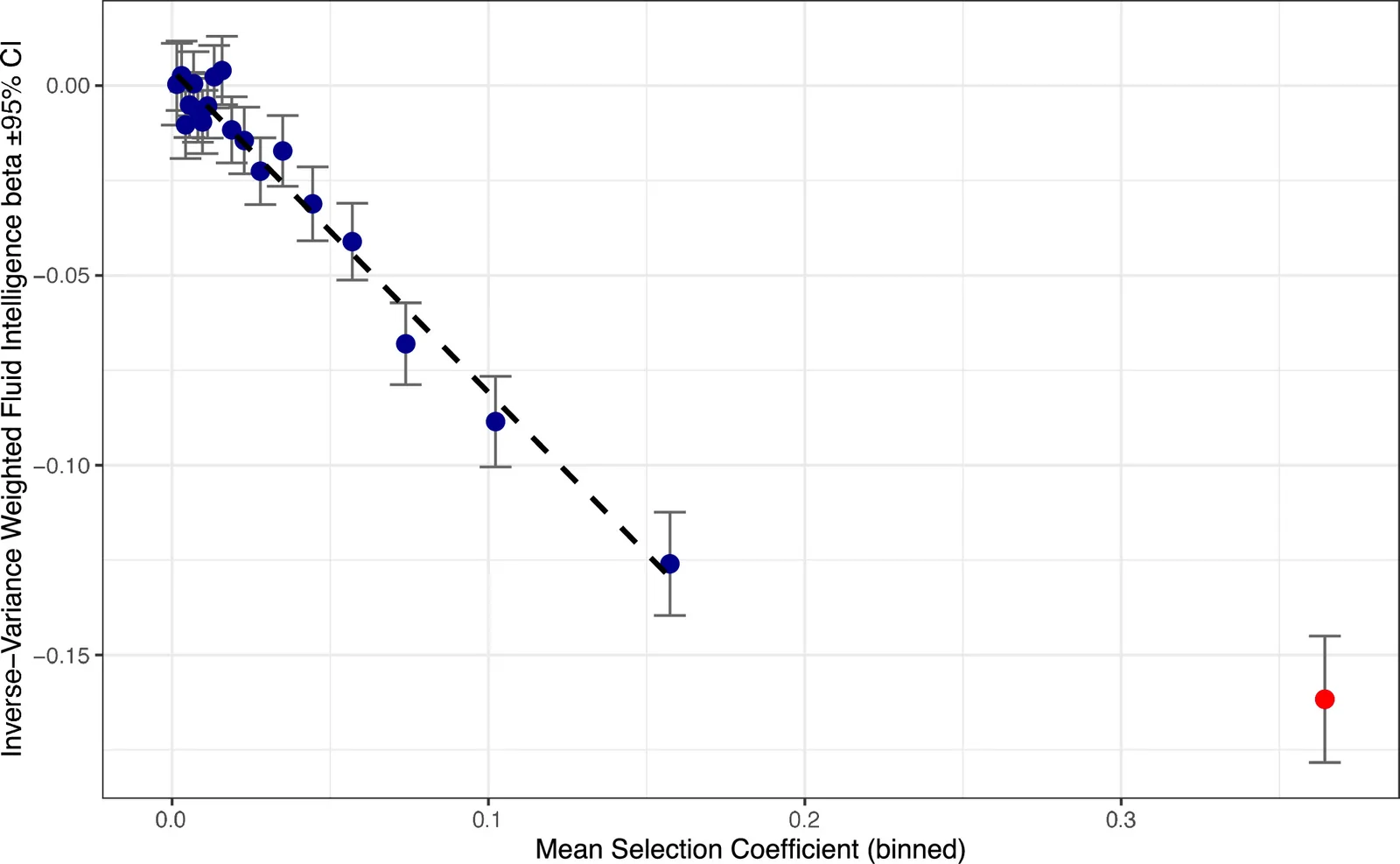
Association between gene-level selection coefficients and pLoF gene burden effect sizes on fluid intelligence. Shown are inverse-variance-weighted mean effect size estimates and 95% CI bands computed in 20 equal-count bins of genes (14,318 total genes, all bins had 71 genes with the exception of the last bin that had 69 genes). The bins were constructed using the gene selection coefficient estimates used as weights in the RVB measures, with each point representing one bin and the x-axis position being the mean selection coefficient for the genes in the given bin. The red dot marks the highest bin (excluded from the dashed regression line as it is an outlier), and the black dashed line is the best-fit linear trend across the remaining 19 bins (Deming regression slope -0.81 (95% CI [-0.963, -0.664]). The red dot is likely an outlier because pLoF variants in highly constrained genes found in UK Biobank are enriched for technical or annotation artefacts95, reducing their average effect size on cognition.

To query the associations between cognitive performance and rare protein-altering variants, we generated new exome sequencing data on ALSPAC (8,436 children and 3,215 parents) and MCS (7,667 children and 6,925 parents) to supplement the SNP genotype and longitudinal phenotype data already available^42^. We first used these to assess the associations between IQ and RVB in ALSPAC (*n* = 6,495). Current sample sizes are underpowered to estimate variant-level or even gene-level effect sizes on cognition for rare variants accurately^24,43^, so we cannot construct a rare variant score in the same way as we do for common variant-based PGIs. However, we noted that there is a strong, linear relationship between the degree of negative selection against loss-of-function (LoF) variants in a gene^44^ (‘constraint’) and the association between pLoFs in that gene and fluid intelligence in UK Biobank^43^ (*β* = −0.81, *Z* = −10.62, *P* = 2.42 × 10^−^^26^, 95% CI [−0.963, −0.664]; Deming regression) (Extended Data Fig. 3), suggesting that using the more precise measurement afforded by the selection coefficient is a reasonable variant weighting strategy. Thus, we quantified RVB as the sum of gene-specific selection coefficients^44^ for genes in which an individual carries a rare predicted LoF or deleterious missense variant (RVB_pLoF_, RVB_Missense_; minor allele frequency (MAF) < 0.1%; see Methods), and, as a negative control, calculated an equivalent score for synonymous variants (RVB_Synonymous_).

**Extended Data Fig. 4:**
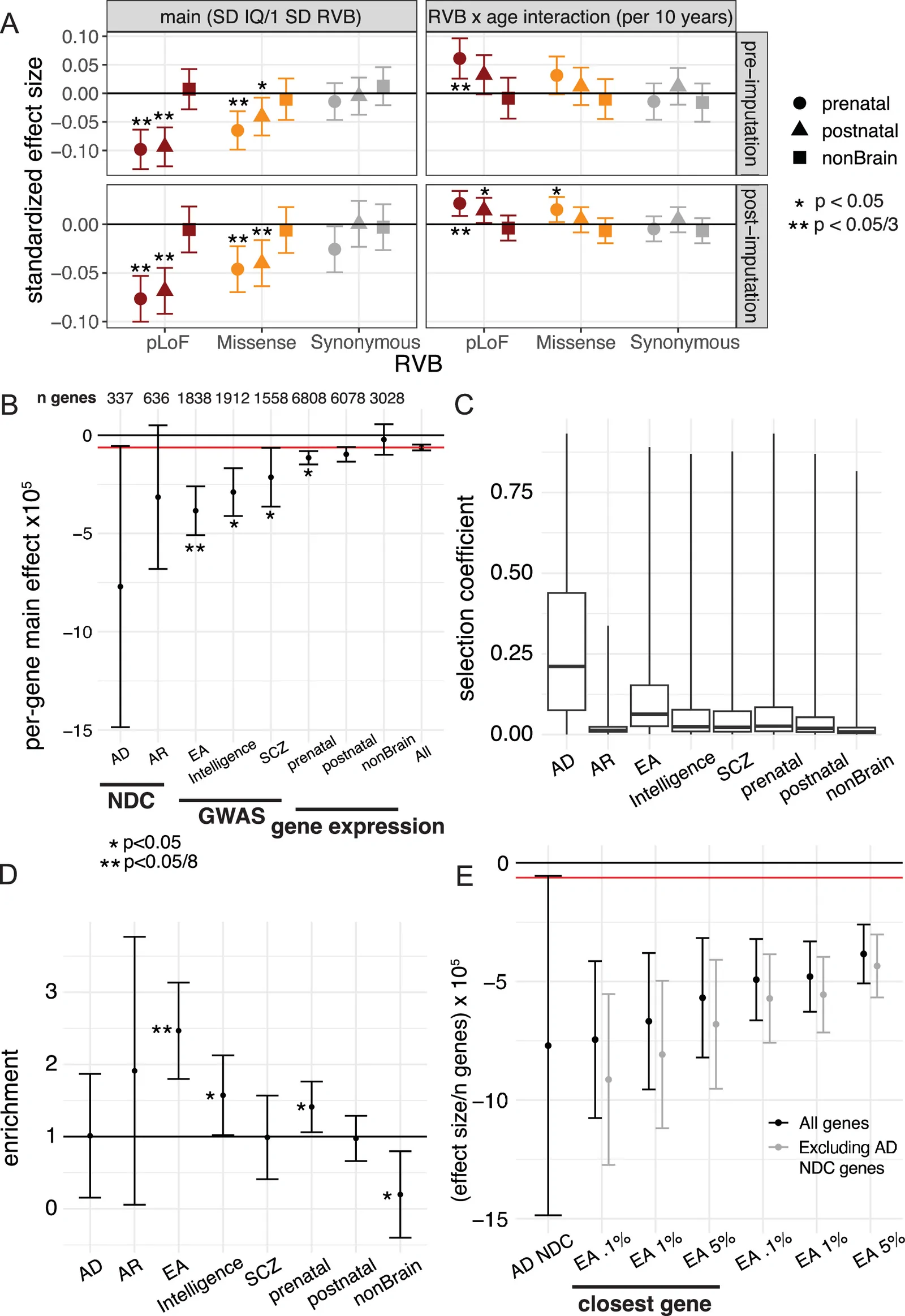
Associations and enrichment of gene set-specific RVB across development. **A**) Standardized effect sizes and 95% confidence intervals estimated for main effects and RVB-by-age-interaction effects for each RVB in three mutually exclusive gene sets from Li et al96. These comprise genes in co-expressed clusters that are more highly expressed in prenatal (6,808 genes) or postnatal brain (6,078) or that are not detected in the study (non-brain) (3,028)) pre- and post- imputation (pre-imputation ages 4 n = 1,012, 8 n = 7,347, and 16 n = 5,270 and n = 6,495 at all ages post-imputation, respectively). **B**) Standardized effect sizes for main effects of RVB_pLoF_ on IQ (post-imputation) stratified by gene set, divided by the number of genes in the given gene set with 95% confidence intervals. Red horizontal line indicates the average effect for RVB_pLoF_ across all genes. Asterisks indicate the p value for difference in per-gene effects between a given gene set and all genes using a two-sided z-test, with * indication nominal significance and ** indicating bonferroni significance for 8 tests. **C**) Boxplots of the distribution of shet (selection coefficient against heterozygous pLoFs in that gene^44^) per gene set, where a coefficient of 0 indicates no selection against heterozygous pLoFs in a given gene and 1 indicates a 100% reduction in fitness for heterozygous pLoF carriers relative to non-carriers. Box plots show the minimum, 1st quartile, median, third quartile, and maximum values. **D**) Ratio of the main effect for RVB_pLoF_ for the indicated gene set relative to randomly sampled gene sets with matching underlying shet distributions (enrichment) with 95% confidence intervals. **E**) As in (**B**) for gene sets defined using different FDR threshold cutoffs based on gene prioritization in 97 (5%, 1%, 0.1%) and by restricting to prioritized genes at a given cutoff that are also the closest genes to the prioritizing SNP in the Lee et al. GWAS with 95% confidence intervals (EA < 5% n = 1,838, 1,711, and 830 for all genes, excluding NDC, and further restricting to closest gene, respectively, EA < 1% n = 1,536, 1,418, and 721, EA < 0.1% n = 1,327, 1,216, and 621). Results are shown before (black) or after (grey) excluding overlapping genes from the set of autosomal dominant neurodevelopmental condition genes with a loss-of-function mechanism from DDG2P (AD NDC). AD/AR NDC (337 and 636 genes, respectively): Autosomal dominant/recessive neurodevelopmental disorder genes with loss-of-function mechanism from DDG2P^97^, EA (1,838): educational attainment GWAS prioritized genes by Lee et al.^91^ at 5% FDR threshold, Intelligence (1,912): intelligence GWAS prioritized genes from Savage et al.^9^, SCZ (1,558): schizophrenia GWAS prioritized genes from Pardiñas et al.^92^ Effect sizes and p values were estimated using mixed-effects linear regression (two-sided Wald tests). Point estimates, 95% confidence intervals, and p values are provided in Supplementary Table 14.

RVB_pLoF_ and RVB_Missense_ were significantly negatively associated with IQ, whereas RVB_Synonymous_ was not (Fig. 2a left, and Supplementary Fig. 2a and Table 6). The unstandardized effect size of RVB_pLoF_ was 3.2-times larger than that of RVB_Missense_ (Δ*β* = −0.757, *Z* = −5.07, *P* = 3.94 × 10^−^^7^, 95% CI [−1.050, −0.464]; two-sided *Z-*test) (Supplementary Fig. 3). The association between IQ and deleterious rare variants attenuated with age (Fig. 2a, and Supplementary Fig. 2a and Table 6). Analyses using an academic performance measure in ALSPAC yielded similar results (Supplementary Note 2). Gene set-specific analyses indicate that genes prioritized by common variant analyses of EA and genes expressed in pre- and post-natal brain make an outsized contribution to the RVB associations with IQ (Supplementary Note 3 and Extended Data Fig. 4).

We then estimated the direct genetic effects of RVBs on IQ across development in 958 unrelated exome-sequenced trios, by adding parental RVBs as covariates. Our findings suggested that the population effects of rare variants are dominated by the direct effects, in contrast to what we observed for PGIs: there was no evidence that the estimated population and direct main effects were significantly different from each other for either RVB_pLoF_ or RVB_Missense_ (RVB_pLoF_ Δ*β* = −0.043, *Z* = −0.84, *P* = 0.399, 95% CI [−0.144, 0.057], two-sided *Z-*test; RVB_Missense_ Δ*β* = −0.096, *Z* = −1.61, *P* = 0.108, 95% CI [−0.214, 0.021]) (Fig. 2a, and Supplementary Fig. 2a and Table 6) and no evidence of parental RVB measures being significantly associated with IQ after accounting for multiple testing (Fig. 2b, and Supplementary Fig. 2b and Table 6), although this may in part be due to the smaller sample size. We also found that the direct effects of both RVB_pLoF_ and RVB_Missense_ significantly attenuated with age (Fig. 2a, and Supplementary Fig. 2a and Table 6). In summary, our results show that higher exome-wide burden of rare damaging variants is associated with lower IQ in childhood, although with attenuated effects at later age, and that this pattern is recapitulated when examining the direct genetic effects.

**Extended Data Fig. 5:**
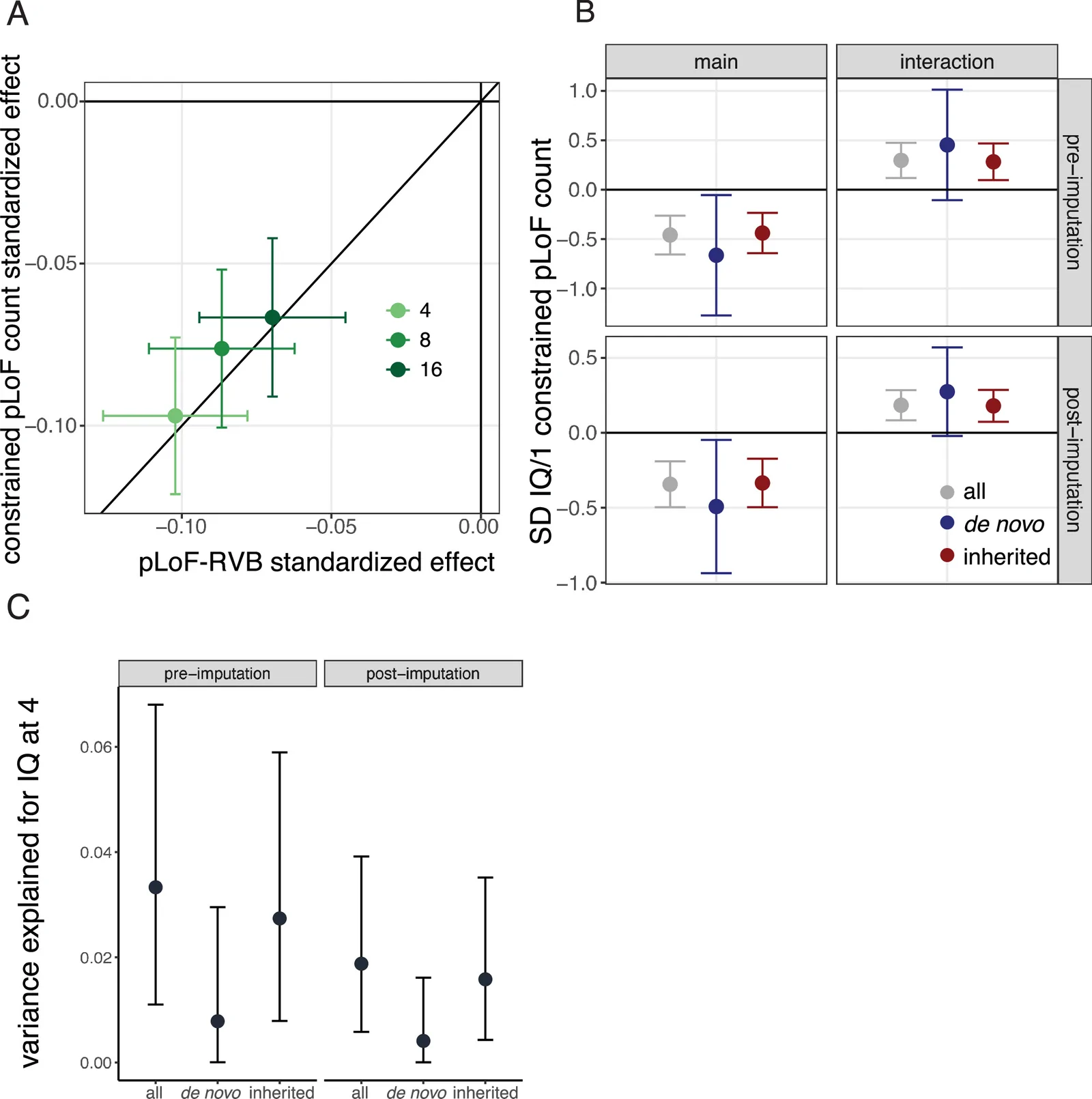
Association between inherited and de novo pLoF variant counts in constrained genes and IQ in ALSPAC. **A**) Standardized effect sizes and 95% confidence intervals of RVB_pLoF_ and constrained pLoF count (see Supplementary Methods) on IQ across ages post-imputation (n = 6,945). Black line indicates the y = x line. **B**) Standardized effects and 95% confidence intervals for the main and RVB-by-age interaction effect from a longitudinal mixed-effects model of constrained pLoF counts on standardized IQ when considering all variants or *de novo* mutations and inherited variants separately, both pre- (age 4 n = 154, age 8 n = 861, age 16 n = 895) and post-imputation (n = 958). **C**) Variance explained and 95% confidence intervals by constrained pLoF counts on standardized IQ when considering all variants or *de novo* and inherited separately, both pre- and post-imputation. Effect sizes and p values were estimated using mixed-effects linear regression (two-sided Wald tests). See Supplementary Figure 1 for results from a similar analysis of cognitive performance in MCS. Point estimates, 95% confidence intervals, and p values are provided in Supplementary Table 6.

When partitioning the association between IQ and rare pLoFs in constrained genes into inherited variants versus de novo mutations, we estimated that the former explained 3.5-times more variance in IQ than the latter in ALSPAC (Δ*β* = 0.076, *Z* = 2.03, *P* = 0.042, 95% CI [0.003, 0.149]; two-sided *Z-*test) (Supplementary Note 4 and Extended Data Fig. 5), and we saw similar results when considering a composite measure of childhood cognitive performance in MCS (see Methods and Supplementary Fig. 4). This contrasts with the relatively larger contribution of de novo mutations in cohorts of children with neurodevelopmental disorders^19,45^.

### Association between genetic factors and the tails of the IQ distribution

**Extended Data Fig. 6:**
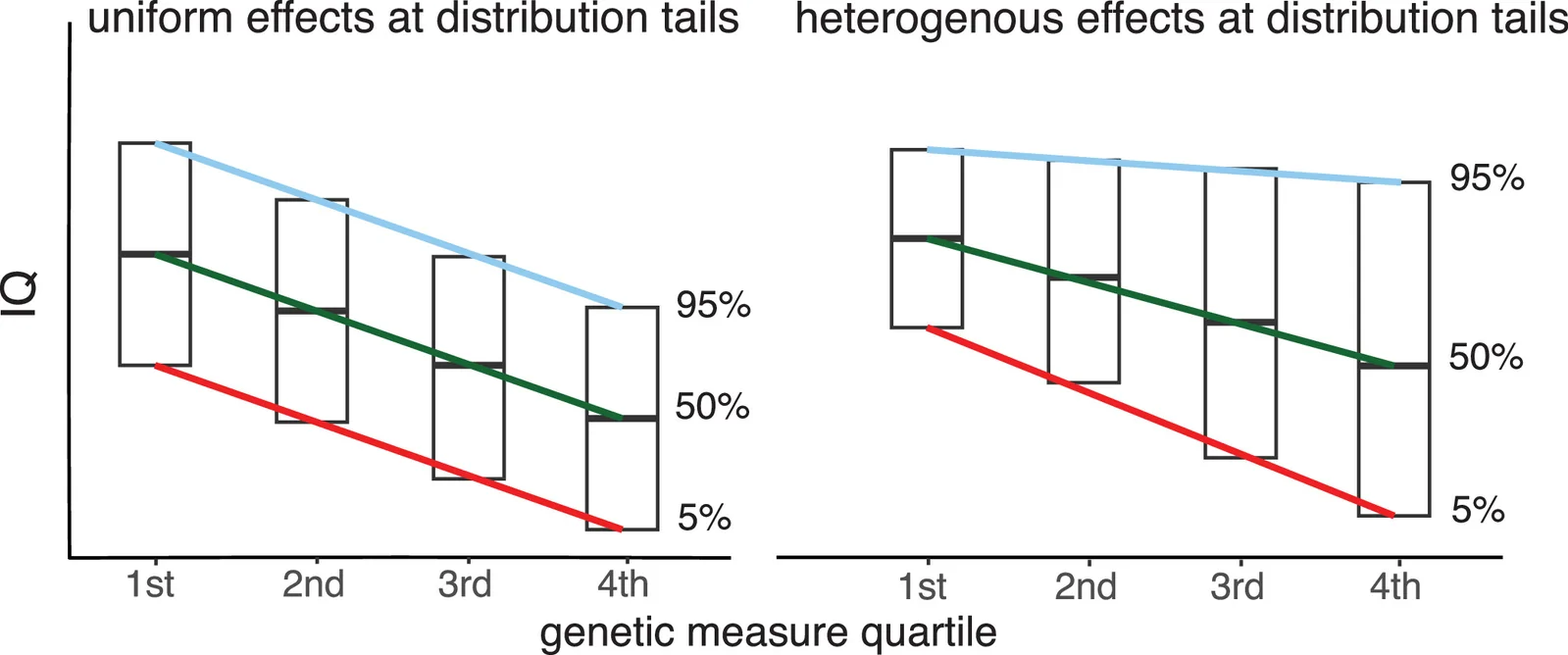
Schematic illustrating the difference between homoskedasticity and heteroskedasticity in terms of their effects on the relationship between a phenotype (IQ) and a genetic measure with which it is associated. The boxes show the distribution of the phenotype for individuals in the indicated quartile of the genetic measure (with the three bars representing the 5th, 50th and 95% percentile respectively), and the red, green and blue lines indicate the association between the genetic measure and the phenotype at those three quantiles. In the lefthand scenario, the genetic measure has uniform effects across the IQ distribution, and hence the coloured lines are parallel. In the righthand scenario, the genetic measure has heterogeneous effects on IQ across the IQ distribution, with a larger effect on the 5th percentile than the 50th or 95th. This righthand scenario is what we observed for RVB_pLoF_ and RVB_Missense_ at age 4 (assuming that the 4th quartile represents the highest RVB_pLoF_ score), which show larger effects on the 5th than 50th or 95th percentile (Supplementary Figure 4).

Our results thus far consider genetic associations with mean IQ. Deleterious rare variants in constrained genes are known to be related to risk of neurodevelopmental conditions^19^, hinting that they may possibly have larger associations with the lower tail end of the IQ distribution. This would reflect a statistical phenomenon known as heteroscedasticity, in which, in a linear regression of phenotype on genotype, the variance of the residuals varies by genotype. To test whether there are heterogeneous genetic associations with the tails of the IQ distribution, we used quantile regression to estimate the association between PGIs and RVBs and both the median IQ as well as the bottom and top 5th percentiles (Extended Data Fig. 6), which roughly correspond to IQs of 75 and 125, respectively^35^.

**Extended Data Fig. 7:**
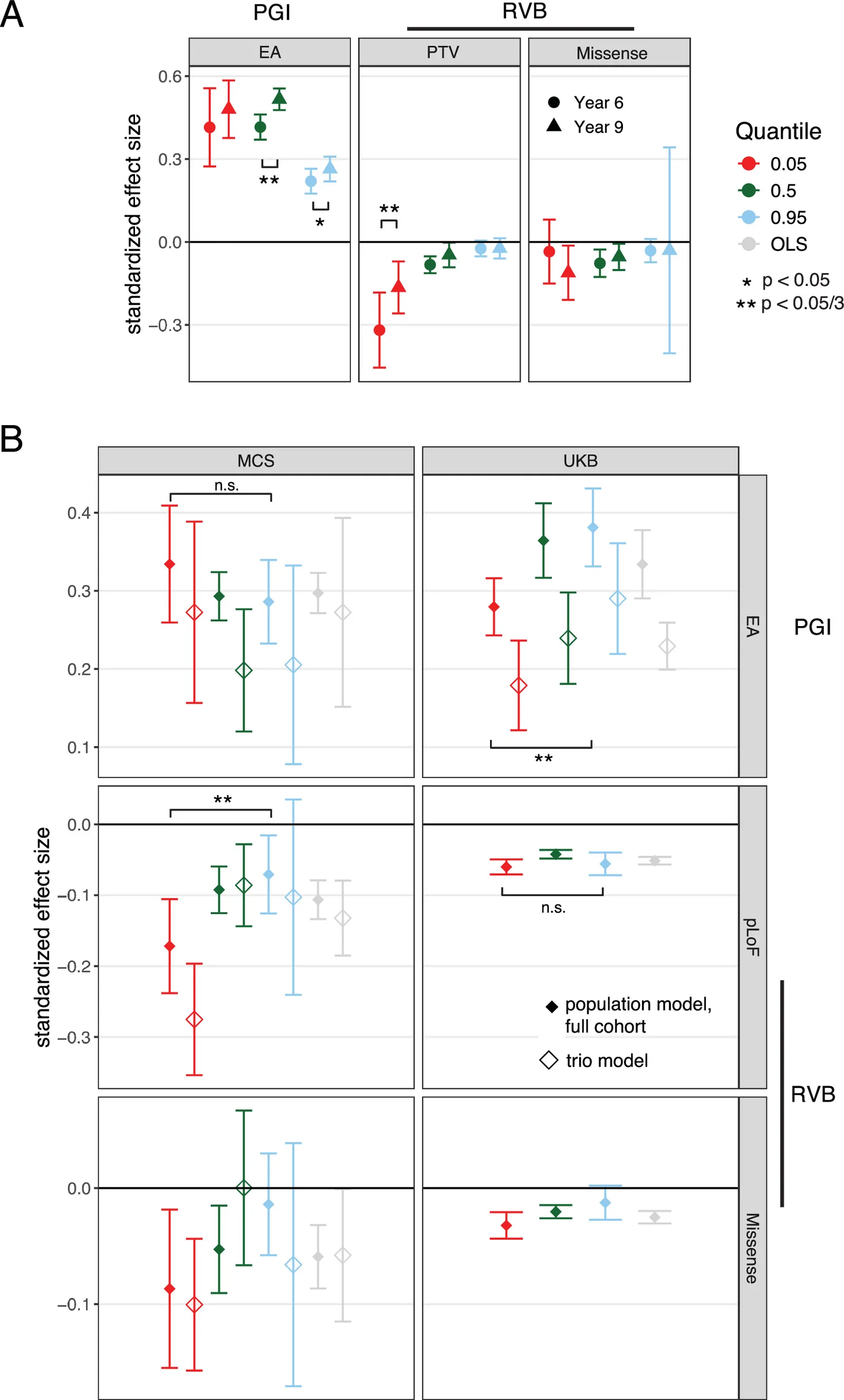
Influence of genetic measures on the tails of the phenotypic distribution using academic performance in ALSPAC and cognitive performance measures in MCS and UK Biobank. **A**) Standardized effect sizes and 95% confidence intervals for quantile regression of the 5th (red), 50th (green), and 95th (blue) percentiles of genetic measures on academic performance in Year 6 and Year 9 in the full ALSPAC cohort showing population effects (n = 3,895). The square brackets with asterisks indicate significant age interactions. **B**) Standardized effect sizes and 95% confidence intervals for quantile regression of the 5th (red), 50th (green), and 95th (blue) percentiles and linear regression (OLS; gray) estimated from cross-sectional associations with cognitive performance measures in MCS (n = 5,920 and 5,309 population and direct effect n for PGI analyses, respectively, n = 5,666 for RVB analyses) and UK Biobank (n = 101,232 and 11,859 population and direct effect n, respectively, n = 101,232 for RVB analyses). Effect sizes and p values were estimated using mixed-effects quantile regression with bootstrapped standard errors (500 replicates; two-sided tests). The PGI_EA_ used in UK Biobank was constructed from an EA GWAS in 23andMe only^13^, as the GWAS excluding 23andMe includes UK Biobank. We only considered PGI_EA_ as we could not construct PGI_Cog_ to apply in UK Biobank since we did not have an independent GWAS for cognitive ability in adults that excluded this cohort. The square brackets indicate significant comparisons highlighted in the text (two-sided z tests). Point estimates, 95% confidence intervals, and p values are provided in Supplementary Table 9.

First, we considered PGI_EA_ and PGI_Cog_, which had significant direct genetic effects on IQ in Fig. 1. Our results suggested that the observed increase in these PGI associations with IQ with age was due to increasing associations in the upper half of the IQ distribution (Fig. 3). Although the associations between the PGIs and these different quantiles in the IQ distribution were largely concordant at any given timepoint (Supplementary Fig. 5 and Table 8), we found significant positive age interaction effects for both PGIs at the median and 95th quantile for population effects, although these did not all survive correction for multiple testing when considering the direct effects (Fig. 3 and Supplementary Table 8). There was no evidence for significant age interactions at the 5th percentile for either PGI. Concordant results were seen when analysing academic performance in ALSPAC and cognitive ability measures in MCS and UK Biobank (Supplementary Note 5, Extended Data Fig. 7 and Supplementary Table 9).

Second, we considered the association between rare variant burden and the different quantiles of the IQ distribution at different ages in ALSPAC. Broadly, we found that the associations with deleterious rare variants were strongest on those with low IQ, and that these associations attenuate more markedly with age than those on the upper tail of the distribution, reducing the heterogeneity in associations across the phenotype distribution with time (Supplementary Fig. 7b and Table 8). Notably, at age 4, the population effect of RVB_pLoF_ at the 5th percentile was 3.9-times greater than that at the 95th percentile (Δ*β* = −0.159, *Z* = −3.91, *P* = 9.23 × 10^−^^5^, 95% CI [−0.239, −0.079]; two-sided *Z-*test) and 2.2-times greater than at the median (Δ*β* = −0.118, *Z* = −4.07, *P* = 4.69 × 10^−^^5^, 95% CI [−0.175, −0.061]), whereas by age 16, there was no evidence that the estimates at the different quantiles were significantly different from one another (Δ*β* = −0.023, *Z* = −0.57, *P* = 0.5672, 95% CI [−0.100, 0.055]). Put another way, this implies that, at age 4, the variance in IQ in people with high RVB_pLoF_ is greater than in those with lower RVB_pLoF_, as illustrated in the right-hand plot in Extended Data Fig. 6; however, by age 16, RVB_pLoF_ shows uniform effects at the distribution tails. As a consequence of this, the significant attenuation of population effects with age was observed only at the 5th percentile and median, but not at the 95th percentile, for both RVB_pLoF_ and RVB_Missense_ (Fig. 3). This finding was recapitulated when examining direct genetic effects of RVB_pLoF_, although not of RVB_Missense_ (Fig. 3). Analyses of school grades in ALSPAC and of cognitive ability measures from MCS and UK Biobank yielded concordant observations (Supplementary Note 5 and Extended Data Fig. 7).

We note that our results here imply that there is heteroscedasticity (that is, the variance of the phenotype varies with genotype), which could violate assumptions regarding the estimation of standard errors in our mixed-effects models in Figs. 1 and 2. However, re-estimating several of our key models with bootstrap-derived standard errors produced virtually identical results (Supplementary Tables 4 and 6), suggesting that any heteroscedasticity had minimal impact on our earlier analyses. We do note that there were some differences in pre- and post-imputation results for some of the quantile regression associations with PGIs, which are described in detail in Supplementary Note 7. We also conducted robustness analyses to assess the impact of attrition on these results, and found that it is unlikely that the quantile regression associations could be solely artefacts of attrition (Supplementary Note 8).

**Fig. 5:**
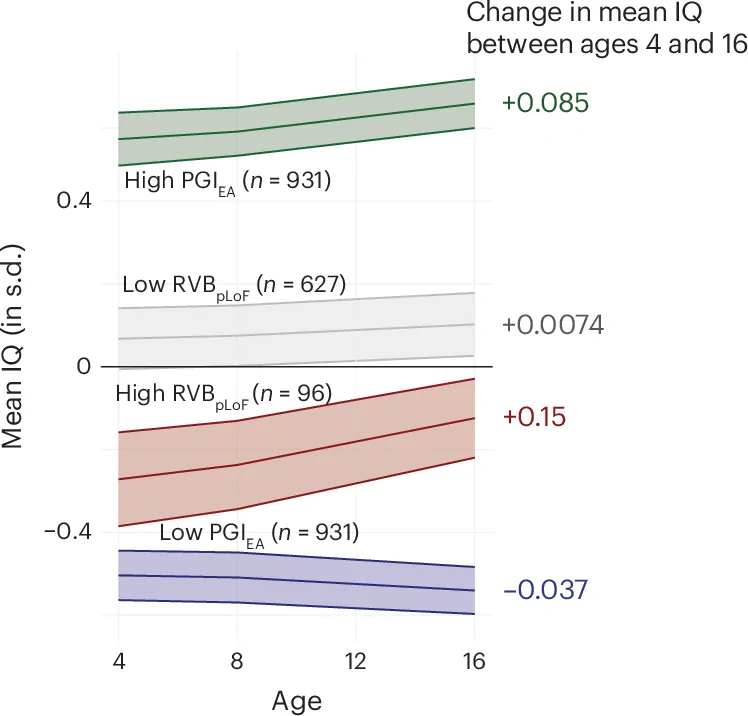
Summary of phenotypic trajectories across development, as inferred in this work. Average trajectories of IQ (inferred post-imputation) for individuals grouped by genetic measures. The high/low PGI_EA_ groups are individuals with a value that is one or more standard deviations above/below 0. The low RVB_pLoF_ group are individuals with RVB_pLoF_ below 0.01 (9.6% of individuals) and the high RVB_pLoF_ group are individuals with RVB_pLoF_ greater than 0.4 (1.5% of individuals); these cut-offs were chosen arbitrarily for illustrative purposes. The change in mean genetic measure/IQ between ages 4 and 16 are shown on the right-hand side of the plots. Bands indicate 95% confidence intervals. The decrease in mean IQ in the low PGI_EA_ group is explained by the fact that, due to PGI_EA_ effects becoming stronger towards the top of the IQ distribution, those that had a high IQ earlier in development will tend towards their genetically predicted lower IQ at later ages, thereby lowering the average IQ in this group.

In summary, we show that the negative associations with RVBs are strongest at the lower tail of the distribution of cognitive ability, and that these associations attenuate with age. However, for the PGIs for education attainment and its cognitive component, the significant age interactions were seen only in the top half of the IQ distribution. These patterns are illustrated in Fig. 5.

### Relative contribution of genetic and other exposures to IQ

**Extended Data Fig. 9:**
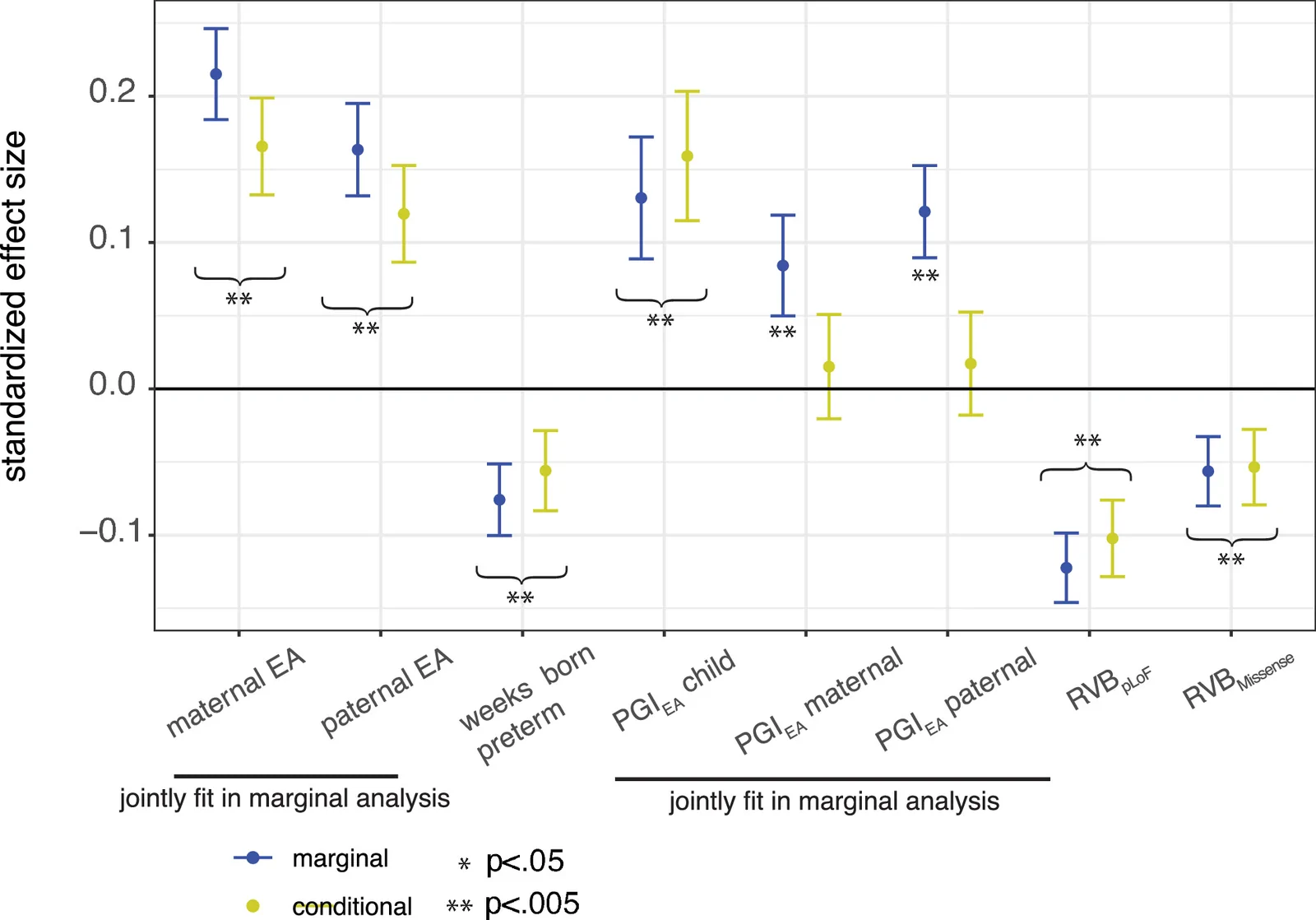
Associations between genetic and other factors and cognitive performance across ages in MCS. Standardized effect sizes and 95% confidence intervals from obtained from two linear models (n = 5,219), as indicated in the key: marginal associations from models in which only the indicated variable(s) were included in the model (in addition to standard covariates), and conditional associations from models in which all the variables were included in the same model. Effect sizes and p values were estimated using linear regression (two-sided Wald tests). Point estimates, 95% confidence intervals, and p values are provided in Supplementary Table 15.

Finally, we compared the longitudinal associations of PGI_EA_, RVB_pLoF_ and RVB_Missense_ with IQ in ALSPAC (Figs. 1 and 2) to those with other factors that have previously been associated with children’s academic and cognitive outcomes (see Methods): realized parental educational attainment and two perinatal factors, namely, maternal illness during pregnancy and premature birth (number of weeks born preterm)^46–48^. The results from this analysis are described in detail in Supplementary Note 6 and summarized briefly here. When fitting the variables separately (marginal associations in Fig. 4), we found strong positive associations with parental EA, as well as negative associations with perinatal factors (Supplementary Table 15), consistent with previous findings^49,50^. The negative effect of premature birth attenuated across development (Fig. 4 and Supplementary Table 15). In a joint model including parental EA, perinatal exposures and genetic scores (offspring and parental PGI_EA_ and the child’s RVB_pLoF_ and RVB_Missense_), most effect sizes changed minimally compared to the marginal associations. One exception was that the parental PGI_EA_ associations became non-significant, consistent with ref. ^51^. Importantly, we did not observe evidence of significant change in the associations of the child’s genetic scores (Fig. 4 top and Supplementary Table 15). RVB contributions were broadly comparable to those of the perinatal exposures, and the direct effect of the child’s PGI_EA_ was similar to the effect of parental EA. Conclusions were similar when considering a composite measure of childhood cognitive performance in MCS (Extended Data Fig. 9 and Supplementary Table 15).

We further considered the association between the different quantiles of the distribution and parental EA and these perinatal exposures (Supplementary Fig. 10). Our conclusions from the marginal analysis in Fig. 3 remain unchanged when controlling for these other factors. Taken together, our results indicate that the relative associations of factors that best predict which children will have cognitive difficulties or will excel cognitively across childhood differ from those that best predict average IQ.

## Discussion

Here we explored the association between different genetic scores and cognitive performance longitudinally across early life. We showed that RVB differs in its pattern of association with IQ and school achievement over time compared to PGIs for educational attainment and its cognitive and non-cognitive components. While the association with RVB_pLoF_ and RVB_Missense_ attenuates between childhood and adolescence (Fig. 2), the association with PGI_EA_ and PGI_Cog_ increases (Fig. 1). In both cases, these age interactions are due to direct genetic effects, with no evidence for an age interaction of non-transmitted common or rare alleles in parents (Fig. 1b); there was also no evidence for associations between parental RVB and average IQ in trio models (Fig. 2b) (see Supplementary Discussion for potential explanations). Our PGI findings accord with those of Malanchini et al. who studied academic achievement in a different cohort^52^. When comparing the relative magnitude of the main effects of genetic versus other factors on IQ in ALSPAC, we found that the RVB contribution was broadly comparable to those of the perinatal exposures, and the direct effect of PGI_EA_ was comparable to that of parental EA (Fig. 4 top). This highlights the important contribution of genetic variation across the allele frequency spectrum to variance in cognitive performance.

In theory, the increasing associations between IQ and polygenic indices for educational attainment and its cognitive component with age could simply be because the underlying SNP effects have been estimated on adult phenotypes which are better correlated with IQ in later childhood than in early childhood^53,54^. However, since there was no evidence that common variant genetic correlations between IQ measured at the different ages were significantly different from 1 (Supplementary Table 2), it is more likely that this association is explained by a true increase in common variant heritability^55^ (Supplementary Discussion). Our finding that the association with RVBs attenuate as children age cannot be explained by the way the variant weights were obtained; this is because the weights are based on estimates of evolutionary negative selection against deleterious variants in each gene in large population-based cohorts of adults^44^, rather than through genotype–phenotype association studies. Since the construction of the common variant-based PGIs and the RVBs differ, the constructs are technically not directly comparable, so one should be cautious about drawing strong conclusions about differences between their patterns of association.

**Extended Data Fig. 10:**
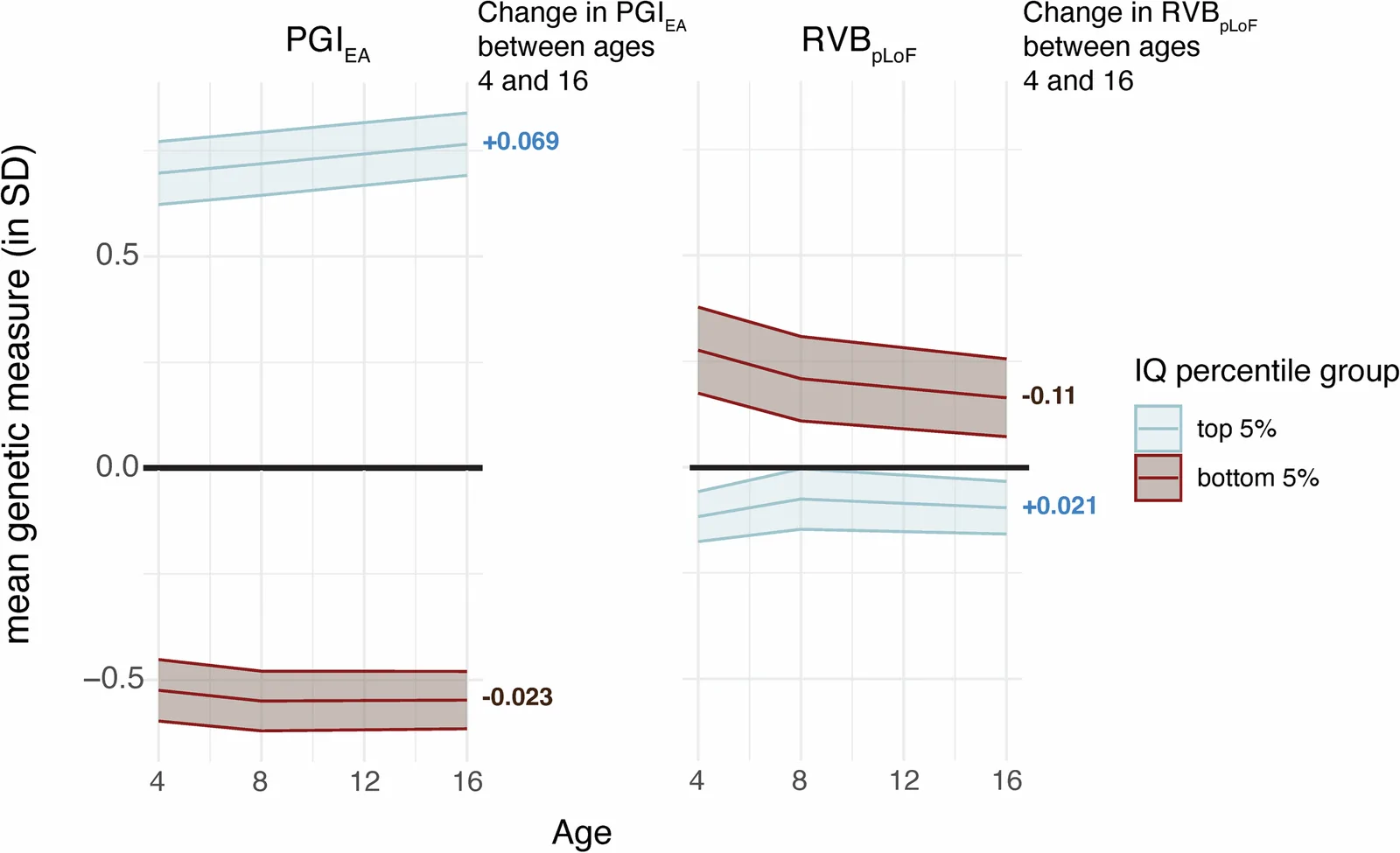
Summary of genetic trajectories across development, as inferred in this work. Mean and 95% confidence intervals for PGI_EA_ (left) and RVB_pLoF_ (right) for individuals in the top (light blue) or bottom (brown) 5% percentile of IQ at a given age (inferred post-imputation of IQ). Note that the individuals in the top and bottom 5th percentile groups vary across development. Children at the top 5th percentile of IQ at age 16 have a higher mean PGI_EA_ than those at the top of the distribution at age 4, while those at the bottom 5th percentile have a relatively steady mean PGI_EA_. In contrast, the quantile-specific age interactions for RVB_pLoF_ suggest that children in the bottom 5th percentile of IQ at age 4 have a much higher burden of damaging rare variants than those at age 16, while those at the top 5th percentile have a stable and low rare variant burden.

Genetic studies typically test for genetic associations with the mean of the phenotype, although some have also considered associations with variance^56–60^ or differential associations with different quantiles of anthropometric traits and biochemical measures^61^. Here we examined the associations of PGIs and RVBs with different quantiles of the IQ distribution, which essentially tests for an association with the phenotypic variance and skew. The increasing associations between PGI_EA_/PGI_Cog_ and mean IQ with age were strongest for the top half of the phenotype distribution, whereas the attenuating associations with RVB_pLoF_ were strongest in the lower tail (Fig. 3). The latter observation appears to reflect RVB_pLoF_ having a larger association with the 5th than the 95th percentile of IQ in early childhood, but similar associations with the distribution tails by age 16 (Supplementary Fig. 7). Results from other cohorts were concordant with these observations (Extended Data Fig. 7). These results imply that the genetic profiles of children in the highest and lowest ranges of cognitive ability differ across development (Extended Data Fig. 10). Figure 5 summarizes our key findings: children in the high versus low PGI_EA_ groups become more stratified across development, with more of the stratification occurring due to increasing IQ in the high PGI_EA_ group, while the stratification in IQ due to RVB_pLoF_ is strongest at the earlier ages and then attenuates, particularly in the high RVB_pLoF_ group.

Our results imply that there is higher variance in IQ in early childhood among individuals with higher RVB_pLoF_, which could potentially be due to gene × environment or gene × gene effects, or simply to stochasticity. More people with high RVB_pLoF_ may cross the diagnostic threshold for intellectual disability than among those with low RVB_pLoF_, but since the association is non-uniform, it may be that many individuals with high RVB_pLoF_ would not cross the threshold, consistent with variable and reduced penetrance of these variants. Multiple neurodevelopmental conditions exhibit a high degree of phenotypic variability, even among carriers of the same causal mutation, such as in neurofibromatosis type 1 (ref. ^62^). Although quantitative measures of intellectual impairment are relatively rare in studies of these conditions, there is evidence, for example, that patients with tuberous sclerosis complex (TSC) have not only lower mean IQ but also higher variance in IQ than unaffected sibling controls (*F*-test for difference in variance between cases and controls; *P* = 0.004 for TSC1)^63^. Taken together, our results support two hypotheses for the incomplete/variable penetrance of rare NDC-associated variants. First, these variants are associated with increased variance in cognitive ability early in life, consistent with heterogeneous phenotypes of children with NDCs. Second, they have larger effects on average cognitive ability in early childhood than later in life, consistent with apparently clinically unaffected parents passing on these pathogenic variants to affected children.

There may be social and/or biological explanations for our observation that RVB associations attenuate with age whereas PGI associations do not. For example, unlike PGIs, RVBs associated with neurodevelopmental disorders often also cause congenital anomalies of other organ systems; it may be that, in a subset of children with such variants, some of the cognitive delay is due to early-life physical health challenges (for example, hospital admission for congenital heart disease, vision or hearing impairments) which resolve as children recover and receive targeted interventions. If this is true, one might predict that the attenuation of RVB associations with cognition with age will be strongest in children from less deprived households, which could be tested in larger datasets. Another important difference between the rare and common variant-based scores we have considered is that, in any given individual, the RVB will primarily reflect the presence of qualifying variants in one or a small number of genes, whereas the PGIs considered are composed of the small effects of thousands of genes. It may be that, for at least a subset of these single genes impacted by the rare variant/s, there are biological compensatory mechanisms that can attenuate the deleterious impact across development.

There are several limitations of this study. First, our primary findings about changing genetic associations with time are based on measurements of IQ at only three timepoints in a single cohort (ALSPAC). There is a dearth of cohorts with longitudinal measures of IQ in childhood and adolescence with sufficient sample size and appropriate genetic data for us to attempt direct replication. Thus, we have relied on replication with school grades measured at two timepoints in ALSPAC, or, for some analyses, a single measure of cognitive performance from MCS and the UK Biobank. Broadly, the results from these replication analyses supported our primary findings in ALSPAC. Second, IQ tests are less reliable in early life^64^ and those used in ALSPAC may be less accurate at measuring the tails of the distribution^65^. If the test reliability differed between the tests used at different ages, this may have induced spurious interaction effects between the genetic scores and age. However, this does not seem a likely scenario given that we replicate trends in other cohorts and with other cognitive performance measures, and we see age interaction effects in opposite directions for PGIs versus RVBs for the same trait; if the only change across development were decreasing measurement error with age, we would expect the association for both genetic score constructs to increase with age. Third, the genetic scores we have used to capture the contributions of common and rare variants underestimate the total liability conferred by these variants. Thus, the questions addressed here should be revisited in future once much larger whole-genome sequenced cohorts become available. Another limitation is that our results may be affected by biased ascertainment, non-random missingness within the cohorts and attrition across time^33,66^. We have attempted to mitigate non-random missingness by imputation of missing IQ measures in ALSPAC and by Mendelian imputation of unmeasured parental genotypes for our trio-based PGI analyses. In general, our conclusions were unchanged when analysing IQ before versus after imputation, although there were some differences particularly in the findings from the quantile regressions (Supplementary Note 7; Fig. 3 versus Supplementary Fig. 6; Supplementary Fig. 5a versus 5b). To mitigate ascertainment bias and attrition, we incorporated weights in MCS to render the sample representative of the whole UK population. We also conducted several robustness analyses to assess the potential biases that attrition could have induced in our ALSPAC analyses, and found that non-random missingness, although present, is unlikely to have led to spurious inferences (Supplementary Note 8). One analysis that is likely to have been impacted by ascertainment bias is the estimate of the fraction of the rare variant effect on IQ that is due to de novo mutations, which we have probably overestimated (Supplementary Note 4 and Extended Data Fig. 5). Finally, because our study is based on observational data, our results should be viewed as identifying genetic associations, both between and within family, rather than as causal estimates of genetic effects.

Our findings highlight the value of considering genetic data in studies of social and environmental factors influencing cognitive outcomes. Future work should seek to confirm the relevance of our observations to the incomplete penetrance of rare damaging variants in NDCs, for which we are likely to need clinical cohorts with genetic and longitudinal phenotype data from both affected children and their parents. It should also seek to better understand why there are differential time-varying genetic associations in children depending on their level of cognitive ability and why these differ between PGIs and RVBs. Larger cohorts would allow us to construct more comparable scores for rare versus common variants, allowing for more direct comparison of their associations. They would also allow us to test whether particular neurobiological processes disproportionately contribute to the increasing heritability of cognitive ability, for example, using partitioned heritability approaches^67^. More broadly, our results suggest that heteroscedastic and time-varying genetic associations with human phenotypes deserve more exploration.

## Methods

This work complies with all relevant ethics regulations. The Cambridge South Research Ethics Committee (10/H0305/83), The Republic of Ireland Research Ethics Committee (GEN/284/12) and The East of England–Cambridge Central Research Ethics Committee (20/EE/0035) gave ethical approval for this work. ALSPAC was accessed under application number B3565, MCS under GDAC_2022_14 and the UK Biobank under 44165. This study was not pre-registered. No statistical methods were used to pre-determine sample sizes, but our sample sizes are similar to those reported in previous publications^24,52^.

### Cohorts

We report results from the Avon Longitudinal Study of Parents and Children^33,68^, Millennium Cohort Study^34^ and the UK Biobank cohorts. Participants in ALSPAC and MCS had their sex assigned at birth noted, whereas in the UK Biobank participants were asked their sex with at least the options of ‘female’ or ‘male’, and in all cohorts participants had their sex genetically inferred via genotype microarray data (male having XY and female having XX chromosomes). Individuals with discordant self-reported and genetically inferred sex were removed.

#### ALSPAC

In ALSPAC, pregnant women with expected deliveries between 1 April and 31 December 1991 were recruited in the greater Bristol area (formerly Avon county), resulting in an initial sample of 14,541 pregnancies enrolled in the study, of which 13,988 resulted in live births of children surviving to age 1. Data collected after the age of 7 were available for an additional 906 pregnancies from other phases of enrollment, which resulted in an additional 913 children that survived to age 1. At initial enrollment, 14,203 unique mothers were in the study, which increased to 14,833 unique mothers enrolled after the additional phases of enrollment (G0 mothers). The partners of G0 mothers (G0 partners) were also invited to participate in the study, of which 12,113 provided data at one point in the study and 3,807 are currently enrolled. From birth to early adulthood, mother and children were followed up with questionnaires and clinical and psychometric data collection across several timepoints. Biosamples used for genotyping and exome sequencing were obtained from most children and some of the mothers and fathers.

In the current study, data from 6,495 unrelated children with European inferred genetic ancestry (G1 children; described below) were used, along with genetic and/or survey data collected on 4,968 G0 mothers and 4,563 G0 partners. Note that the study website (http://www.bristol.ac.uk/alspac/researchers/our-data/) contains details of all data available through a fully searchable data dictionary and variable search tool. Ethics approval for the study was obtained from the ALSPAC Ethics and Law Committee and the Local Research Ethics Committees. Consent for biological samples was collected in accordance with the Human Tissue Act (2004). Informed consent for the use of data collected via questionnaires and clinics was obtained from participants following the recommendations of the ALSPAC Ethics and Law Committee at the time. At age 18, study children were sent ‘fair processing’ materials describing ALSPAC’s intended use of their health and administrative records, and were given clear means to consent or object via a written form. Data were not extracted for participants who objected, or who were not sent fair processing materials.

#### MCS

MCS recruited 18,552 pregnant mothers (2000–2002) using a sampling scheme to ensure a nationally representative sample across the UK, as previously described^34^. Mothers and children were followed longitudinally and genetic data were collected when the children were age 14, including from mothers and fathers where available. Ethics approval for the collection of saliva samples from these individuals as part of the sixth sweep was obtained from London-Central Research Ethics Committee. Genotype and newly generated exome data are available for ~8,000 and ~7,000 children, respectively (~13,000 and ~7,000 parents).

#### UK Biobank

The UK Biobank is a prospective cohort of over 500,000 individuals sampled throughout the UK between 2006 and 2010. Individuals have extensive phenotype data and genetic data including genotype array^69^ and whole-exome sequence data^70^.

### Cognitive performance measures across cohorts

In this study, we used various cognitive performance and school performance measures across the three cohorts.

In ALSPAC, children had IQ measured at ages 4 (Wechsler Preschool and Primary Scale of Intelligence), 8 (Wechsler Intelligence Scale for Children) and 16 (Wechsler Abbreviated Scale of Intelligence). Linked educational records are available, including national standardized exam scores in English, Math and Science administered at the end of Key Stage 2 (Year 6 at ~11 years old) and Key Stage 3 (Year 9 at ~14 years old). To create a composite measure of academic performance, we standardized each score and, at a given Key Stage examination point, computed a one-factor model score with the ‘factanal’ function in R using the Bartlett method for scoring, explaining 74% and 77% of the variance at Key Stages 2 and 3 exams, respectively. The factor loadings and factor variance explained for the three exams were nearly identical at Key Stages 2 and 3 (Supplementary Table 1), implying longitudinal invariance.

In MCS, children completed various cognitive performance tests at several ages. Those at later ages had a greatly reduced sample size, so we focused on those at ages 3 to 7. These included Bracken School Readiness at age 3, reading vocabulary at ages 3 and 5, pattern construction at ages 5 and 7, and word reading and progress in math at age 7. These were summarized into a single cognitive performance measure using a one-factor model score with the factanal function in R and Bartlett method for scoring, explaining 39% of the variance.

In the UK Biobank, individuals were asked 13 questions testing verbal–numerical reasoning in a limited time frame during the initial assessment visit. We used the sum of questions answered correctly as a measure of fluid intelligence (data field 20016.0), as has been used previously in genetic studies of cognitive ability^71^.

### Imputation of IQ values in ALSPAC

Imputation of missing IQ values was carried out using SoftImpute^37^ with rank.max = 4.5 and lambda = 4.5. Before imputation, all variables were standardized to have a mean of 0 and a variance of 1. To assess the imputation accuracy obtained using three potential sets of variables (described below), we set 100 random individuals with measured IQ values at a given age to ‘missing’, conducted phenotype imputation and calculated correlations between the true measured values and the imputed values, repeating this procedure 100 times.

We considered the following sets of variables:

1. Base set: sex (kz021), birth weight (kz030), maternal and paternal socioeconomic groups (b_seg_m and b_seg_p), and IQ values at ages 4 (cf813), 8 (f8ws112) and 16 (fh6280).
2. Expanded set: base set plus development score at age 2 (cf783), sociability score at age 3 (kg623a/c), additional verbal and performance IQ at age 4 (cf811, cf812), non-word repetition and multisyllabic word repetition at age 5 (cf470, cf480), communication score at age 6 (kq517), Skuse social cognition score at age 7 (kr554a/b), cognitive scores at age 8 including Sky Search, Diagnostic Analysis of Nonverbal Accuracy, non-word repetition, verbal and performance IQ, and children communication checklist score (f8at062, f8dv443, f8dv444, 8dv445, f8dv446, f8sl100, f8sl101, f8sl102, f8ws110, f8ws111 and ku506a).
3. Auxiliary set: expanded set excluding base set variables.

When calculating the imputation accuracy using the expanded or auxiliary sets, we removed verbal and performance IQ at each age, although in practice very few children had only one measured at a given age.

For the imputation of the final IQ values, we used the expanded set and required that each individual had non-missing values for at least one IQ test and at least 20% of the variables used for imputation. Final IQ values were standardized to a mean 0 and a standard deviation of 1 separately for measured and imputed values, and the measured and imputed values were then combined into a single variable.

### Genotype data preparation and imputation

#### ALSPAC

ALSPAC genotype data were generated and processed as described in ref. ^68^. We further removed individuals with high genotype missingness (>3%). We restricted analyses to autosomal SNPs with MAF > 0.5%, missingness rate <3%, and that had Hardy–Weinberg Equilibrium (HWE) test *P* > 1 × 10^−5^.

We used KING^72^ for relatedness inference and removed 152 samples who had first degree relationships but were reported as coming from different families, and another 16 samples who did match available exome sequencing data supposedly for the same individuals, resulting in 8,831 children, 9,302 mothers and 1,706 fathers. To identify a set of unrelated children, we iteratively identified the child with the greatest number of genetically inferred relatives (3rd degree or closer) and removed them, recalculated the number of inferred relatives per child, and repeated the first step until no children were inferred to have any relatives. This resulted in a total of 6,495 unrelated children with genotype array and whole-exome sequence data.

To identify individuals of genetically inferred European ancestry, we projected samples onto 1,000 Genomes Phase 3 individuals^73^ using the smartpca function in EIGENSOFT (v.7.2.1)^74^. We used linkage disequilibrium (LD)-pruned SNPs (pairwise *r*^2^ < 0.2 in batches of 50 SNPs with sliding windows of 5) with MAF > 5% and removed 24 regions with high or long-range LD, including the human leucocyte antigen region^75^. All ALSPAC samples projected onto European ancestry samples. We then performed principal component analysis (PCA) identically on the unrelated ALSPAC samples and projected all individuals onto the PCA space.

Before imputation, we removed palindromic SNPs, SNPs that were not in the imputation reference panel, and SNPs with mismatched alleles. We imputed the samples to the TOPMed r2 reference panel using the TOPMed imputation server^76–78^. We kept well-imputed common variants with Minimac4 *R*^2^ > 0.8 and MAF > 1%.

#### MCS

MCS genotyped data were generated and processed as described in ref. ^79^. A set of unrelated individuals with genetically inferred European ancestry was identified identically to the procedure used in ALSPAC above. Imputation was also conducted identically as in ALSPAC. We kept well-imputed common variants with Minimac4 *R*^2^ > 0.8 and MAF > 1%.

#### UK Biobank

UK Biobank genotype data were generated, processed and imputed by UKB as described in ref. ^69^. Sample quality control consisted of excluding individuals with >3% missingness, inconsistent sex, sex aneuploidy or withdrawn consent, and relatedness was similarly calculated using KING as previously described. As in the other cohorts, we kept autosomal SNPs with MAF > 1%, missingness rate <3% and that passed the HWE test (*P* > 1 × 10^−5^). To identify UKB individuals with genetically inferred European ancestry, we similarly projected samples onto the 1,000 Genomes Phase 3 individuals and assigned individuals to a genetic ancestry based on their Mahalanobis distance to the nearest continental ancestry group centroid using the top 6 PCs. Those more than 6 standard deviations from the centroid along any axis were removed. Unrelated individuals were identified using the iterative exclusion procedure as previously described, resulting in 387,531 unrelated individuals. PCs were recalculated in this subset of unrelated individuals using smartpca as previously described, and related individuals were projected onto this PCA space.

### Calculating polygenic scores

SNP weights for polygenic scores were estimated using LDpred2-auto^80^, which does not require a tuning dataset and automatically estimates the required hyperparameters from the discovery sample^81^. The LD reference panel was computed from unrelated individuals from the target dataset for a set of 1,444,196 HapMap3+^82^ variants^69^. GWAS summary statistics for EA^13^ excluding 23andMe or 23andMe only (used for PGI_EA_ UK Biobank samples), EA-Cog and EA-NonCog^41^ were matched with the list of overlapping SNPs.

Once the weights were generated, we scored individuals using the ‘–score’ function in PLINK v.1.9, which calculates the weighted sum of genotypes across a set of SNPs for each individual.

### Mendelian imputation of parental genotypes

When an individual had at least one parent with genotype data in the cohort, we imputed the expected parental genotype of the other parent. To do so, we used the ‘snipar’^17^ package across the three cohorts. We supplied relatedness inference using the KING output and used the default options throughout. We used the previous weights derived from each GWAS using LDpred2-auto to calculate the PGIs in the full trios using the ‘pgs.py’ script provided in the snipar package.

### GWAS of IQ and cognitive performance

We used the linear regression function ‘lm’ in R to conduct GWASs on the IQ measures pre- and post-imputation in ALSPAC and the cognitive performance measure in MCS on unrelated genetically inferred European ancestry individuals, controlling for 10 genetic PCs and sex. We removed variants with MAF < 1% or missingness >2%.

### Heritability and genetic correlations

We used both GREML-LDMS^38^ and LD score regression^83,84^ to estimate SNP heritabilities and genetic correlations from the summary statistics of the GWASs conducted on the IQ measures and external GWASs using the previously described LD reference panel on HapMap3 SNPs.

### Other exposures associated with IQ in ALSPAC and MCS

We considered the following exposures: maternal and paternal educational attainment (EA), weeks born preterm, and a composite variable representing maternal illness in ALSPAC only. For educational attainment, in ALSPAC we used the highest qualification reported by the parent and coded it as previously reported^13^, with a university degree equalling 20 years of education, A levels as 13, O levels/vocational as 10, and Certificate of Secondary Education/no degree as 5 (c645a and c666a for the mother and father, respectively); in MCS we used the equivalent self-reported derived variables in sweep 1 from each parent questionnaire (APLFTE00). Maternal illness was defined as a binary variable indicating whether or not a mother had at least one of the following conditions reported during the pregnancy in her obstetric clinical records: preeclampsia, anaemia (DELP_1060), any diabetes (pregnancy_diabetes), or genital herpes, gonorrhoea, syphilis, urinary tract infection, vaginal infection, or hepatitis B noted during delivery (DEL_P1050-1055). Weeks born preterm was coded as 40 minus the gestational age at birth (in weeks) assessed in a formal paediatric assessment (DEL_B4401). All variables were standardized to have a mean of zero and a standard deviation of 1.

### Exome-sequencing data preparation

Whole-exome sequencing data quality control (QC) for ALSPAC and MCS cohorts was carried out by the Human Genetics Informatics team at the Sanger Institute as described in ref. ^42^. Briefly, GATK v.4.2 was used to call short variants (single-nucleotide variants (SNVs) and insertions/deletions (indels)) in 11,994 samples from ALSPAC and 15,050 samples from MCS. Sample QC measures were employed to remove outliers on several metrics (for example, heterozygosity, variant counts) and likely sample mismatches. To identify low-quality variants (variant QC), a random forest was trained on pre-defined truth sets in each cohort individually. Random forest filtering was then applied in combination with genotype-level and missingness filters to balance precision, recall, true and false positive rates, and synonymous transmission ratios. Specifically, SNVs were filtered (genotypes set to missing) if they had an allele depth (DP) < 5, a heterozygous allele balance ratio (AB) < 0.2, or a genotype quality (GQ) < 20 (ALSPAC) or <15 (MCS); indels were filtered using these thresholds: DP < 10, AB < 0.3, GQ < 10 (ALSPAC) or GQ < 20 (MCS). The variants were excluded if they failed the random forest filtering or if the fraction of missing genotypes (missingness) exceeded 0.5. The final dataset included 8,436 children and 3,215 parents in ALSPAC, and 7,667 children and 6,925 parents in MCS. Calling and QC of de novo mutations is described in the Supplementary Methods.

In the UK Biobank, we performed quality control for whole-exome sequencing data on 469,836 participants within the UKB research analysis platform. First, we split and left-aligned multi-allelic variants in the population-level variant call format files into separate alleles using ‘bcftools norm’^85^. Next, we performed genotype-level filtering using bcftools to separately filter for SNVs and indels using a missingness-based approach. Specifically, SNV genotypes with a depth lower than 7 and genotype quality lower than 20, or indel genotypes with a depth lower than 10 and genotype quality lower than 20, were set to missing. We further tested for an expected alternate allele contribution of 50% for heterozygous SNVs using a binomial test, and SNV genotypes with a binomial test *P* ≤ 0.0001 were set to missing. Finally, we recalculated the proportion of individuals with a missing genotype for each variant and excluded all variants with a missingness value greater than 50%.

### Classification of deleterious rare variants

All variants were annotated using the MANE transcript from Ensembl^86^. In MCS and ALSPAC, rare pLoFs were defined as pLoFs annotated as high confidence by LOFTEE^31^ that had a combined annotation dependent depletion (CADD) score > 25 (ref. ^87^) (if SNVs), were not located in the last exon or intron, and had a gnom-AD^31^ V3 allele frequency of <3 × 10^−5^ (up to ~5 occurrences in gnomAD r3 genomes & ~10× in exomes), and an in-sample allele frequency of <0.1% among the unrelated set of children from that cohort^86^. Rare damaging missense variants were defined using identical allele frequency and CADD filters to pLoFs but were additionally required to have a missense badness, PolyPhen-2 and constraint (MPC) score^88^ ≥2. Rare synonymous variants were defined with identical allele frequency thresholds.

In UKB individuals with inferred European genetic ancestry, we defined rare variants as those with a within-sample allele frequency <0.001%. For pLoFs, we retained only those variants defined as high-confidence pLoFs by LOFTEE and had CADD > 25 as in MCS and ALSPAC. For missense variants, we defined the damaging missense variants by including variants with REVEL >0.5, AlphaMissense^89^ >0.56 and MPC >2.

### Calculating rare variant burden

We calculated RVB using the following formula:

1 $${\mathrm{RVB}}_{i,c}\,=\,\mathop{\sum }\limits_{g\in G}{I}_{g,c}(i){S}_{g}$$

where ${I}_{g,c}(i)$ is an indicator function for whether an individual *i* has a variant of consequence *c* in gene *g*, ${S}_{g}$ is the fitness cost for heterozygous carriers of a pLoF allele in gene *i* estimated in ref. ^44^, and *G* is the set of all autosomal genes.

For analyses to ascertain the relative contribution of de novo versus inherited variants (Supplementary Note 4), we also calculated a separate rare variant burden metric which we call ‘constrained variant count’:

2 $${\mathrm{constrained}\,\mathrm{pLoF}\,\mathrm{count}}_{i}=\mathop{\sum }\limits_{g\in \mathrm{constrained}\,\mathrm{genes}}{I}_{g,\mathrm{pLoF}}(i)$$

where ${I}_{g,\mathrm{pLoF}}(i)$ is an indicator function for whether an individual *i* has a pLoF in gene *g*, and ‘constrained genes’ is the set of genes identified as constrained in ref. ^44^. For Extended Data Fig. 4, we calculated each RVB in three mutually exclusive gene sets from Li et al.^90^, as well as in genes prioritized from various GWASs^6,91,92^.

### Sampling and non-response weights in MCS

Sampling weights were developed by MCS to adjust for the non-random sampling scheme devised for the study^93^. We used the full UK sampling weights. Non-response inverse probability weights were developed for each sample as done in ref. ^23^. Specifically, we fitted a logistic regression model predicting whether an MCS child had available genotype data, using the following variables collected at the first study sweep (where missingness was minimal, with over 96% of individuals having complete data): housing tenure, parental years of education, language spoken at home, study sampling stratum, single parent status and breastfeeding status. The study stratum variable comprised nine categories defined by country within the UK (England, Wales, Scotland, Northern Ireland), each stratified by area-level advantage or disadvantage, with an additional ethnic minority stratum in England. We fitted this model separately for two target samples: first, to predict membership in the set of unrelated individuals with genetically inferred European ancestry and genotype data (*N* = 5,884 of 6,036 children with complete covariate data); and second, to predict membership in the subset of these children who additionally had genotyped parents forming complete trios (*N* = 2,445 of 2,498 trio children with complete covariate data). In the trio model, an additional covariate indicating whether the child had any genotype data was included. These models achieved Nagelkerke *R*^2^ values of 0.51 and 0.42, respectively. For each genotyped individual, we extracted the predicted probability of being in the target sample and took its inverse as the non-response weight, such that individuals with lower predicted probabilities of inclusion who were nonetheless present in the sample received higher weights. Individuals whose weights exceeded three standard deviations above the mean were excluded as likely reflecting data errors driving implausibly low predicted probabilities; this removed 14 individuals from the full genotyped sample and 3 from the trio subset. Final combined weights were obtained by multiplying the MCS-provided sampling weights by our non-response weights, and these were incorporated into all relevant regression analyses using the weights argument in R.

### Associations between genetic measures and traits

Cross-sectional associations for a genetic score and IQ/cognitive performance measure were conducted using the lm function in R, restricting to unrelated samples with genetically inferred European ancestry as follows:

3 $${{\rm{IQ}}}_{i}\,\sim \,{G}_{i}\,+\,{\rm{PC}}{1}_{i}\,+\,\ldots \,+{\rm{PC}}{10}_{i}\,+\,{{\rm{sex}}}_{i}$$

where *G*_*i*_ is the genetic score (PGI or RVB) for child *i* and PC1–10_*i*_ are their genetic PCs.

Genetic scores, unless otherwise stated, were standardized to a mean of 0 and a standard deviation of 1. In trio-based analyses, the parental genetic values were similarly standardized and regression was conducted as follows:

4 $${{\rm{IQ}}}_{i}\sim {G}_{i}+{G}_{i,{\rm{m}}}+{G}_{i,{\rm{p}}}+{\rm{PC}}{1}_{i}+\ldots +{\rm{PC}}{10}_{i}+{{\rm{sex}}}_{i}$$

where *G*_*i*,m_ and G_*i*,p_ are the genetic scores for child *i*’s mother and father, respectively.

Mixed-effects linear models were conducted using the ‘lme4’ package in R^94^. For our primary models, in addition to Wald-test *P* values, we obtained heteroscedasticity-robust inference by performing a parametric bootstrap of the fixed-effect estimates (1,000 replicates) via bootMer (type = ‘parametric’) in lme4. Bootstrap standard errors and *P* values are reported in Supplementary Tables 4 and 6. The same covariates were used for the cross-sectional analysis with the addition of age, an age × genetic score interaction effect and a child-specific random intercept term as follows:

5 $${{\rm{IQ}}}_{i,t}\sim {{\rm{age}}}_{i,t}+{G}_{i}+{{\rm{age}}}_{i,t}\ast G{\rm{i}}+{\rm{PC}}{1}_{i}+\ldots +{\rm{PC}}{10}_{i}+{{\rm{sex}}}_{i}+{C}_{i}$$

where age_*i*,*t*_ and IQ_*i*,*t*_ are child *i*’s age and IQ at age *t*, and *C*_*i*_ is a child-specific random effect intercept. Age was coded as age-4 such that the genetic predictor intercept estimate was equivalent to the effect at the first age at which IQ data were collected. In the trio-based analyses, an age interaction effect was additionally modelled for the parental genetic values as follows:

6 $$\begin{array}{l}{\mathrm{IQ}}_{{\rm{i}},{\rm{t}}}\sim {\mathrm{age}}_{{\rm{i}},{\rm{t}}}+{{\rm{G}}}_{{\rm{i}}}+{\mathrm{age}}_{{\rm{i}},{\rm{t}}}\ast {{\rm{G}}}_{{\rm{i}}}+{{\rm{G}}}_{{\rm{i}},{\rm{m}}}+{\mathrm{age}}_{{\rm{i}},{\rm{t}}}\ast {{\rm{G}}}_{{\rm{i}},{\rm{m}}}\\ \,\,\,\,\,\,\,\,+{{\rm{G}}}_{{\rm{i}},{\rm{p}}}+{\mathrm{age}}_{{\rm{i}},{\rm{t}}}\ast {{\rm{G}}}_{{\rm{i}},{\rm{p}}}+\mathrm{PC}{1}_{{\rm{i}}}+\ldots +\mathrm{PC}{10}_{{\rm{i}}}+{\mathrm{sex}}_{{\rm{i}}}+{{\rm{C}}}_{{\rm{i}}}\end{array}$$

For cross-sectional and longitudinal quantile regression modelling, we used the R package ‘quantreg’^95^ and ‘rqpd’^96^, respectively, using the same models as above. Mixed-effects model standard errors were determined using bootstrapping with 500 bootstrap replications.

For the academic performance score, the cross-sectional models were performed in the same way as described above, with the addition of age at testing (in weeks) as a covariate (ks2age_w and ks3age_w). As there were only two timepoints, a slightly different longitudinal model was used for the linear modelling (Extended Data Fig. 2) as follows:

7 $$({{\rm{APS}}}_{9,i}-{{\rm{APS}}}_{6,i})\sim {G}_{i}+{{\rm{age}}}_{6,i}+{{\rm{age}}}_{9,i}+{\rm{PC}}{1}_{i}+\ldots +{\rm{PC}}{10}_{i}+se{x}_{i}$$

where APS_6/9,*i*_ and age_6/9,*i*_ are the academic performance scores and ages of individual *i* at Years 6 and 9, respectively. The coefficient estimated for G_*i*_ in this regression is equivalent to *G*’s effect at Year 9 minus that at Year 6 within an individual, that is, the age interaction effect. To compare the effects for the quantile regression effect size estimates at Years 6 and 9, we conducted a *Z*-test between the effect sizes (Extended Data Fig. 7).

For the linear models, we assumed normally distributed residuals and additionally, for mixed-effects linear models, random effects. We assessed the potential impact of heteroscedasticity on our inference by re-estimating key models with bootstrap-derived standard errors (Supplementary Tables 4 and 6), which produced virtually identical results. Quantile regression does not assume normally distributed errors and is robust to heteroscedasticity.

All statistical tests were two-tailed, except for the *F*-test of variance differences, which was one-tailed by construction. Assumptions for statistical tests were not explicitly tested. This study was not pre-registered.

### Marginal and conditional associations between genetic and other factors and IQ

To assess the associations of genetic and other factors with IQ (Fig. 4), we considered both marginal and conditional models. For marginal models, we conducted the following regressions:

8 $${{\rm{IQ}}}_{i,t}\sim {{\rm{age}}}_{i,t}+{E}_{i}+{{\rm{age}}}_{i,t}\ast {E}_{i}+{\rm{PC}}{1}_{i}+\ldots +{\rm{PC}}{10}_{i}+{{\rm{sex}}}_{i}+{C}_{i}$$

where *E*_*i*_ is the given measure of interest. In Fig. 4, the coefficient on the *E*_*i*_ term (that is, the main effect) is shown in the top panel, and the coefficient on the age_*i*,*t*_ × *E*_*i*_ term (that is, the interaction effect) in the bottom panel.

As maternal and paternal EA are highly correlated, we modified the model for the effects of parental EA as follows:

9 $$\begin{array}{l}{\mathrm{IQ}}_{{\rm{i}},{\rm{t}}}\sim {\mathrm{age}}_{{\rm{i}},{\rm{t}}}+{\mathrm{EA}}_{{\rm{i}},{\rm{m}}}+{\mathrm{EA}}_{{\rm{i}},{\rm{p}}}+{\mathrm{age}}_{{\rm{i}},{\rm{t}}}\ast {\mathrm{EA}}_{{\rm{i}},{\rm{m}}}\\ \,\,\,\,\,\,\,\,+{\mathrm{age}}_{{\rm{i}},{\rm{t}}}\ast {\mathrm{EA}}_{{\rm{i}},{\rm{p}}}+\mathrm{PC}{1}_{{\rm{i}}}+\ldots +\mathrm{PC}{10}_{{\rm{i}}}+{\mathrm{sex}}_{{\rm{i}}}+{{\rm{C}}}_{{\rm{i}}}\end{array}$$

where EA_*i*,m_ and EA_*i*,p_ are the maternal and paternal EA, respectively. Similarly, we fit the PGI_EA_ values for the child, mother and father jointly in a trio model.

To calculate the variance explained by a given variable, we took the square of the standardized effect size of that variable’s main effect. To calculate the total variance explained by two uncorrelated variables (for example, RVB_pLoF_ and RVB_Missense_ that have a correlation of −0.01, *P* = 0.16), we summed the squares of their standardized effect sizes. To test for differences in effect size between the combination of RVB_Missense_ and RVB_pLoF_ versus other factors, we used the square root of the previous variance explained estimate as the effect size estimate and determined the standard error for that effect size by summing the square of the standard error for each effect estimate and then taking the square root of the new estimate. We were then able to use a *Z*-test to compare the effect size estimates as done previously.

For the full joint models, we included all genetic and other measures in a single model, with a main and age interaction effect for each measure. Since only a subset of probands had information on gestational age, we fitted the full joint model both with and without the ‘weeks born preterm’ variable.

### Reporting summary

Further information on research design is available in the Nature Portfolio Reporting Summary linked to this article.

## Supplementary information

Supplementary Information Supplementary Methods, Table legends, Figs. 1–13, Notes 1–8, Discussion and Frequently Asked Questions.

Reporting Summary

Peer Review File

Supplementary Table Supplementary Tables 1–15.

## Figures and Tables

**Extended Data Fig. 8:**
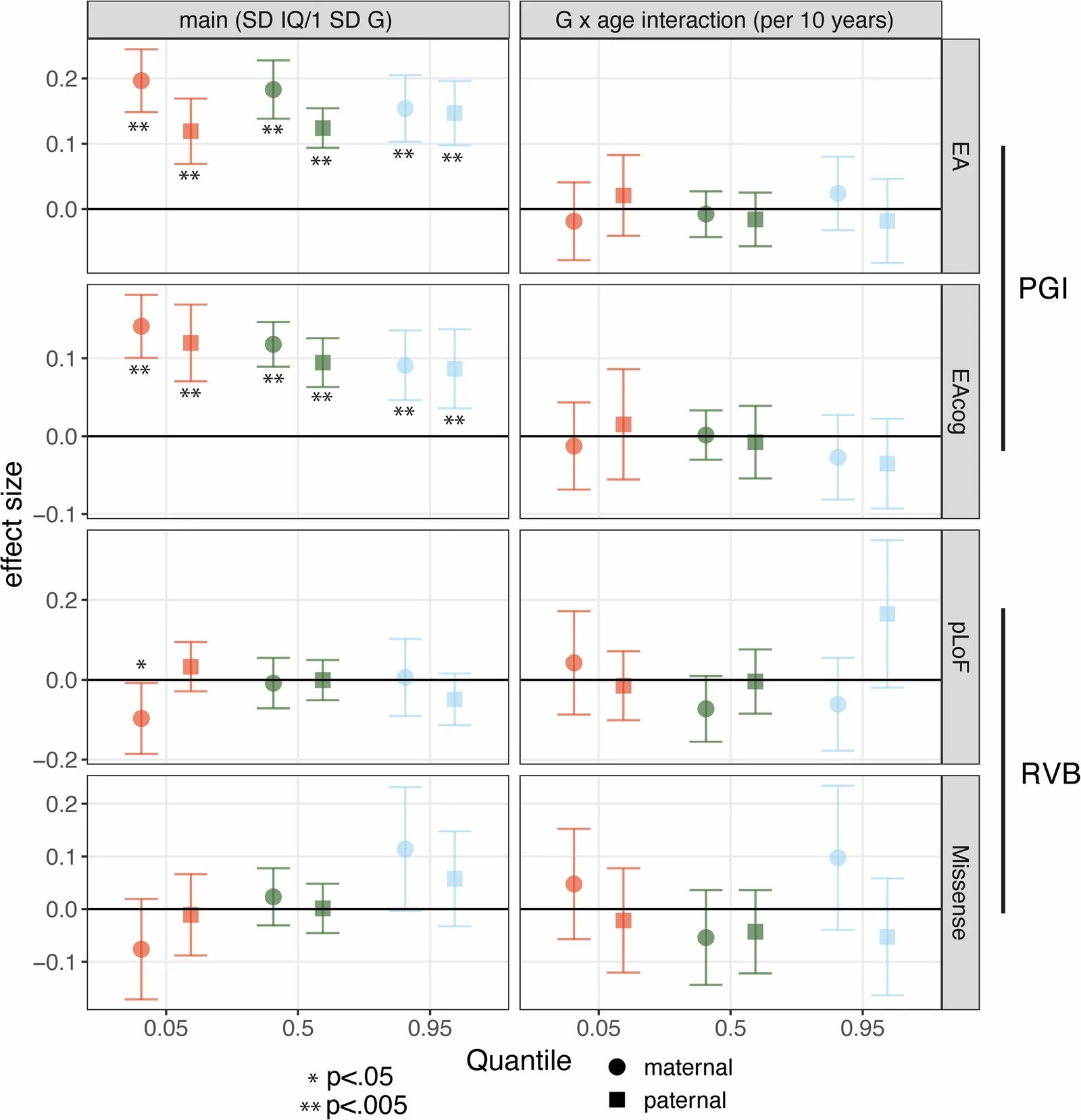
Influence of parental common and rare variants on different quantiles of the IQ distribution post-imputation. Standardized effect sizes and 95% confidence intervals for quantile regression of the 5th, 50th, and 95th percentiles estimated from mixed-effects modeling with post-imputation IQ at ages 4, 8, and 16 for parental PGI_EA_, PGI_Cog_, RVB_pLoF_ and RVB_Missense_ estimated in a trio model (n = 6,495). Effect sizes and p values were estimated using mixed-effects quantile regression with bootstrapped standard errors (500 replicates; two-sided tests). Point estimates, 95% confidence intervals, and p values are provided in Supplementary Table 8.

## Data Availability

Researchers can apply to access data from ALSPAC (https://www.bristol.ac.uk/alspac/researchers/access/), MCS (https://cls.ucl.ac.uk/dataaccess-training/data-access/) and the UK Biobank (https://www.ukbiobank.ac.uk/enable-your-research/apply-for-access).
