# Supplementary material for: Common and rare genetic variant associations with cognitive performance across development in British birth cohorts
Source: Nat Hum Behav. 2026 Jul 10;10(9):1841–54. doi: 10.1038/s41562-026-02491-8 (PMC13590407; doi:10.1038/s41562-026-02491-8)
Supplement: Supplementary file 1 [file 41562_2026_2491_MOESM1_ESM.pdf]

nature human behaviour

Supplementary information

https://doi.org/10.1038/s41562-026-02491-8

Common and rare genetic variant associations with cognitive performance across development in British birth cohorts

In the format provided by the authors and unedited

Supplementary Methods

De novo variant calling in ALSPAC and MCS

We carried out DNM calling in 1,326 trios in ALSPAC and 3,106 trios in MCS. Starting with the unfiltered callset obtained by GATK (described in detail in our recent data note[1]), a set of candidate *de novo* mutations (DNMs) was generated using `bcftools +trio-dnm2 --use-NAIVE` (http://samtools.github.io/bcftools/trio-dnm.pdf). In this mode, the program detects variants that violate the Mendelian patterns of inheritance simply by comparing parental and proband genotypes. However, a callset produced this way is dominated by false positives due to sequencing, mapping or alignment artifacts. In order to filter such artifacts, the following pipeline was used (Supplementary Figure 11A):

1. Per-trio genotyping. We generated the following annotations at each candidate site using `bcftools mpileup -pa AD,QS,SP,SCR,FMT/NMBZ`:
    - AD, allelic depth, the number of reference and alternate reads observed in the three samples
    - QS, phred-score allele quality sum, an auxiliary annotation used by `bcftools +trio-dnm2`
    - SP, phred-scaled strand bias P-value
    - SCR, number of soft-clipped reads
    - NMBZ, Mann-Whitney U-z test of number of mismatches within supporting reads
2. Parental genotyping. We generated profiles of parental variant allele fraction (VAF) to identify problematic regions, as described in the section on "Parental VAF Profiles" below.
3. Obtaining posterior probabilities. We ran `bcftools +trio-dnm2 --strictly-novel --ppl` and `bcftools +trio-dnm2 --strictly-novel` to obtain posterior *de novo* probabilities using the DeNovoGear model[2] implemented in the trio-dnm2 plugin (http://samtools.github.io/bcftools/trio-dnm.pdf).
4. Random forest. We used random forest to score the candidate variants based on the following covariates:
    - DNM, the trio-dnm2 score
    - DNG, the DeNovoGear score
    - VCF_QUAL, variant quality as presented in the VCF QUAL column
    - MaxParentalVAF, maximum variant allele frequency (VAF) observed across parents at this site
    - NMBZ, Mann-Whitney U-z test of number of mismatches in variant reads
    - SCR, number of soft-clipped reads
    - SCBZ, Mann-Whitney U-z test of number of soft-clips in variant reads
    - MQBZ, Mann-Whitney U-z test of mapping quality of variant reads

    The random forest classifier was trained on a subset of calls manually inspected in IGV (Integrative Genomics Viewer)[3], separately for SNVs, deletions and duplications, and for autosomal and sex chromosomes in male and female samples. It was run with 100 trees

in the forest and the relative contributions of the classification annotations are shown in Supplementary Figure 11B.

5. Iterative manual curation and retraining of the random forest. We randomly selected 100 variants and curated them manually to extend the truth set, then re-trained the random forest classifier. We repeated this and the previous step 3-4 times until the random forest quality score separates clearly real from clearly false positive DNMs well.
6. We applied the following final filters:
    - In both ALSPAC and MCS
        - exclude any variants with population allele frequency 1% or higher (gnomAD v2.1)
        - exclude variants with parental depth lower than 8 reads
        - filter variants observed across unrelated parents, assessed with the LNP_VAF metric (log-normal probability of matching parental VAF profiles; i.e. we required LNP_VAF > -1000, see the section on “Parental VAF Profiles” below)
    - In ALSPAC
        - require random forest score bigger than 0.92
        - restrict C>A and G>T calls that passed stringent QC applied to the whole-exome data, due to a known sequencing artifact in the dataset[1]
    - In MCS
        - require random forest score bigger than 0.6
        - require VAF >= 25%

Note that different filters were applied in the two cohorts since they were differentially affected by a sequencing artifact described in the data note[1].

Parental VAF Profiles

The LNP_VAF annotation (log-normal probability of matching parental variant allele fraction profiles) is intended to filter sites in difficult regions by comparing the VAF profile in unrelated parents with the expected distribution. It is assumed that alternate reads in parents unrelated to the index proband represent sequencing errors. The method identifies a set of high-confidence *de novo* variants (i.e. sites with DNM score equal to 0) with sufficient coverage in all three trio samples (>=15x), proband VAF > 30%, and no alternate reads in the index proband's parents. For each such site, a VAF distribution is collected across all parents in the dataset who are unrelated to the proband of interest, and the mean μ and variance σ are determined across all sites for each bin of the distribution. The log-normal score is then calculated as follows:

LNP_VAF = $-\sum_{i=1}^{N}(x_i - \mu_i)^2/\sigma_i^2$, where N=50 is the number of bins of the VAF distribution.

It was then used in the filtering described above.

DNM callset summary and evaluation

The expected numbers of DNMs in different consequence classes were estimated per-gene for MANE transcripts from a sequence context-based mutational model [4]. The cumulative rates of expected DNMs per parental genome were as follows:

- synonymous: 0.160734,
- missense: 0.364192,
- loss-of-function, including stop gained, stop lost, start lost, splice acceptor, and splice donor consequence predictions: 0.0277504,
- stop gain: 0.0181173,
- frameshift:1.2*expected number of stop gain

In calculating the total expected number of DNMs in the dataset, the proportion of male vs female samples was taken into account as follows: 2*nSamples*dnm_rate for autosomal chromosomes and (nMales+2*nFemales)*dnm_rate for chrX. Pseudoautosomal regions were not considered in the calculation.

The mutation spectrum and distribution of parental VAF per dataset are shown in Supplementary Figure 12, and the comparison of observed to expected DNMs in each consequence class in Supplementary Figure 13. The number of DNMs in any one class or dataset did not differ significantly from the expected number, with the exception of missense mutations of which we observed a slight excess in MCS.

Gene set associations and enrichment

To derive gene set-specific associations (Extended Data Figure 4, Supplementary Figure 8, Supplementary Figure 9), we conducted mixed-effect linear modeling identically as above, restricting the RVB_{pLoF} calculation to genes only present in a given gene set. To make the effect sizes comparable between gene sets of varying sizes, we divided the effect sizes by the number of genes in the gene set.

To derive gene set-specific enrichment for a given gene set *g* of size *n*, we randomly sampled *n* genes not in *g* to generate a matched gene set *h*. To ensure that the distribution of heterozygous PTV selection coefficients in *h* is similar to *g*'s, we first calculated the 5-quantiles of the selection coefficient distribution of *g* and sampled *n*/5 genes from each quantile, rounded to the nearest upper whole integer. We then calculated a *h* specific RVB_{pLoF} and repeated the analyses above, repeating this procedure 100 times. We then combined the estimates of the 100 gene sets using Rubin's rule. Enrichment was calculated as the ratio of the RVB_{pLoF} effect sizes for *g* and the pooled effect sizes for *h*. Significance was determined using a Wald test.

Sensitivity analyses using simulated IQ measures

We carried out three Monte Carlo simulation scenarios to evaluate the impact of nonrandom missingness and age-dependent genetic effects on the estimated associations between IQ and two predictors, PGI_{EA} and RVB_{pLoF}. All simulations used the real missing data patterns observed on IQ at ages 4, 8 and 16 in ALSPAC.

1. Constant effects across age (constant effects model)

For each subject, we standardized PGI_{EA} and RVB_{pLoF} to mean=0 and SD=1. At each age, simulated IQ = 0.30xPGI + (–0.10)xRVB + ε, where $\varepsilon \sim N(0, \sigma^2)$ and σ^2 was chosen so that Var(IQ)=1. The effect sizes were chosen to approximate those observed empirically (Figure 1 and 2). The observed missingness mask was then applied, and estimated the mixed-effects regressions as done previously:

$$IQ_{i,t} \sim age_t + PGI_{EA,i} + RVB_{pLoF,i} + age_{i,t}*PGI_{EA,i} + age_{i,t}*RVB_{pLoF,i} + PC1_i+..+PC10_i+sex_i+ C_i$$

2. Increasing PGI effect with age (Positive PGI_{EA} x age interaction model)

This was the same model as above, except that the PGI coefficient grew linearly by 0.01 per year after age 4:
IQ = (0.30 + 0.01x[age - 4])xPGI + (-0.10)xRVB + ε.

3. Age-varying PGI plus heterogenous RVB effect (Positive PGI_{EA} x age interaction + RVB_{pLoF} heterogenous effect)

Building on scenario 2, we first computed a subject-level "baseline age 4 IQ" = 0.30xPGI + v where ($v \sim N(0, \sigma^2)$), then identified the bottom 50% of that distribution. Then, only those in the lower half received the RVB effect (–0.20xRVB); all others had no RVB contribution:

IQ = (0.30 + 0.01x[age - 4])xPGI + (-0.20)xRVB + v   if baseline age 4 IQ below 50th percentile
IQ = (0.30 + 0.01x[age - 4])xPGI + v   otherwise

The PGI effect continued to increase by 0.01 per year. This was intended to simulate an extreme case scenario, where the effects of RVB were only present in the lowest half of the baseline scores, though we note we empirically observed a much more subtle gradation of associations.

We repeated each scenario 1,000 times, refitting the mixed model to each simulated dataset to obtain distributions of the PGI_{EA} × age and RVB_{pLoF} × age slopes under each data-generating process.

Supplementary Tables

Supplementary Table 1

Key stage factor loadings and proportion of variance explained by one factor model on academic performance in ALSPAC.

Supplementary Table 2

Heritabilities and genetic correlations of the IQ measures in ALSPAC pre- and post-imputation, estimated using GREML-LDMS[5], with standard errors 95% CI, and p values shown (Wald test). Genetic correlations were estimated between post-imputation IQ measures or between pre-imputation IQ measures. GREML-LDMS estimates of heritability at age 4 pre-imputation are missing from this table as they did not converge. Estimates of heritabilities and genetic correlation between IQ at age 8 and 16 pre-imputation were calculated in unrelated individuals that had both measures.

Supplementary Table 3

Genetic correlations of the post-imputation IQ measures in ALSPAC and MCS cognitive performance PC1 with external GWASs using LDSC[6], with standard errors 95% CI, and p values shown (Wald test).

Supplementary Table 4

PGI associations for cross-sectional and mixed-effects models on IQ in ALSPAC. First column indicates the PGI being assessed in the regression, second column indicates whether the effect is the proband's population or direct effect estimate or the paternal/maternal effect estimate from a trio model, columns three through five indicate the effect size, standard error, and the p value, respectively, the sixth column indicates whether it was a cross-sectional or mixed-effects model, the seventh column indicates for a cross-sectional model the age at which the IQ test was administered or for a mixed-effects model whether it is an estimate of the main or age interaction effect, the eighth column indicates whether the regression was conducted before or after imputation of IQ measures, and the ninth column indicates whether the regression was conducted in the full cohort or the trio subset.

Supplementary Table 5

PGIs associations with academic performance in ALSPAC. First column indicates the PGI being assessed in the regression, second column indicates whether the effect is the proband's population or direct effect estimate or the paternal/maternal effect estimate from a trio model, columns three through five indicate the effect size, standard error, and the p value, respectively, the sixth column indicates the year at which the exams were administered or whether it is the age interaction estimated by taking the difference the scaled academic performance measures.

Supplementary Table 6

RVB associations for cross-sectional and mixed-effects models on IQ and cognitive performance in ALSPAC and MCS, respectively. First column indicates the RVB being assessed in the regression, second column indicates whether the effect is the proband's population or direct effect estimate or the paternal/maternal effect estimate from a trio model, columns three through seven indicate the effect size, standard error, and the p value, and 95% confidence intervals, respectively, the eighth column indicates whether it was a cross-sectional or mixed-effects model, the ninth column indicates for a cross-sectional model the age at which the IQ test was administered or for a mixed-effects model whether it is an estimate of the main or age interaction effect, the tenth column indicates whether the regression was conducted before or after imputation of IQ measures (not applicable in MCS), and the eleventh column indicates whether the regression was conducted in the full cohort or the trio subset. The twelfth column indicates the cohort.

Supplementary Table 7

RVB associations with academic performance in ALSPAC. First column indicates the RVB being assessed in the regression, second column indicates whether the effect is the proband's population or direct effect, columns three through five indicate the effect size, standard error, and the p value, respectively, the sixth column indicates the year at which the exams were administered or whether it is the age interaction estimated by taking the difference the scaled academic performance measures.

Supplementary Table 8

Quantile regression cross-sectional and mixed-effects model results for PGI and RVB associations with IQ in ALSPAC. First column indicates the PGI (EA, Cog, or Noncog) /RVB (pLoF or Missense) being assessed in the regression, second column indicates whether the effect is the proband's population or direct effect estimate or the paternal/maternal effect estimate from a trio model, columns three through five indicate the effect size, standard error, and the p value, respectively, the sixth column indicates whether it was a cross-sectional or mixed-effects model, the seventh column indicates for a cross-sectional model the age at which the IQ test was administered or for a mixed-effects model whether it is an estimate of the main or age interaction effect, the eighth column indicates the quantile being assessed, the ninth column indicates whether the regression was conducted before or after imputation of IQ measures, and the tenth column indicates whether the regression was conducted in the full cohort or the trio subset.

Supplementary Table 9

Quantile regression and difference in associations with PGI and RVB effects on academic performance and other cognitive performance measures in ALSPAC, MCS, and UK Biobank. First column indicates the PGI (EA, Cog, or NonCog) /RVB (pLoF or Missense) being assessed in the regression, second column indicates whether the effect is the proband's population or direct effect estimate or the paternal/maternal effect estimate from a trio model, columns three through five indicate the effect size, standard error, and the p value, respectively, the sixth column indicates the year at which the exams were administered or whether it is the age interaction estimated by taking the difference the scaled academic performance measures, the seventh column indicates the quantile being assessed.

Supplementary Table 10

Mean and standard deviation for PGIs and RVBs in ALSPAC participants with different IQ missingness patterns. First column indicates the PGI (EA, Cog, or NonCog) /RVB (pLoF or Missense). The following columns indicate mean and standard deviation of the scores, the IQ measure being considered and the imputation status of the group being considered, and the number of individuals in that group.

Supplementary Table 11

Simulation studies to assess biased estimation of age interaction effects pre-imputation in ALSPAC. The first column indicates the simulation study setting (Supplementary Methods). The second column indicates the variable for which the age interaction was tested. The third column indicates the mean and standard deviation of the age interaction estimate (per 10 years) minus the expected value (i.e., 0 if no interaction was simulated and minus 0.01 if an interaction of 0.01 per year was simulated). The fourth column indicates the percent of simulations in which the estimated interaction effect was both significant at 0.017 and in the direction of effect that was empirically observed (i.e., positive interaction for both PGI_{EA} and RVB_{pLoF}).

Supplementary Table 12

PGIs and RVB by age interaction population effects on IQ in ALSPAC estimated after omitting age 4 IQ measures. First column indicates the PGI/RVB interaction being assessed in the regression, the next three three indicate the effect size, standard error, and the p value, respectively, for the interaction effects estimated in ALSPAC using age 4, 8, and 16, the following three columns show identical values for estimates obtained in a model only including IQ measures from ages 8 and 16, and the final indicates the imputation status.

Supplementary Table 13

Sensitivity analysis in ALSPAC using a cognitive performance PGS constructed using Savage et al.[10] First column indicates the PGI being assessed in the regression, second column indicates whether the effect is the proband's population or direct effect estimate or the paternal/maternal effect estimate from a trio model, columns three through five indicate the effect size, standard error, and the p value, respectively, the sixth column indicates whether it was a cross-sectional, mixed-effects, or quantile regression model, the seventh column indicates for a cross-sectional model the age at which the IQ test was administered or for a mixed-effects model whether it is an estimate of the main or age interaction effect, the eighth column indicates whether the regression was conducted before or after imputation of IQ measures, the ninth column indicates whether the regression was conducted in the full cohort or the trio subset, the tenth and eleventh columns indicate the bootstrapped standard error and p value where applicable, the twelfth column indicates the quantile being assessed for quantile regressions, and the thirteenth column indicates the sample size.

Supplementary Table 14

Gene set RVB associations with cognitive performance measures in ALSPAC and MCS. First column indicates the RVB being assessed in the regression, second column indicates whether the effect is the main effect, age interaction, or enrichment, the third column indicates the gene set being analysed, columns four through eight indicate the effect size, standard error, 95% confidence intervals, and the p value, the ninth column indicates the imputation status and the tenth column indicates the cohort.

Supplementary Table 15

Associations between genetic and other factors and cognitive performance in ALSPAC and MCS. First column indicates whether the estimate is for the main effect or the measure-by-age interaction effect, second column indicates the variable being assessed (e.g., maternal EA, paternal EA, weeks born preterm, child/maternal/paternal PGI_EA, child RVB_pLoF, child RVB_Missense), third column indicates whether the estimate is from a marginal model (in which only the indicated variable was included alongside standard covariates) or a conditional model (in which all variables were included jointly), columns four through six indicate the effect size, standard error, and p value, respectively, columns seven and eight indicate the lower and upper bounds of the 95% confidence interval, and the ninth column indicates the cohort (ALSPAC or MCS).

Supplementary Figure Legends

Supplementary Figure 1

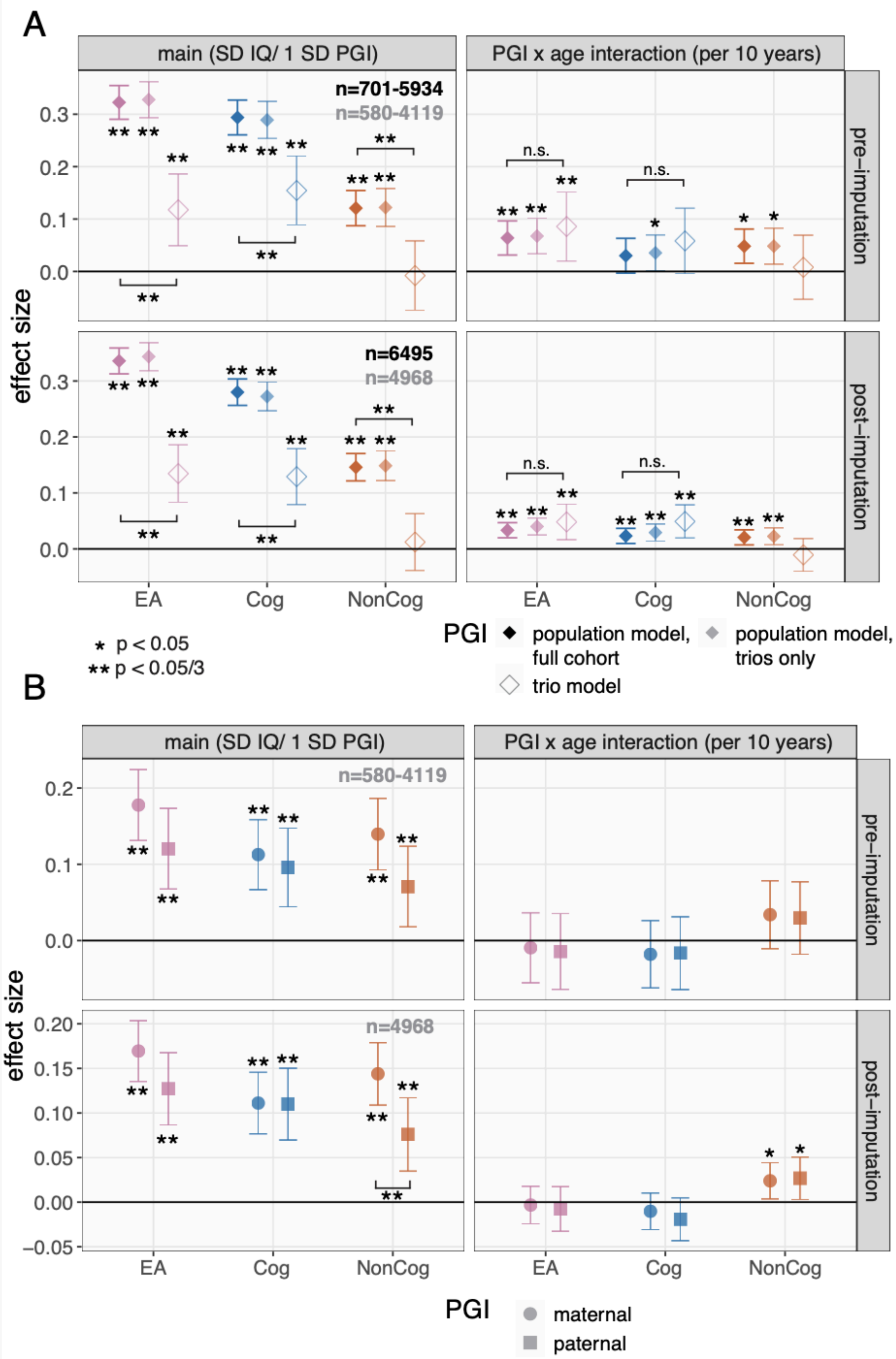

Association between PGIs and IQ across ages pre- and post-imputation. Standardized effect sizes and 95% confidence intervals estimated for the main effects and PGI-by-age-interaction effects for each PGI, either pre- (top) and post-imputation (bottom) A) Results for the children's PGIs not controlling (i.e. 'population model') and controlling for parental PGIs (i.e. trio model). Population effect sizes are shown

estimated in the full sample (opaque) and in the subset of children with parental PGIs (translucent), with the corresponding sample sizes shown in black and gray text respectively. B) Results for the parental PGIs from the trio model. SD: standard deviation; PGI: polygenic index; EA: educational attainment; Cog: cognitive component of EA from [7]; NonCog: non-cognitive component of EA from [7]. The square brackets indicate significant comparisons highlighted in the text (z tests).

Supplementary Figure 2

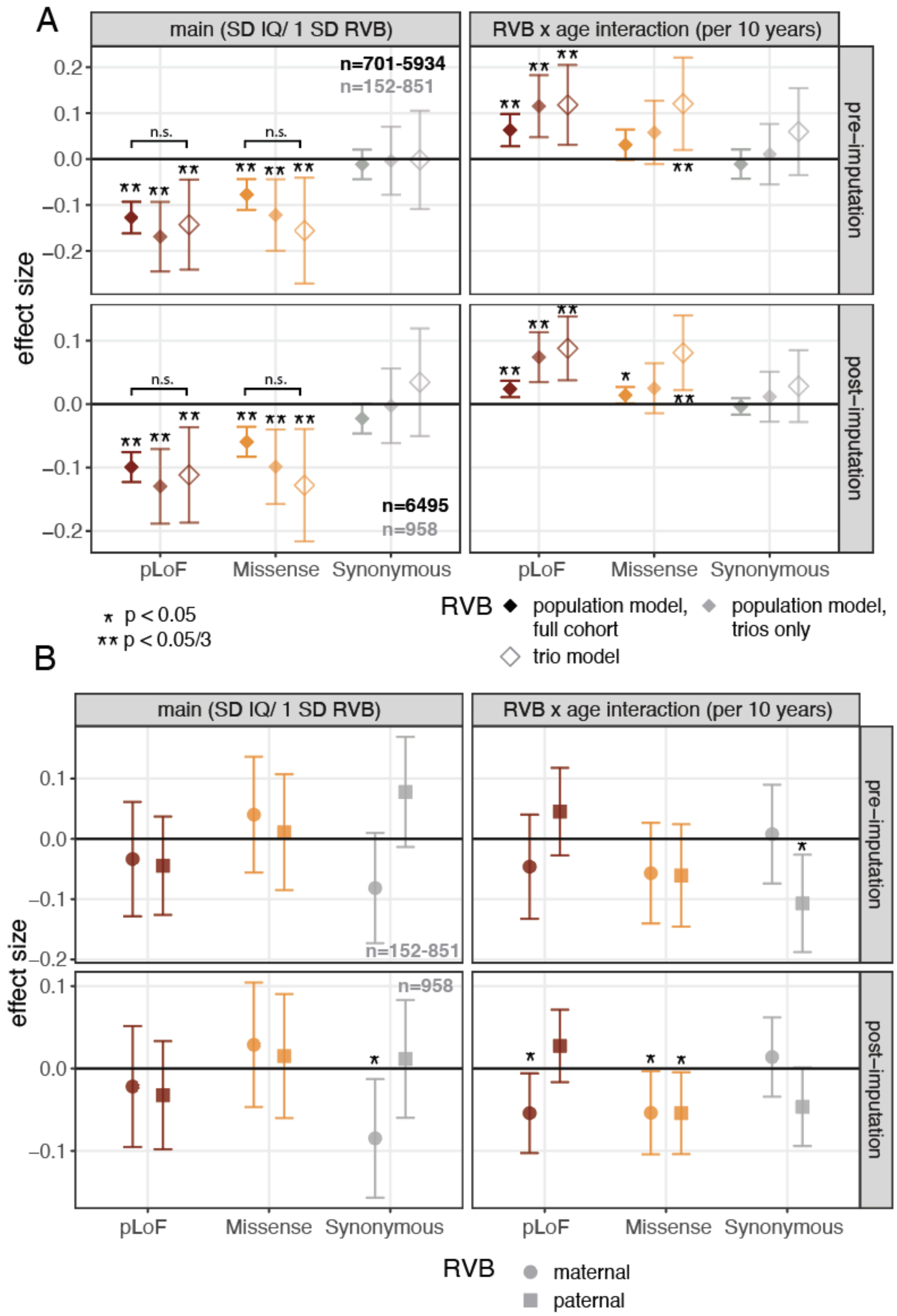

Association between rare variant burden (RVB) and IQ across ages pre- and post-imputation. Standardized effect sizes and 95% confidence intervals estimated for the main effects and RVB-by-age-interaction effects for RVB calculated with three different consequence classes (pLoF, missense or synonymous), either pre- (top) and post-imputation (bottom) . A) Results for the children's RVBs not controlling (i.e. 'population model') and controlling (right) for parental RVBs (i.e. trio model). Population effect sizes are shown estimated in the full sample (opaque) and in the subset of children with

parental RVBs (translucent), with the corresponding sample sizes shown in black and gray text respectively. B) Results for the parental RVBs from the trio model. The square brackets indicate comparisons highlighted in the text (z tests).

Supplementary Figure 3

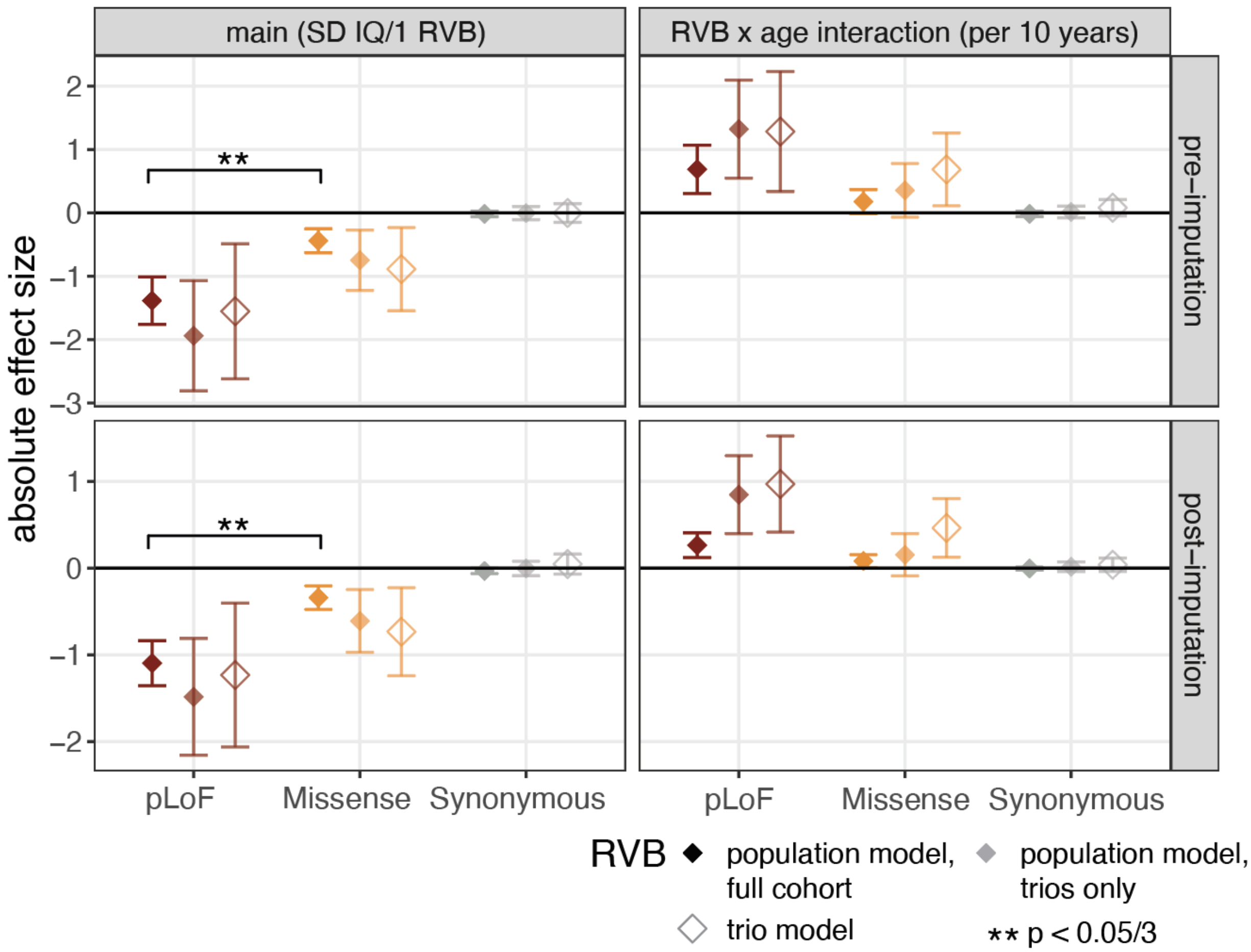

Association between unstandardized rare variant burden (RVB) and IQ across ages in ALSPAC, estimated with a mixed-effects linear model. (i.e. as for Figure 2 but with unstandardized rather than standardized RVB scores.) Absolute effect sizes and 95% confidence intervals estimated for the main effects and RVB-by-age-interactions for RVB calculated with three different consequence classes (pLoF, missense or synonymous), either pre- (top) and post-imputation (bottom). Results for the children's RVBs not controlling (i.e. 'population model') and controlling (right) for parental RVBs (i.e. trio model). Population effect sizes are shown estimated in the full sample (opaque) and in the subset of children with parental RVBs (translucent). The square brackets indicate significant comparisons highlighted in the text (z tests).

Supplementary Figure 4

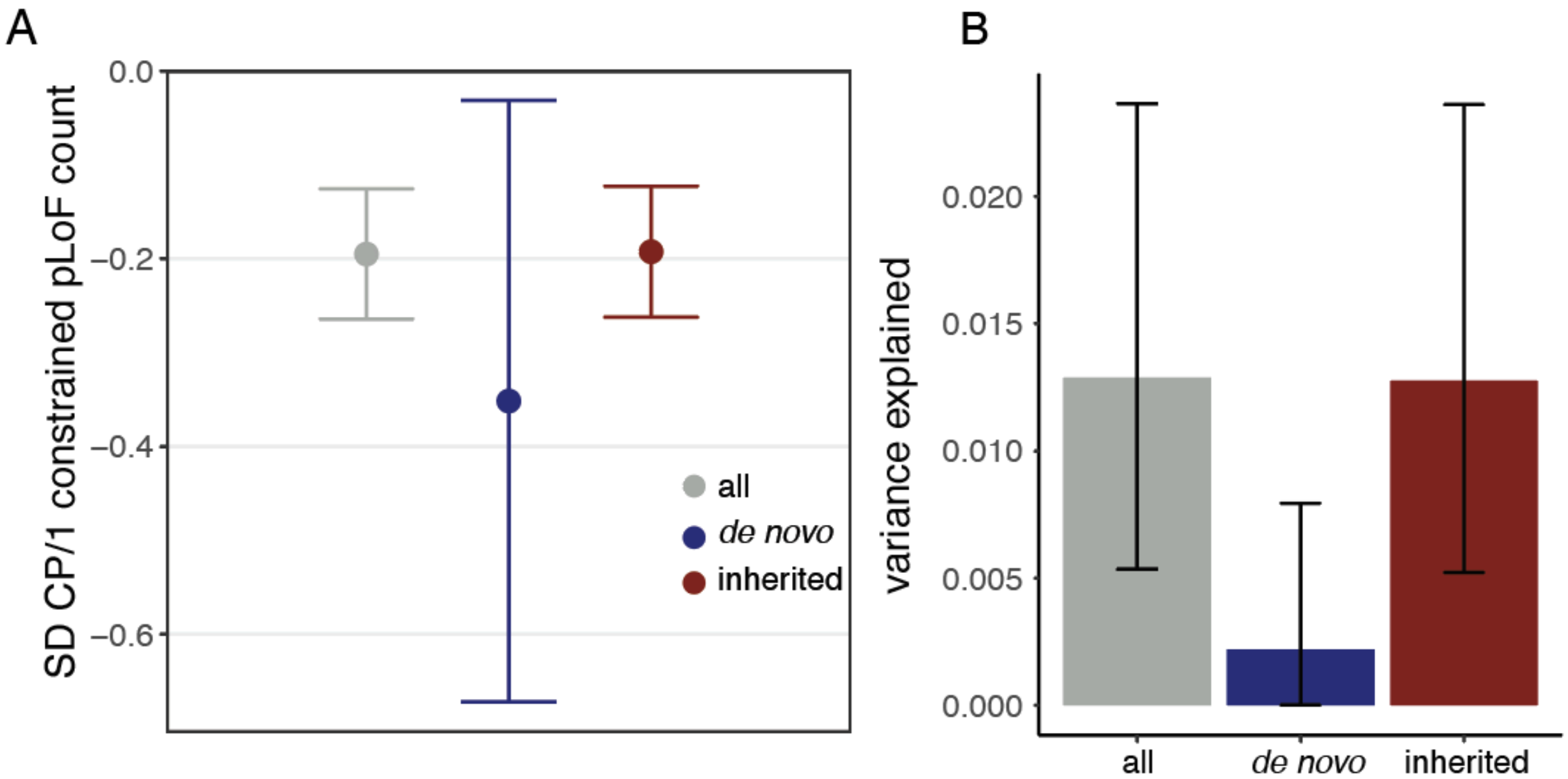

Association between inherited and *de novo* pLoF variant counts in constrained genes and composite cognitive performance scores in MCS. A) Standardized effect sizes for the main and RVB-by-age interaction effect from a longitudinal mixed-effects model of constrained pLoF counts on standardized cognitive performance scores when considering all variants or *de novo* mutations and inherited variants separately. B) Variance explained by constrained pLoF counts when considering all variants or *de novo* and inherited separately.

Supplementary Figure 5

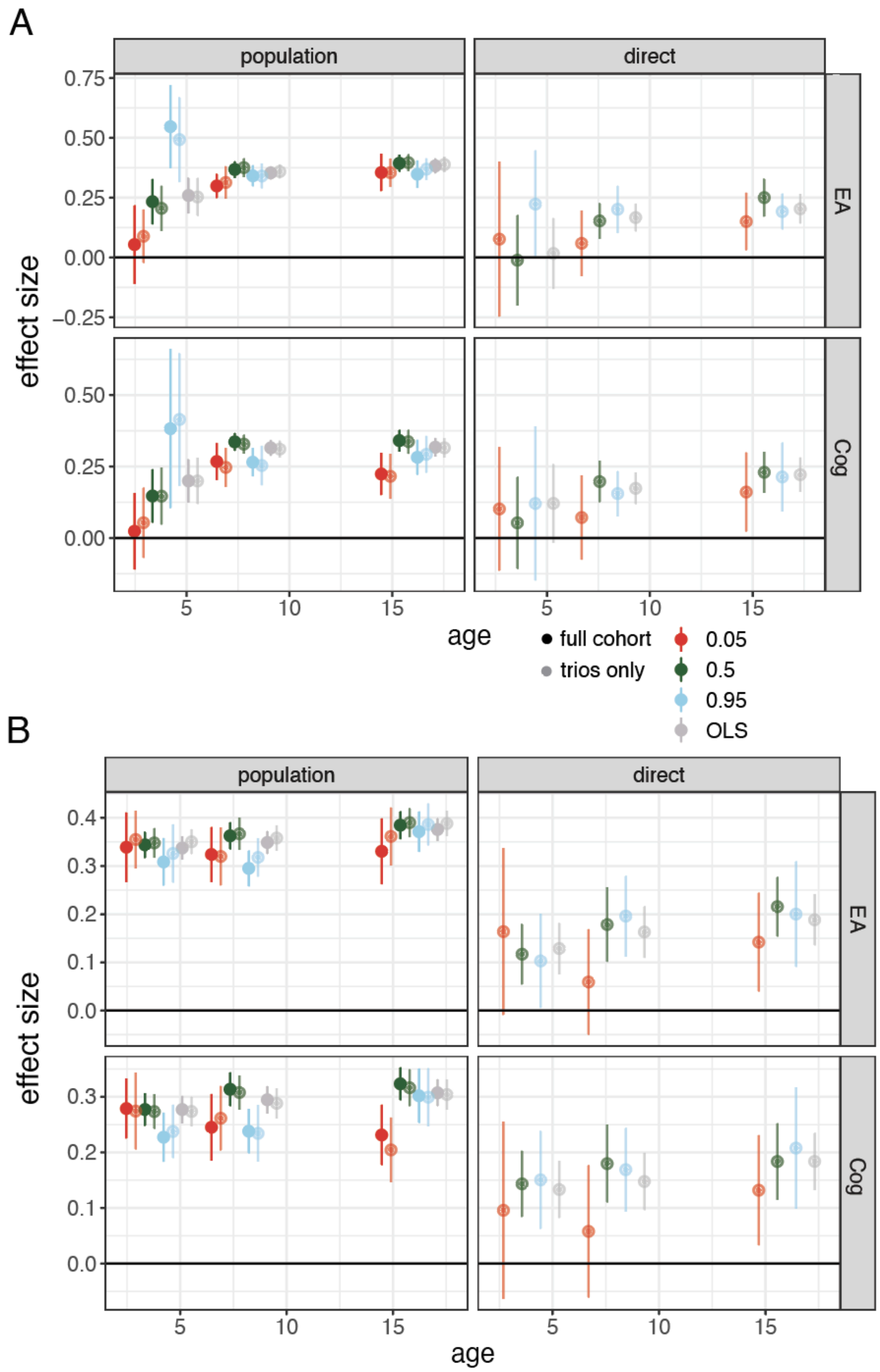

Association of PGIs with different quantiles of the IQ distribution in ALSPAC estimated by applying quantile regression in cross-sectional analyses. A) Results using pre-imputation IQ measures. Standardized effect sizes and 95% confidence intervals for quantile regression of the 5th (red), 50th (green), and 95th (blue) percentiles and ordinary least squares (OLS) linear regression (gray) estimated from cross-sectional associations with pre-imputation IQ at ages 4, 8, and 16 for PGI_{EA} and PGI_{Cog} before (left) and after (right) controlling for parental genetic measures. B) Same as (A) using post-imputation IQ measures.

Supplementary Figure 6

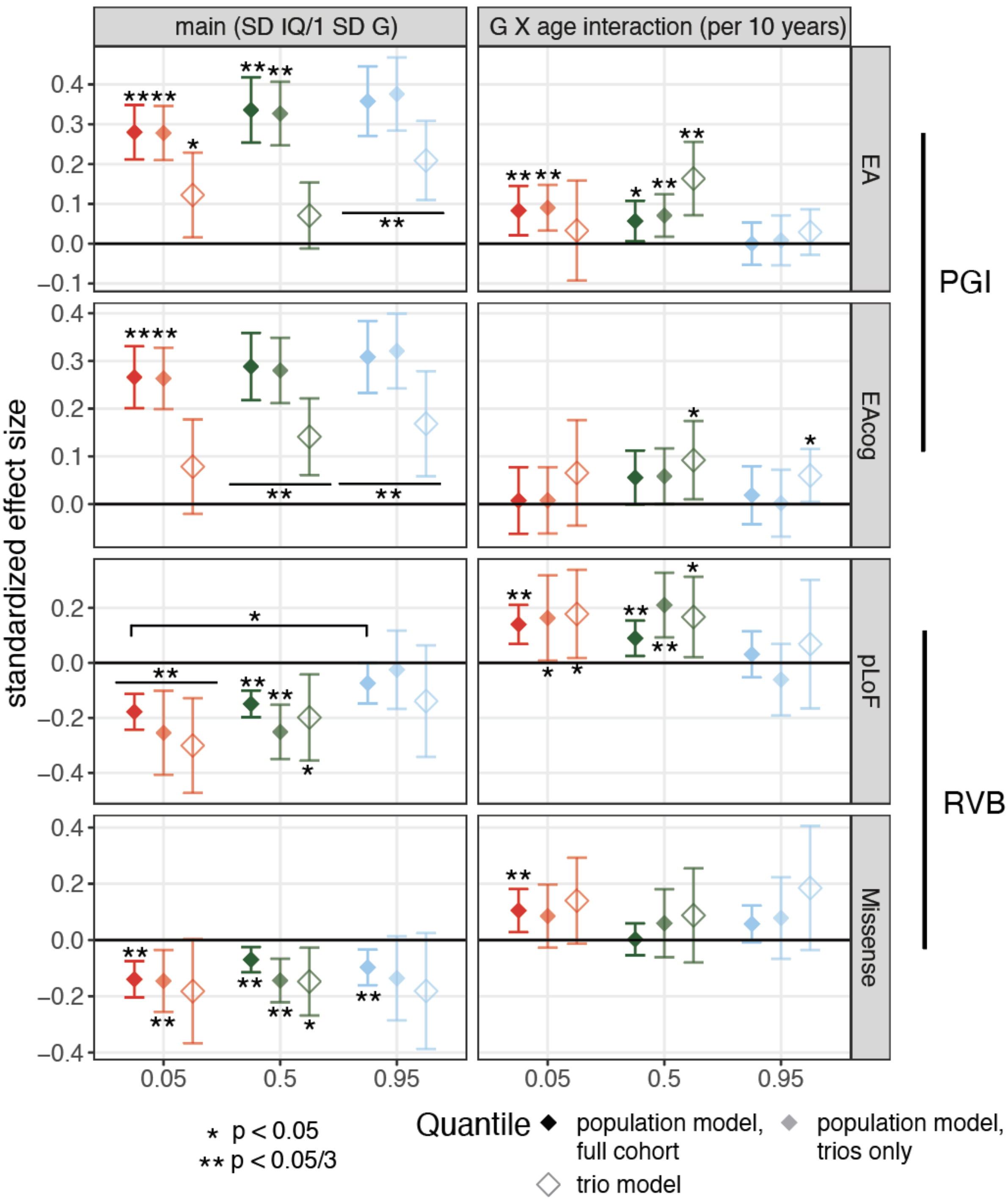

Association of common and rare variants with different quantiles of the IQ distribution pre-imputation. (As for Figure 3, which is based on post-imputation IQ. Standardized effect sizes and 95% confidence intervals for quantile regression of the 5th, 50th, and 95th percentiles estimated from

mixed-effects modeling with pre-imputation IQ at ages 4, 8, and 16 for PGI_{EA}, PGI_{Cog}, $RVBS_{pLoF}$ and $RVBS_{Missense}$ before (left) and after (right) controlling for parental genetic measures. The square brackets indicate significant comparisons highlighted in the text (z tests).

Supplementary Figure 7

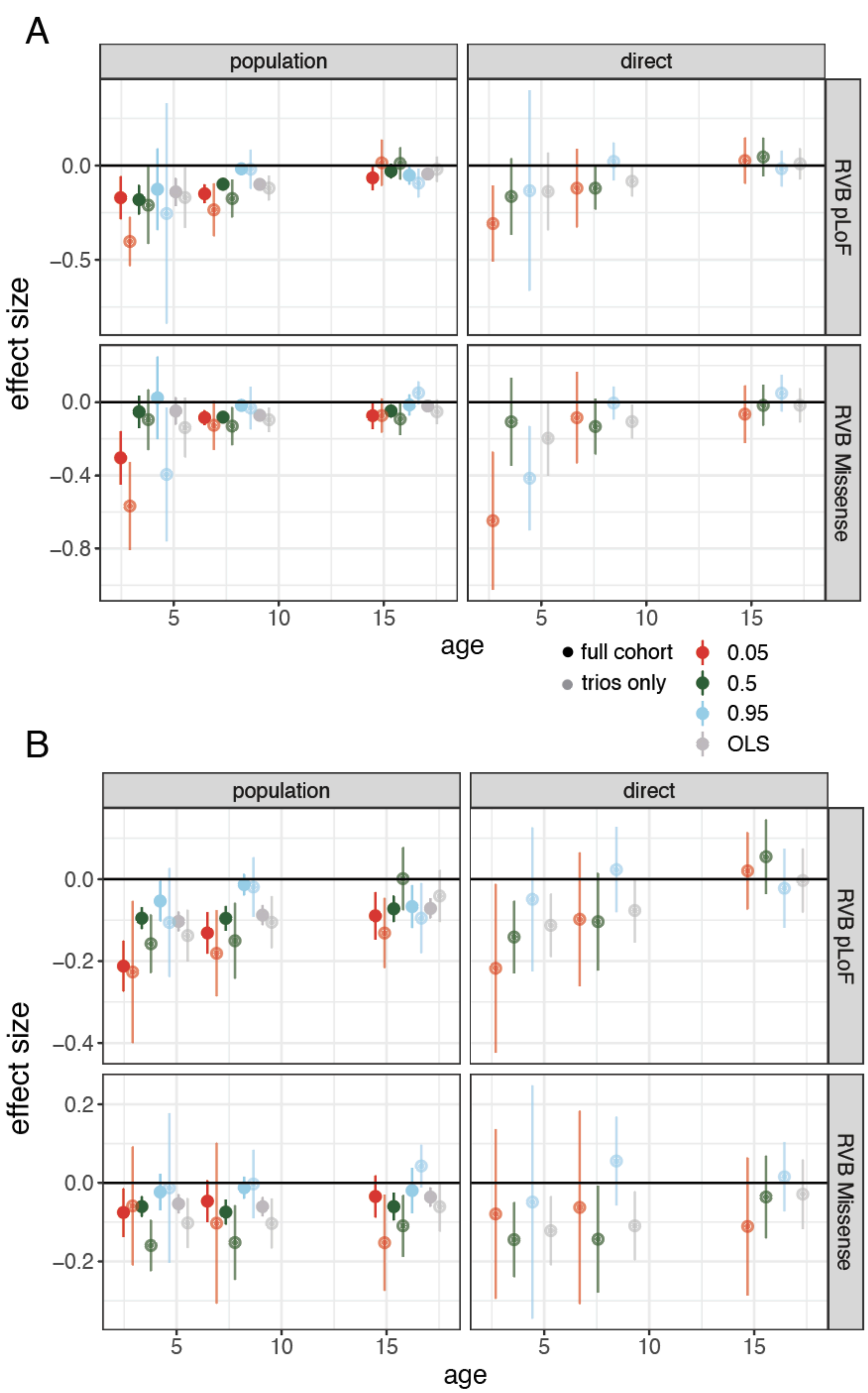

Association of RVB with different quantiles of the IQ distribution using pre-imputation IQ measures in ALSPAC, from cross-sectional analyses. A) Standardized effect sizes and 95%

confidence intervals for quantile regression of the 5th (red), 50th (green), and 95th (blue) percentiles and ordinary least squares (OLS) regression (gray) estimated from cross-sectional associations with pre-imputation IQ at ages 4, 8, and 16 for PGI_{EA} and PGI_{Cog} before (left) and after (right) controlling for parental genetic measures. B) Same as (A) using post-imputation IQ measures.

Supplementary Figure 8

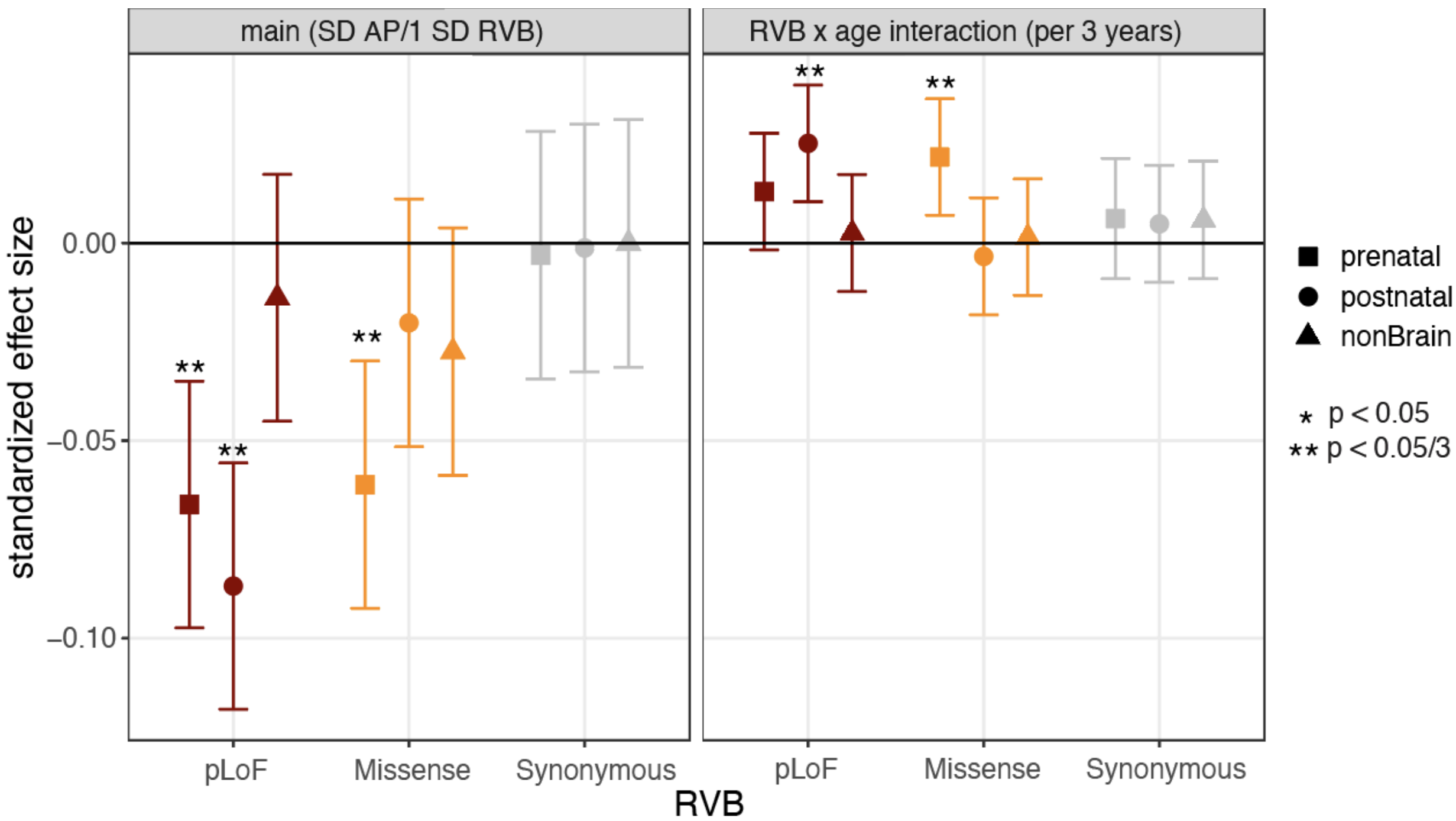

Associations between RVB in gene sets defined according to their expression patterns and academic performance in ALSPAC. (Similar to Extended Data Figure 4A which shows results for IQ in ALSPAC.) Standardized effect sizes and 95% confidence intervals estimated for main effects on academic performance and RVB-by-age-interaction effects for each RVB in three mutually exclusive gene sets from Li et al. These comprise genes in co-expressed clusters that are more highly expressed in prenatal or postnatal brain or that are not detected in the study (non-brain).

Supplementary Figure 9

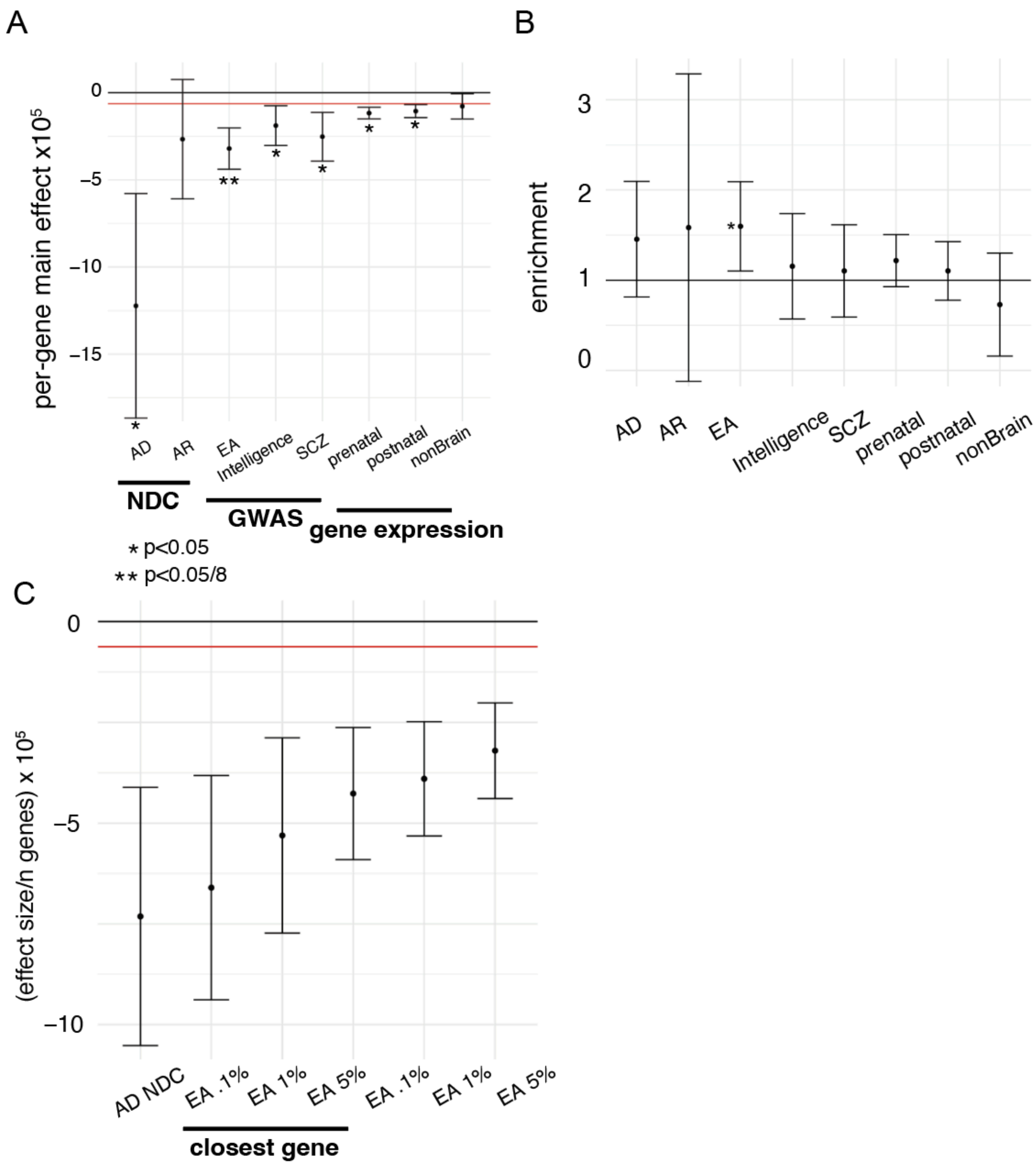

Replication of effects of gene set-specific RVB on composite cognitive performance measure in MCS. (Similar to Extended Data Figure 4BDE which show results using the main effect from mixed-effect models fitted on IQ in ALSPAC.) A) Associations of RVB_{pLoF} on composite cognitive performance score stratified by gene set, divided by the number of genes in the given gene set, with 95% confidence intervals. Red horizontal line indicates the average effect size for RVB_{pLoF} across all genes. Asterisks indicate the p-value for difference in per-gene effect sizes between a given gene set and all genes using a z test, with * indicating nominal significance and ** indicating Bonferroni significance for 8 tests. B) Ratio of the main effect for RVB_{pLoF} for the indicated gene set relative to randomly sampled gene sets with matching underlying s_{het} distributions (enrichment). C) Per-gene main effect for RVB_{pLoF} using different gene FDR threshold cutoffs based on gene prioritization in Lee et al. and by restricting to prioritized genes at a given cutoff that are also the nearest genes to the prioritizing SNP in the Lee et al. GWAS[8]. AD/AR

NDC: Autosomal dominant/recessive neurodevelopmental disorder genes with loss-of-function mechanism from DDG2P[9], EA 5%: educational attainment GWAS prioritized genes by Lee et al.[8] at 5% FDR threshold, IQ: intelligence GWAS prioritized genes from Savage et al.[10], SCZ: schizophrenia GWAS prioritized genes from Pardiñas et al.[11]

Supplementary Figure 10

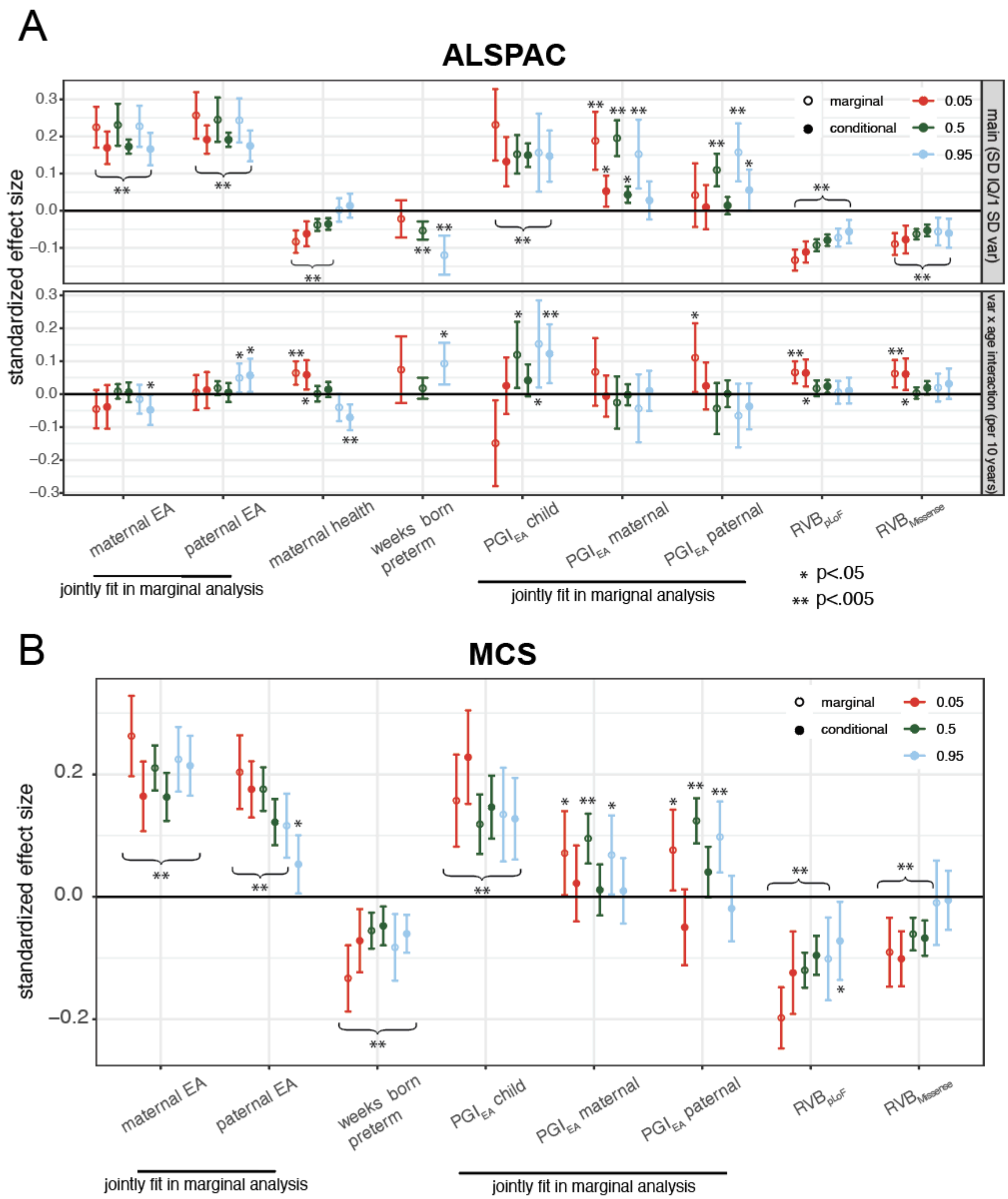

Associations between genetic and other factors and cognitive performance using quantile regression. A) ALSPAC: Standardized effect sizes and 95% confidence intervals for the main effects (top) and measure-by-age-interaction effects (bottom) for quantile regression of the 5th, 50th, and 95th percentile estimated from mixed-effects quantile regression modeling for IQ post-imputation using both marginal and conditional associations as in Figure 4. B) MCS: Standardized effects and 95% confidence intervals using a quantile regression model for quantile regression of the 5th, 50th, and 95th percentile. Asterisks indicate whether the estimate is significantly different from 0; note that curly brackets with asterisks indicate that the estimates spanned by the bracket are all significant.

Supplementary Figure 11

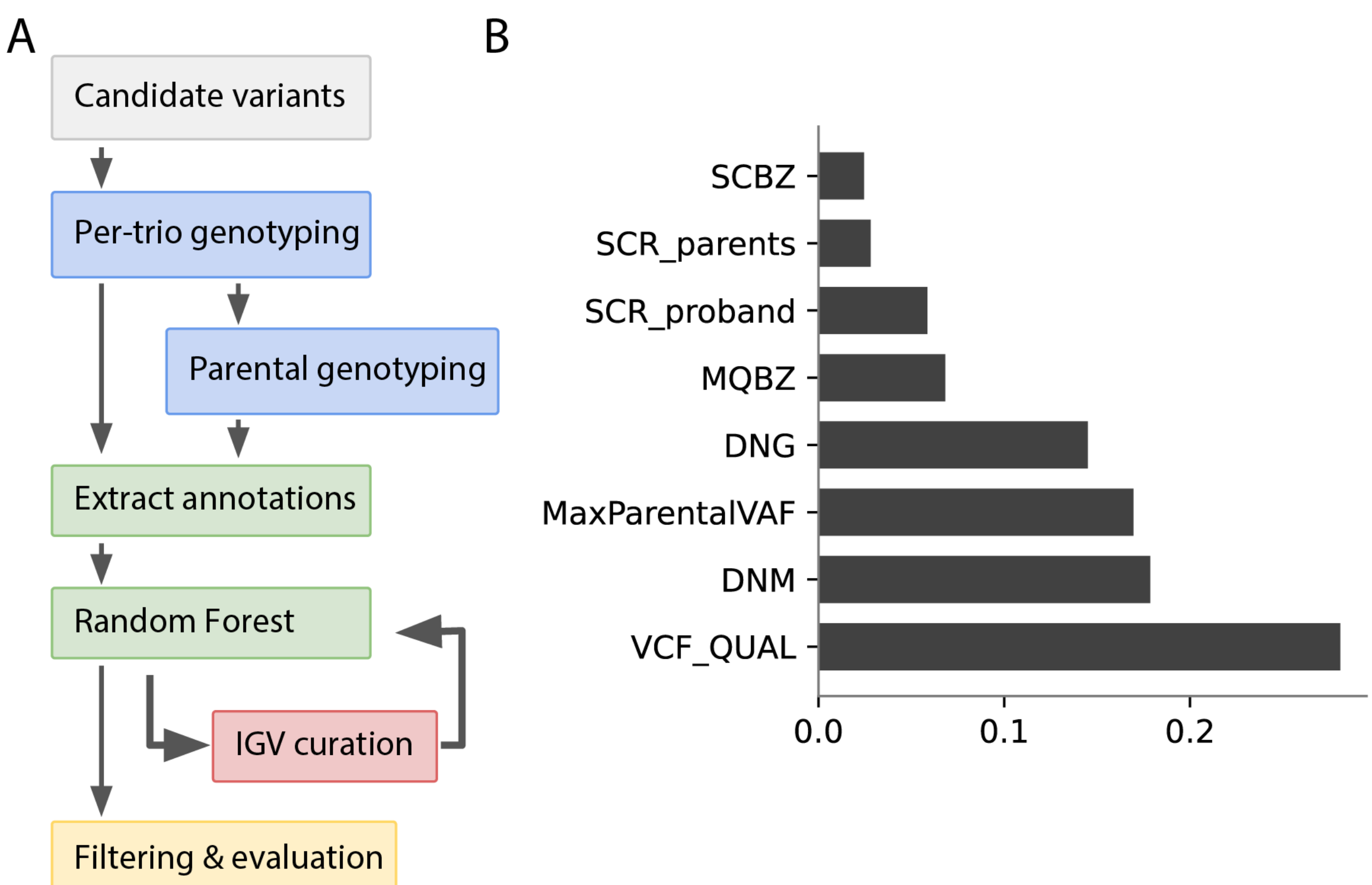

Calling and quality control of *de novo* mutations. A) Flow diagram illustrating the calling and quality control process. B) Relative importance of features included in the random forest model used for quality control. SCBZ: Mann-Whitney U-z test of number of soft-clips in variant reads; SCR: number of soft-clipped reads; MQBZ: Mann-Whitney U-z test of mapping quality of variant reads; DNG: DeNovoGear score; MaxParentalVAF: maximum variant allele frequency (VAF) observed across parents at this site; DNM: trio-dnm2 score; VCF_QUAL: variant quality as presented in the VCF QUAL column from GATK.

Supplementary Figure 12

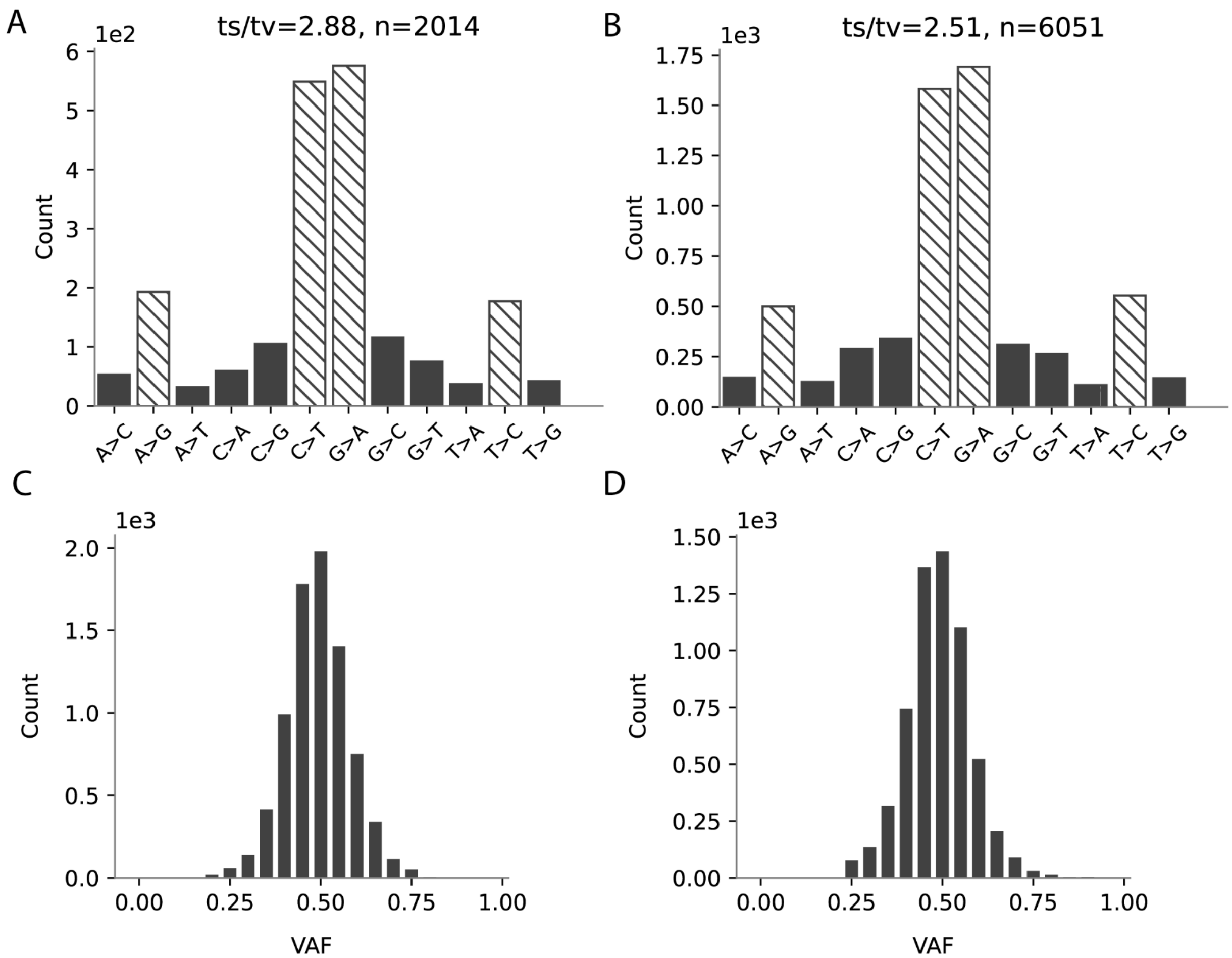

***De novo* mutation spectrum for single nucleotide variants in ALSPAC (A) and MCS (B).** The hashed bars are transitions and the black bars transversions. ts/tv = transition/transversion ratio. Distribution of variant allele fraction (VAF) in ALSPAC (C) and MCS (D).

Supplementary Figure 13

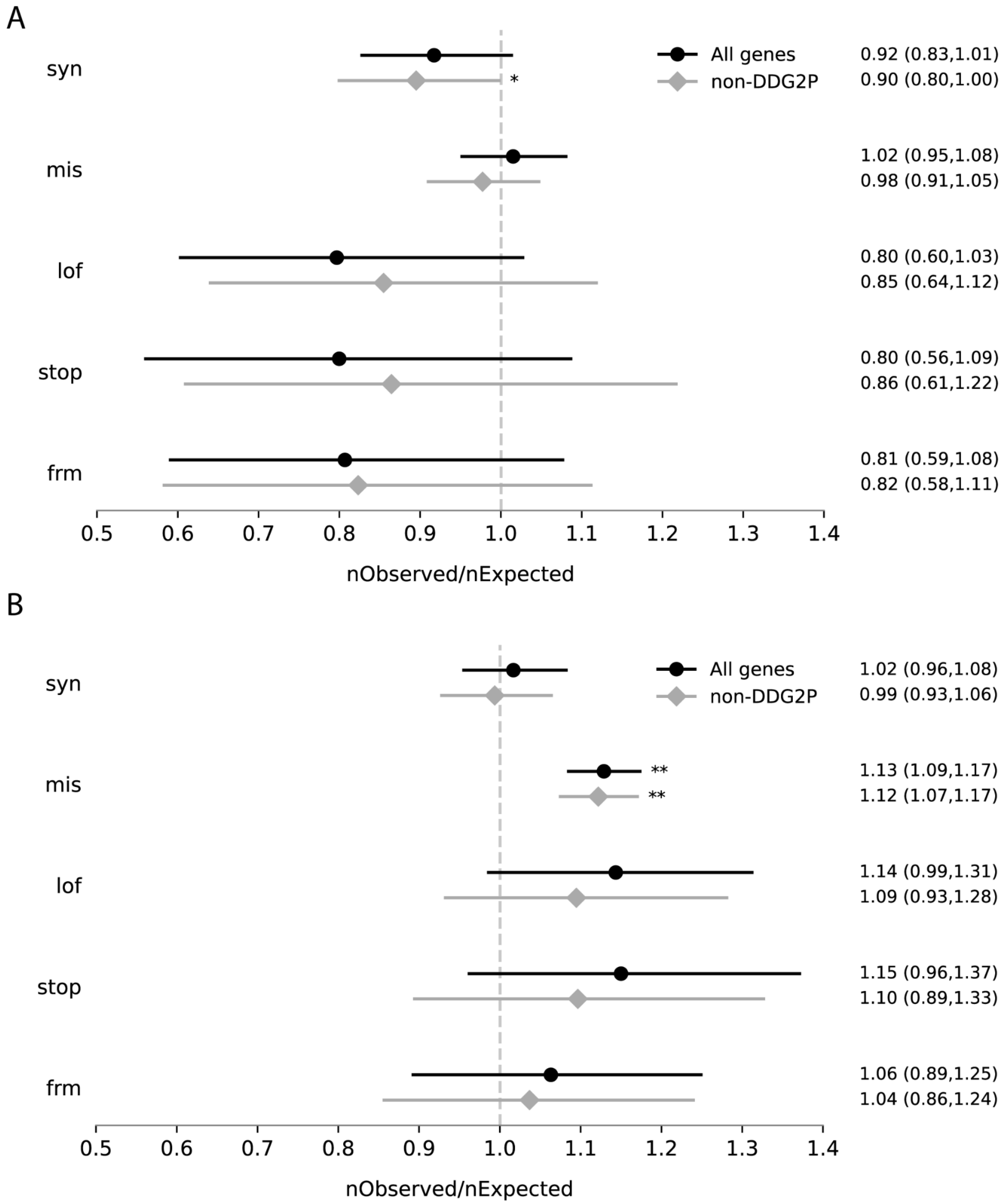

Observed vs expected number of de novo mutations in ALSPAC (A) and MCS (B). Bands show 95% confidence intervals and Poisson test significance threshold of p<0.05 (*) and p<0.01 (**). The considered mutations types were: synonymous (syn), missense (mis), loss of function (lof), stop gained (stop) and frameshift indels (frm).

Supplementary Note 1: IQ imputation in ALSPAC

Imputing IQ using SoftImpute

IQ was measured in ALSPAC at ages 4, 8, and 16 via psychometric testing administered in an in-person visit. Of the 8,804 children in the study with SNP array data, 6,495 had IQ measured at least once, with the lowest attendance at age 4 and relatively higher attendance at ages 8 and 16 (Extended Data Figure 1A). IQ measures were highly correlated between ages, with pairwise correlations ranging from 0.51 (between ages 4 and 16 $t(444) = 12.67$, $p = 1.25 \times 10^{-31}$, $r = 0.51$, 95% CI = [0.444, 0.580]; Pearson correlation) to 0.63 (between ages 4 and 8 $t(584) = 19.80$, $p = 3.90 \times 10^{-67}$, $r = 0.634$, 95% CI = [0.583, 0.680]). To characterize the extent of nonrandom missingness among children with at least one IQ measurement, we compared the distribution of PGIs and RVBs among the different combinations of missingness by coding each individual's missingness pattern as categorical variable (e.g., "IQ measured only age 8", "IQ measured at age 4 and 8", etc.). We specifically assessed nonrandom missingness with respect to the genetic scores since collider bias[12] would only be induced if the variable of interest (in this case PGIs and RVB) is associated with ascertainment. We found small though significant associations between the IQ missingness pattern and all PGIs ($F(6, 5083) = 16.96$, $p = 1.71 \times 10^{-19}$, $R^2 = 1.96\%$, 95% CI = [0.0119, 0.0268];; ANOVA for PGI_{EA}, $F(6, 5083) = 6.42$, $p = 9.43 \times 10^{-7}$, $R^2 = 0.75\%$, CI = [0.0027, 0.0118] for PGI_{Cog}, and $F(6, 5122) = 4.79$, $p = 7.08 \times 10^{-5}$, $R^2 = 0.56\%$, 95% CI = [0.0015, 0.0092] for PGI_{NonCog}). In general, those with fewer tests had lower mean PGIs than those with more than one test completed, and those with age 8 measurements had the lowest mean PGI_{EA} and PGI_{Cog} (Extended Data Figure 1C, Supplementary Table 10). In contrast, we found no association between missingness pattern and any RVB ($\chi^2(6) = 12.01$, $p = 0.066$, $\eta^2 = 0.0022$, 95% CI = [0.0000, 0.0042]; approximative K-sample Fisher-Pitman permutation test for RVB_{pLoF}, $\chi^2(6) = 3.35$, $p = 0.765$, $\eta^2 = 0.0006$, 95% CI = [0.0000, 0.0012] for $RVB_{Missense}$, $\chi^2(6) = 6.97$, $p = 0.327$, $\eta^2 = 0.0013$, 95% CI = [0.0000, 0.0026] for $RVB_{Synonymous}$). However, it is possible that we were underpowered to detect associations between RVB and missingness due to the relatively low variance explained by RVB on relevant phenotypes. These results suggest that, although subtle, nonrandom missingness may influence our findings via collider bias especially in the pre-imputation analyses. To estimate the extent of this bias, we conducted various simulation studies and robustness analyses (e.g., Supplementary Note 8).

In order to address nonresponse bias and increase our phenotyped sample size, we imputed missing IQ values across ages for all individuals with at least one IQ measurement and at least 20% of variables considered for the imputation having nonmissing values per individual using SoftImpute[13], an imputation algorithm that leverages the correlation structure across selected related variables to simultaneously impute all missing phenotype values. We first sought to identify a set of variables that maximize the imputation accuracy. We assessed three groups of variables: 1) the two other IQ measures, sex, parental income, and birth weight (base set), 2) the base set as well as a range of variables measured across life including additional cognitive and behavioral assessments (expanded set) (see Methods), and 3) the expanded set without the base set of variables (auxiliary set). To assess imputation quality for each of these three

imputation strategies, we masked 100 measured IQ values at a given age and compared how the measured values compare to the imputed values.

The highest correlations were observed in the expanded set across ages (Extended Data Figure 1B). Importantly, we observed a median correlation of 0.61 for imputation accuracy for IQ at age 4, where we have the fewest phenotyped individuals. When using the auxiliary set of variables, the performance at age 4 and age 8 was similar to that when using the base set, but performance was markedly worse at age 16 with a median correlation difference of 0.24. These results suggest the auxiliary variables improved imputation most in the younger ages likely due to most of them being measured early in childhood, while at age 16 the base set contained the majority of the information that informed the imputation. Reassuringly, we observed consistent and accurate imputation across ages and used imputed IQ derived from the expanded set of variables throughout.

Common variant heritability and genetic correlations of IQ across time

We then compared the common variant genetic architecture of the imputed IQ values across ages. We conducted genome-wide association studies (GWASs) on IQ measurements at each age in an unrelated set of individuals inferred to have European genetic ancestry (n=6,495) (Methods). We used GREML-LDMS to calculate heritabilities and genetic correlations between the three phenotypes. We found significant heritabilities for all traits, with h^2 estimates between 0.46-0.56 (IQ age 4: Z = 5.68, p = 1.35×10^{-8}, h^2 = 0.46, 95% CI = [0.30, 0.62]; GREML, IQ age 8: Z = 6.00, p = 1.97×10^{-9}, h^2 = 0.48, 95% CI = [0.32, 0.64] IQ age 16: Z = 7.00, p = 2.56×10^{-12}, h^2 = 0.56, 95% CI = [0.40, 0.72]) (Supplementary Table 2). Additionally, we found significant genetic correlations between all of the traits, with none showing significant difference from an r_g = 1 (IQ age 4 – IQ age 8: Z = 46.19, r_g = 0.97, 95% CI = [0.93, 1.01]; bivariate GREML and test for $r_g \neq 1$: Z = -1.43, p = 0.1531, IQ age 4 – IQ age 16: Z = 40.00, r_g = 0.96, 95% CI = [0.91, 1.01]; GREML, Test for $r_g \neq 1$: Z = -1.67, p = 0.0956, IQ age 8 – IQ age 16: Z = 30.97, rg = 0.96, 95% CI = [0.90, 1.02]; test for $r_g \neq 1$: Z = -1.29, p = 0.1969) (Supplementary Table 2). These estimates of childhood IQ are concordant with previous estimates of SNP heritabilities ranging from 0.22-0.72[14,15].

We additionally used LD score regression to calculate genetic correlations between the three GWASs and external GWASs of EA[8] and the cognitive and non-cognitive components of EA (EA-Cog and EA-nonCog)[16]. We found all genetic correlations were significant, with the correlations for EA and EA-Cog being consistently high (r_g > 0.85) (Supplementary Table 3). In contrast, the genetic correlations with EA-nonCog were lower (0.42-0.63) and significantly less than 1, consistent with the lower genetic correlation estimates between childhood IQ and EA-nonCog relative to that with EA-Cog previously observed[16]. Overall, these results indicated the common variant genetic architecture of measured+imputed IQ was highly similar across ages and to those of cognitive performance and educational attainment GWASs in directly-measured external samples.

Supplementary Note 2: Associations of polygenic scores and rare variant burden on academic performance measures in ALSPAC

As a complementary approach, we assessed the association of the PGIs and RVBs with academic performance in ALSPAC. In Year 6 and Year 9 (roughly ages 11 and 14, respectively; known as Key Stages 2 and 3 in the UK) children were administered three standardized exams covering English, Mathematics, and Science from which we derived a composite academic performance metric which we showed was measuring the same latent construct across time (Supplementary Table 1; Methods). For children who had complete data for the three exams at both ages (n=3,895), we assessed the contribution of the three PGIs to academic performance in a similar way to that described for IQ in the main text. We found significant population effects for all PGIs and significant increases in effects with age for PGI_{EA} and PGI_{NonCog} (Extended Data Figure 2A, Supplementary Table 5). In a trio analysis (n=3,024), we found evidence for direct genetic effects of PGI_{EA} and PGI_{Cog} on academic performance (Extended Data Figure 2A, Supplementary Table 5), consistent with previous work in other cohorts[7]. Though all of the PGI-by-age interactions were positive, only the PGI_{EA} showed a significant increase in direct effects (Supplementary Table 5), likely due to the reduction in power of the decreased sample size and the narrower age range considered. However, broadly these results for the impact of common genetic variation on academic performance mirror what we observed for IQ. For RVB_{pLoF} and $RVB_{Missense}$, we found that they were significantly negatively associated with academic performance at age 11 but their associations attenuated with age (Extended Data Figure 2B, Supplementary Table 7).

Supplementary Note 3: Differential associations of rare variants in different gene sets on IQ

We assessed whether the RVB associations with IQ differed according to the expression patterns of the genes in which they lie. Prior GWAS studies suggest that genes overlapping loci associated with intelligence and educational attainment are enriched for expression in brain[8,10], as expected, and that genes associated with educational attainment showed on average relatively higher expression in prenatal rather than postnatal brain[8]. Similarly, genes implicated in autism by rare variants are also enriched for expression in prenatal brain [17], as are those associated with neurodevelopmental disorders[18]. We recalculated RVB stratified by genes' assignments to co-expressed modules enriched for expression in prenatal brain (n genes = 6,808), postnatal brain (6,078), or undetected in brain (3,028)[19]. We found that RVBs in the non-brain genes were not significantly associated with IQ (Extended Data Figure 4A). In contrast, RVB_{pLoF} and $RVB_{Missense}$ in the prenatal and postnatal gene sets were associated with IQ with main effects of similar magnitudes (RVB_{pLoF} in prenatal genes post-imputation main effect: $Z = -6.30$, $p = 2.92 \times 10^{-10}$, $\beta = -0.076$, 95% CI = [-0.099, -0.052]; Wald test, RVB_{pLoF} in postnatal genes post-imputation main effect: Post-imputation main effect: $Z = -5.60$, $p = 2.18 \times 10^{-8}$, $\beta = -0.067$, 95% CI = [-0.091, -0.044], $RVB_{Missense}$ in prenatal genes post-imputation main effect: $Z = -3.82$, $p = 1.31 \times 10^{-4}$, $\beta = -0.046$, 95% CI = [-0.070, -0.022], $RVB_{Missense}$ in postnatal genes post-imputation main effect: $Z = -3.30$, $p = 9.57 \times 10^{-4}$, $\beta = -0.040$, 95% CI = [-0.064,

-0.016]). In the prenatal gene set, RVB_{pLoF} had a significant interaction with age pre- and post-imputation (Pre-imputation age interaction: Z = 3.34, p = 8.24 × 10^{-4}, β = 0.006, 95% CI = [0.002, 0.010], Post-imputation age interaction: Z = 3.24, p = 1.20 × 10^{-3}, β = 0.002, 95% CI = [0.001, 0.003]) and though $RVB_{Missense}$ had a non-significant interaction post-imputation (Pre-imputation age interaction: Z = 1.87, p = 0.0620, β = 0.003, 95% CI = [-0.0002, 0.006], Post-imputation age interaction: Z = 2.30, p = 0.0213, β = 0.002, 95% CI = [0.0002, 0.003]). In contrast, we found only a no significant age interaction for RVB_{pLoF} in postnatal genes post-imputation (Post-imputation age interaction: Z = 2.13, p = 0.0335, β = 0.001, 95% CI = [0.0001, 0.003]), and no evidence for an age interaction pre-imputation (Pre-imputation age interaction: Z = 0.72, p = 0.4730, β = 0.001, 95% CI = [-0.002, 0.004]). Hence, rare damaging variants in genes preferentially expressed in the prenatal brain showed a clear attenuation of the RVB association with age on IQ, but there is only mixed evidence for an age interaction among genes preferentially expressed in postnatal brain.

Similarly, we then assessed the association between RVBs stratified by expression patterns in the brain and academic performance in ALSPAC. As before, we detected no significant associations with any $RVB_{Synonymous}$ and or RVB calculated in genes not detected in brain (Supplementary Figure 8). We found significant associations between RVB_{pLoF} and $RVB_{Missense}$ calculated in prenatal genes and academic performance, and RVB_{pLoF} calculated in postnatal genes. However, the patterns of attenuation were less clear: RVB_{pLoF} only showed significant attenuation with age when calculated in postnatal genes and $RVB_{Missense}$ only showed a significant attenuation in prenatal genes. The confidence intervals on the age interaction estimates are large, particularly for the positive age interaction for RVB_{pLoF} calculated in prenatal genes where the estimate was not significant, which could have been due to statistical power limitations.

We next explored other gene sets that we hypothesized might have differential contributions to the RVB_{pLoF} association with IQ. In addition to the aforementioned gene sets defined by expression pattern, we considered gene sets ascertained for severe monogenic autosomal dominant (n genes = 337) or recessive NDCs (636) and genes prioritized via common variant genome-wide association studies of EA[8] (1,838), cognitive ability (1,912), and schizophrenia[11] (1,558). We calculated RVB_{pLoF} subsetted to only genes in a given gene set, and considered the main effect on IQ in ALSPAC estimated in a mixed-effects model, divided by the number of genes in the gene set, yielding per-gene effect size estimates (Extended Data Figure 4B). Of the gene sets defined based on expression in the brain, only the prenatal gene set showed a modestly greater per-gene effect size than the exome-wide average (Per-gene enrichment (2.0x exome-wide): Z = -3.01, p = 0.0026, Δ per-gene β = -1.06 × 10^{-5}, 95% CI = [-1.75 × 10^{-5}, -3.70 × 10^{-6}]; z test). The autosomal dominant NDC gene set had the largest per-gene effect size, with the average per-gene effect being 12.3 times the exome-wide average (Z = -1.98, p = 0.024, β = -0.0230, 95% CI = [-0.046, 0.000]), though not significantly different from it due to the large standard error of the estimate(Z = -1.82, p = 0.0686, Δ per-gene β = -1.69 × 10^{-4}, 95% CI = [-3.50 × 10^{-4}, 1.29 × 10^{-5}]). In contrast, we found no evidence for the autosomal recessive gene set having significant association with IQ (Z = -1.69, p = 0.091, β = -0.0200, 95% CI = [-0.043, 0.003]). The next strongest association was that of the EA GWAS gene set (Z = -6.06, p = 1.34

$\times 10^{-9}$, $\beta = -0.0356$), with an average per-gene effect 7 times greater than the exome-wide average ($Z = -5.27$, $p = 1.34 \times 10^{-7}$, Δ per-gene $\beta = -6.79 \times 10^{-5}$, 95% CI = $[-1.01 \times 10^{-4}, -4.64 \times 10^{-5}]$).

The differences in average rare variant effect sizes per gene between gene sets observed in could simply be reflecting their different distributions of underlying selection coefficients (Extended Data Figure 4C), since the majority of the RVB_{pLoF} signal is due to variants in highly constrained genes (Extended Data Figure 5A). In order to assess the enrichment of per-gene effects in a gene set relative to similarly constrained genes, for each gene set, we simulated 100 gene sets of equal size with matching underlying selection coefficient distributions and pooled their effect estimates (see Supplementary Methods). Enrichment was defined as the ratio of the effect estimate for the original gene set to the pooled estimate from the randomly sampled gene sets. The autosomal dominant NDC gene set did not show any enrichment, suggesting that the relatively high number of evolutionarily constrained genes in this gene set is the main driver of the large per-gene effect (Extended Data Figure 4D). In contrast, the only Bonferroni-significant enrichment was observed for the EA GWAS gene set, with nominal enrichments for the intelligence and prenatal gene sets and a relative depletion of signal in the non-brain gene set (Extended Data Figure 4D). We observed similar results using the composite cognitive performance measure in MCS, albeit with attenuated levels of enrichment compared to ALSPAC that were nominally significant only for the EA GWAS gene set (Supplementary Figure 9). These results suggest that genes prioritized via the EA GWAS contribute more variance to the RVB associations with childhood IQ than expected given their level of constraint, and potentially also for those prioritized via the intelligence GWAS and genes preferentially expressed in prenatal brain.

Given the large enrichment and per-gene effects observed for the RVB_{pLoF} in the EA gene sets, we then sought to compare how different gene inclusion criteria impact their associations on IQ in ALSPAC. We previously used a 5% FDR cutoff from the prioritized genes in Lee et al. regardless of whether the gene was the closest gene to the significant SNPs informing the prioritization. We additionally considered all combinations of using a 1% and 0.1% FDR cutoff as well as restricting to only genes that were the closest genes to the GWAS significant SNPs, resulting in six gene sets. Both when considering all genes or only closest genes, using more stringent FDR thresholds led to stronger per-gene standardized effects (Extended Data Figure 4E). When restricting to only closest genes, the point estimates of the effects increased substantially for all FDR thresholds. For example, when comparing the gene sets with an FDR cutoff of 0.1%, the per-gene standardized effects increased by 51% when restricting to the closest genes, i.e. the per-gene variance explained was 2.3 times greater, though the standard errors increased substantially due to the decrease in the gene set size (Extended Data Figure 4E). As there was modest overlap with the AD NDC gene set, we further considered the EA gene sets after excluding genes overlapping the AD NDC gene set to ensure they were not driving the associations. After doing so, the per-gene effect point estimates all became stronger though not significantly so (Extended Data Figure 4E), likely due to the relative rarity of damaging variants in the highly constrained NDC genes. These results were recapitulated using a composite measure of cognitive performance in MCS (Supplementary Figure 9C). We

conclude that genes prioritized via common variant GWAS of cognitive traits in adult populations converge with those that have outsized effects on IQ in early life.

Supplementary Note 4: Relative contributions of deleterious pLoF *de novo* and inherited variants to IQ

We sought to compare the relative contribution of inherited versus *de novo* pLoF variants in evolutionarily constrained genes (n = 3160 autosomal genes) to IQ in ALSPAC and our composite cognitive performance measure in MCS. To simplify interpretability of the influence of *de novo* variants in such genes on these phenotypes, we first compared the association of the RVB_{pLoF} previously used to the association of the the number of evolutionarily constrained genes in which an individual carries a pLoF variant (constrained pLoF count). The standardized effect size of RVB_{pLoF} and constrained pLoF count on IQ were not significantly different from each other at any age in ALSPAC, though the latter consistently produced slightly weaker effect sizes (Extended Data Figure 5A). Thus, for this analysis, we considered the associations of constrained pLoF count with measures of cognition.

We then compared the unstandardized effect sizes of the constrained pLoF count of putatively inherited versus robust *de novo* variants on IQ in the fully exome-sequenced trios from ALSPAC (n=958) using mixed-effect modeling. The *de novo* constrained pLoF count had a larger point estimate, but it was not significantly different from that for inherited variants (Extended Data Figure 5B). However, power may have limited the ability to estimate a difference in the effect sizes, as only 1.8% of children had at least one *de novo* pLoF in a constrained gene, compared to 12% with at least one inherited pLoF in a constrained gene. The age interaction with *de novo* constrained pLoF count was nonsignificant, though in a concordant direction with the inherited pLoF count and the total pLoF constrained count (Extended Data Figure 5B). The ratio of the main effect to the age interaction effect was highly concordant between *de novo* mutations and inherited variants (18.6 and 17.9, respectively), potentially suggesting the age attenuation of the association with rare variants acted similarly for *de novo* and putatively inherited variants, though the error bars are large.

The variance of the constrained pLoF count for *de novo* mutations was significantly lower than that for inherited variants (0.018 versus 0.14, $F(957, 957) = 7.78$, $p = 1.16 \times 10^{-190}$, variance ratio = 7.78, 95% CI = [6.85, 8.83]; F test). The variance in IQ explained by the constrained pLoF count of inherited variants was nearly equal to that of all variants and about four times that of the *de novo* pLoF count (Extended Data Figure 5C). As the inherited and *de novo* constrained pLoF counts were uncorrelated ($t(956) = -1.24$, $p = 0.2161$, $r = -0.04$, 95% CI = [-0.103, 0.023]; Pearson correlation) and the estimated age interaction effects were proportionally consistent, these results suggested that the inherited constrained pLoF count explained between 3.5-4 times more variance than the *de novo* constrained pLoF count in IQ across development in this cohort. Virtually identical results were observed when conducting the same analysis on cognitive performance in MCS (Supplementary Figure 4).

However, this analysis has several limitations. First, our *de novo* variant calling was calibrated to maximise specificity, possibly at the expense of sensitivity, so some of the putatively inherited variants may in fact be *de novo*. Second, the fact that children in full trios in ALSPAC and MCS are biased towards coming from households with higher educational attainment (e.g. association of maternal and paternal EA with being in a full trio in ALSPAC, maternal: $Z = 9.92$, $p = 3.51 \times 10^{-23}$, OR = 1.079, 95% CI = [1.063, 1.095]; logistic regression (Wald test), paternal $Z = 11.77$, $p = 5.59 \times 10^{-32}$, OR = 1.086, 95% CI = [1.071, 1.101]) and older parents (maternal age $Z = 2.72$, $p = 0.0065$, OR = 1.029, 95% CI = [1.008, 1.050]; logistic regression (Wald test) in ALSPAC; $Z = 7.82$, $p = 5.00 \times 10^{-15}$, OR = 1.078, 95% CI = [1.058, 1.098] in MCS) likely means we have overestimated the fraction of the rare variant score association that is due to *de novo* mutations compared to an unbiased sample. This implies that the trio parents are likely to have fewer inherited rare variants reducing cognitive ability and that they probably pass on more *de novo* mutations due to having, on average, higher parental age[20,21].

Supplementary Note 5: Quantile regression results using cognitive ability measures in MCS and UK Biobank

As additional replication for the quantile regression results (Figure 3), we then considered the association between PGI_{EA} and a single measure of cognitive performance from each of MCS and UK Biobank. In MCS, cognitive tests were administered at multiple ages, but previous work showed that these are not longitudinally invariant[22], so we instead extracted a single composite cognitive measure from the tests administered at ages 3 and 7 (see Methods) to represent overall cognitive performance in early childhood. In UK Biobank, we used the results for the verbal-numerical reasoning test (sometimes called “fluid intelligence”) conducted at baseline, to represent adult cognitive performance. We hypothesized that we would see relatively uniform associations across quantiles of early childhood cognitive performance measured in MCS, as we did for IQ in ALSPAC at age 4 (Supplementary Figure 5B). Given the differential age interactions across quantiles observed in ALSPAC (namely the increasing association with age that is seen only in the top half of the distribution), we predicted that, in UK Biobank adults, the PGI_{EA} associations would be markedly stronger at the top 5% and median than the bottom 5%. Our results were concordant with these two predictions (Extended Data Figure 7B): neither the population (n=5,920) nor direct (n=5,309) effects were statistically different across quantiles in MCS, while we found significant heterogeneity in UK Biobank when examining both the population effect (n=101,232) and direct effect (n=11,859), with the direct effect at the top 5% being 1.62 times greater than that at the bottom 5% (p=0.0084).

We then considered the association between the RVB_{pLoF} and $RVB_{Missense}$ and measures of cognitive performance in MCS (n=5,666) and UK Biobank (n=101,232). Given the heterogeneity of associations with IQ observed at age 4 in ALSPAC (Supplementary Figure 7B), we hypothesized that in MCS, we would find stronger associations for RVB_{pLoF} at the bottom 5% of our composite cognitive performance measure from early childhood. In UK Biobank, we predicted that the differences across quantiles would be minimal since we observed increasingly

uniform associations across the quantiles by age 16 in ALSPAC (Supplementary Figure 7B). Our findings were concordant with these two predictions: the bottom 5% had an association 1.82-times stronger than the median and 2.4-times stronger than the 95th percentile in MCS (p=0.019 and 0.011, z-test, respectively), while we found no significant differences in associations across quantiles in UK Biobank (Extended Data Figure 7B). Collectively, these results support our PGI and RVB findings using ALSPAC IQ measures.

Supplementary Note 6: Results of models including genetic measures, parental education, and perinatal exposures

We first considered the associations of genetic scores, parental education and perinatal factors (maternal health and weeks born preterm) with mean IQ in ALSPAC using mixed-effects linear regression. Since perinatal factors considered are both associated with lower parental EA[23–25], and parental EA is correlated with both the parents' and the child's genetics, the associations of these various factors on the child's cognitive ability were likely not independent. Thus, we considered a model in which we jointly fit parental EA and the perinatal exposures together with the genetic scores that showed significant associations in Figure 1 and 2, namely offspring and parental PGI_{EA} and the child's RVB_{pLoF} and $RVB_{Missense}$ (conditional estimates shown in orange in Figure 4). For replication, we ran a linear regression of our composite cognitive performance measure in MCS against the genetic scores, parental education and weeks born preterm, omitting the 'maternal health' variable since the data were not readily available (Extended Data Figure 9).

In ALSPAC, incremental R^2 of the joint model (excluding weeks born preterm due to the lower sample size) relative to a baseline model with sex and genetic 10 PCs was 22.8% at age 4, 21.2% at age 8 (p=0.29 for a z-test for difference in variance explained compared to age 4) and 25.6% at age 16 (p=0.073 and 0.0045 relative to age 4 and 8 respectively, z test). In MCS, the incremental R^2 of the joint model was 16.7% relative to the baseline. We found that when jointly fit with the other variables, the parental PGI_{EA} associations became nonsignificant in both cohorts, though the child's direct effect estimate did not significantly change (Figure 4 top, Extended Data Figure 9). Similarly, the associations of the child's RVB_{pLoF} and $RVB_{Missense}$ with IQ did not significantly attenuate in either cohort after controlling for these different exposure variables and PGI_{EA}. The association between weeks born preterm and IQ was no longer significant in the conditional analysis in ALSPAC, though this may be due to reduced power in the joint model, whereas in MCS it remained significant. Maternal and paternal EA showed similar effect sizes to each other in both cohorts, which were also similar to those captured by the direct genetic effect of the child's PGI_{EA}. These results suggest, apart from the parental PGIs, effect size estimates were largely unaffected in a conditional analysis.

We next considered the association of genetic scores, parental education and perinatal factors with the different quantiles of the distribution. In ALSPAC, considering each of the variables separately (Supplementary Figure 10A, marginal estimates), we found that paternal and

maternal education had similar magnitudes of main effects as well as uniform associations across quantiles, and we detected a nominally significant positive age interaction for paternal education at the 95th percentile (Supplementary Figure 10A), although the latter was not replicated in MCS, potentially due to the different demographics of this cohort. We found substantial heterogeneity in the association of weeks born preterm in ALSPAC; this variable showed highly significant main effects at the 95th percentile (effect size = -0.120, $p=8.44 \times 10^{-6}$, bootstrap statistics) that significantly attenuated with age, as previously observed when examining the association with mean IQ (Figure 4 bottom), while we detected no significant associations at the 5th percentile (effect size = -0.022, p=0.389, bootstrap statistics). However, in MCS we did not find significant heterogeneity of associations with this variable at the tails (Supplementary Figure 10B). This difference may be because all the ALSPAC children in this subsample were born after 32 weeks' gestation, thus excluding very and extremely premature babies who might be expected to have the greatest cognitive deficits[26]. In contrast to the prematurity result, we detected significant main effects of maternal illness at the 5th percentile in ALSPAC that attenuated with age, while the main effect at the 95th percentile was nonsignificant (Figure 4 bottom). These results suggest that different perinatal factors may have varying impacts across the IQ distribution, with some factors predominantly affecting the upper or lower tails of cognitive ability.

We then further explored associations on the different quantiles of the IQ distribution in a joint model of the genetic and other exposures (Supplementary Figure 10A, conditional estimates), excluding "weeks born preterm" in ALSPAC due to high missingness. In ALSPAC, we detected significant maternal PGI_{EA} main effects on the 5th and 50th percentiles of the IQ distribution (Supplementary Figure 10A) which were not observed in the conditional analysis of mean IQ (Figure 4), suggesting the heterogeneous associations across the IQ distribution may have masked these associations. This result suggests that the mother's PGI_{EA}, independently of its influence on the mother's actualized EA, may be associated with the child's IQ at the lower tail of the IQ distribution either due to genetic nurture or confounding tagged by the paternal PGI_{EA}. However, we did not replicate this finding in MCS (Supplementary Figure 10B). Maternal EA did not show heterogeneity of main effects across the IQ distribution in either cohort (Supplementary Figure 10). However, in ALSPAC but not MCS, paternal EA did show a significant positive age interaction at the 95th percentile (Supplementary Figure 10A bottom) which was not observed when considering the association with mean IQ (Figure 4 bottom), suggesting the influence of paternal EA on IQ increases at later stages of development among those at the top of the IQ distribution. Thus, in summary, our results suggest that the relative influences of factors that best predict which children will have cognitive difficulties or will excel cognitively across childhood differ from those that best predict average IQ. However, given that some specific findings did not replicate across cohorts, there is a need to explore this in larger sample sizes.

Supplementary Note 7: Results of quantile regressions on genetic scores pre- versus post-imputation of IQ

The results from the quantile regressions in Figure 3 were based on IQ after imputation of missing values (Supplementary Note 1). We found that when analyzing pre-imputation IQ, some quantile regression estimates were not concordant with the observed associations post-imputation, particularly at age 4. Specifically, we found that, in cross-sectional analyses, there was no significant association between PGI_{EA} and PGI_{Cog} and IQ at the 5th percentile, but a large association with the 95th percentile (Supplementary Figure 5A, Supplementary Table 8). However, by age 8 and 16, where the missingness was substantially lower, the pre-imputation estimates were similar to the post-imputation estimates (Supplementary Figure 5A versus B). When analyzing the pre-imputation measures using mixed-effects quantile regression, this discordant association at age 4 led to an observed positive age interaction at the 5th percentile, a nominal interaction effect at 50th percentile, and none at 95th percentile (Supplementary Figure 6). However, in a direct effect analysis, the association with the 95th percentile was greatly attenuated (Supplementary Figure 6) and the age interactions generally mirrored the results observed post-imputation (Supplementary Figure 6 versus Figure 3). Specifically, when examining the direct effects, there was no age interaction at the 5th percentile for either PGI, but there were positive interactions at the 50th percentile for both PGIs and at the 95th percentile for PGI_{Cog} (Supplementary Figure 6). These results suggest that ascertainment bias most likely strongly impacted the PGI results pre-imputation. The fact that we observed no significant population effect of these PGIs on the 5th percentile of the distribution at age 4 may be because power has been reduced by ascertainment biases in the IQ test conducted at this age; of the subsample of participants invited to complete this test, only 81% accepted, and it may be that those families who declined were particularly biased towards lower educational attainment. Additionally, children born very prematurely were excluded from the sample and these were likely to have lower IQ. However, after conditioning on parental PGIs, the results were largely concordant with the post-imputation analysis.

The RVB_{pLoF} and $RVB_{Missense}$ results pre-imputation (Supplementary Figures 6-7) were qualitatively similar to the post-imputation results (Figure 3), with nominally significant larger main effects at the 5th percentile relative to the 95th and significant positive age interactions only detected at the lower half of the IQ distribution.

Supplementary Note 8: Assessing the robustness of age interaction findings to non-random attrition

As there is nonrandom missingness in the ascertainment of IQ measures (Supplementary Note 1), we sought to assess the extent to which it may bias our pre-imputation results. To do so, we conducted several simulation studies and robustness analyses.

In the first simulation study (the “constant effects model”, see Supplementary Methods), we simulated IQ using RVB_{pLoF} and PGI_{EA} at three time points using the true genetic scores of individuals. We modelled each IQ variable as a standard normally distributed variable, having a partial correlation with PGI_{EA} of 0.3 and with RVB_{pLoF} of -0.1 (both constant with age), to mimic the effect sizes we empirically observed. We then masked individual IQ scores to mirror exactly the missingness pattern observed in the real data. Following masking, we then restandardized each IQ measure to have a mean of 0 and standard deviation of 1 and fit the mixed-effect regression models as we did in the main analyses in Figures 1 and 2. We did not find any evidence to suggest that, under this data-generating model, the age interaction with PGI or RVB (which should be zero here) would be estimated with meaningful bias given the observed pattern of missingness (Supplementary Table 11).

In the second simulation study, we used the exact same parameters as before though we simulated a scenario where the effect of PGI_{EA} increased linearly with age at a rate of 0.1 per 10 years to mimic the empirical observation. We again found minimal evidence for bias (Supplementary Table 11), and found we had 58.3% power to detect the PGI_{EA} age interaction in the pre-imputation sample.

Given that we observed RVB associations at the 5th percentile attenuating with age, one might be concerned that a higher rate of attrition at the lower end of the IQ distribution could be producing an artefactual age interaction. To assess this possibility, we simulated a scenario where there is a strong effect of RVB on the lower half of the IQ distribution that is constant ages. Specifically, we modified the prior simulation to make RVB_{pLoF} have an effect of -.2 but only in individuals whose IQ value, prior to considering the RVB effect, is in the lower half of the IQ distribution. We found little evidence that the missingness pattern induced meaningful bias in either the PGI_{EA} or the RVB_{pLoF} interaction term. On average, the RVB_{pLoF} x age interaction term was slightly upward biased, but the magnitude of the bias was much lower than the empirically estimated effect sizes; in the simulations, the average RVB_{pLoF} x age interaction was 0.009 per 10 years, whereas the empirically observed RVB_{pLoF} x age interaction pre-imputation was 0.063 (Supplementary Table 11). Additionally, false positive rates were well-controlled, as is evidenced by the fact that only 2% of simulations had significant RVB_{pLoF} x age interactions. Thus, it is unlikely that, if there were true, quantile-dependent effects of RVB_{pLoF} but no age interaction effect, attrition alone could have produced the age interaction we observed.

Returning to the real data, we also refitted the mixed-effects models after excluding age 4 as that was the age with the highest missingness (Extended Data Figure 1). We found that the point estimates were largely consistent with those obtained when including age 4 both pre- and post-imputation, with most estimates remaining statistically significant despite reduced power (Supplementary Table 12). Of the nine nominally significant age interactions we observed when including all three ages, two became non-significant when restricting to ages 8 and 16, which seems likely to be due to the loss of power. These results are consistent with the replication of the age interactions we observed in school grades which were ascertained at ages 11 and 14 (Extended Data Figure 2).

Collectively, these results suggest it is highly unlikely that the differential age interaction trends observed for PGIs and RVBs are due to the ascertainment patterns of IQ measures but rather likely reflect true differential trajectories of associations across development.

Supplementary Discussion

Lack of significant non-transmitted RVB associations

We observed no attenuation for rare variant direct effects relative to their population effects, in contrast to the substantial attenuation observed for PGIs. There are several potential explanations for this. For one, a key factor may be the differential age-related dynamics we observe for these two score constructs. Given that RVB associations attenuate with age while PGI associations strengthen, the cognitive ability of parents carrying deleterious rare variants is expected to be less impacted by these variants than it was in childhood, potentially resulting in minimal genetic nurture effects through parental phenotypes. In contrast, since the PGI associations become more pronounced in adulthood, they may result in genetic nurture effects by affecting parental adult traits. Additionally, the strength of assortative mating directly influences the within-family effect size attenuations in trio models, and we observed that assortative mating patterns differ substantially between common and rare variants: there was a notable difference in the within-partner correlations for PGI_{EA} ($t(5924) = 27.83$, $p = 2.87 \times 10^{-160}$, $r = 0.34$, 95% CI = [0.32, 0.36]; Pearson correlation) and PGI_{Cog} ($t(5924) = 10.88$, $p = 2.53 \times 10^{-27}$, $r = 0.14$, 95% CI = [0.11, 0.16]) versus RVB_{pLoF} ($t(5924) = 6.64$, $p = 3.33 \times 10^{-11}$, $r = 0.086$, 95% CI = [0.06, 0.11]) and $RVB_{Missense}$ ($t(5924) = -1.54$, $p = 0.1237$, $r = -0.02$, 95% CI = [-0.05, 0.01]). Additionally, the impact of assortative mating may differ between rare and common variant analyses as it has been observed that assortative mating may occur nonuniformly across the spectrum of cognitive abilities.[27] Lastly, we note that the wider confidence intervals for rare variant estimates in the trio models limit our statistical power to detect attenuation; the point estimates alone cannot rule out modest indirect effects. Nonetheless, our findings suggest that indirect genetic effects may not operate uniformly across all types of genetic variation and may depend critically on the developmental timing of genetic effects and their influence on the phenotypic spectrum for traits relevant to partner selection.

Implications and interpretation of our findings of increasing strength of PGI associations with age

One might think that the increasingly strong associations between IQ and PGIs for educational attainment and its cognitive component as children age could be explained by the fact that the underlying SNP associations have been estimated on adult phenotypes which are better correlated with IQ in later childhood than early childhood[28,29]. However, this is an unlikely explanation for several reasons. The common variant genetic correlations between IQ

measured at the different ages and educational attainment measured in adulthood were not significantly different from 1 (Supplementary Table 3), suggesting the increase in PGI associations are most likely due to genome-wide amplification of genetic associations as opposed to different common variants impacting cognitive ability differentially at different ages[32]. Indeed, recent work has shown that observing an increasing PGI association with age while the genetic correlation does not significantly deviate from 1 implies that the common variant heritability increased with age, providing an additional line of evidence supporting the Wilson effect.[33] We observed a positive but not significant association between total SNP heritability of IQ and age (i.e., the Wilson effect) in ALSPAC ($t(1) = 6.93$, $p = 0.0913$, $\beta = 0.0086$, 95% CI = [-0.007, 0.024]; inverse-variance weighted regression) (Supplementary Table 2), consistent with the trend reported in another cohort[30] and recent work showing that the within-family heritability of school grades increased with age in a Norwegian cohort[31]. As a robustness analysis, we repeated all analyses of IQ in ALSPAC with a PGS constructed from another cognitive performance GWAS for Savage et al.[10], which yielded results with identical statistical interpretations to those for PGI_{Cog} (Supplementary Table 13). We did not use results from this PGI in our main analyses since we wanted to have statistically interpretable results when comparing the PGI_{EA} to its cognitive and noncognitive components.

One might posit that the increasing variance explained by PGIs with age could simply be due to a decrease in the environmental effects on IQ (i.e. the heritability could be increasing with age simply because environmental variance decreases), rather than the absolute genetic effect size actually changing. However, we note that since IQ was standardized within age, for the proportion of variance attributed to genetics to increase without a corresponding rise in the absolute genetic effect size, total variance would have to decrease, which contradicts the standardization constraint. The *absolute* genetic contribution must therefore increase with age for the observed effects to hold. Furthermore, we note that in the within-family analyses, the variance in IQ explained by the PGI continues to increase from age 4 to 16. If environmental confounding were driving the pattern, the interaction would attenuate once between-family influences are removed. However, we see no such attenuation of the PGI-by-age interactions after controlling for parental PGIs, suggesting the direct genetic effects are driving the increased genetic contribution with age.

The increasing heritability of IQ with age is well established but its causes remain unclear[32]. In contrast to our results for RVBs, we found evidence across cohorts for increasing *direct* genetic effects of PGIs with age, particularly at the top of the IQ distribution; to our knowledge, this has not been previously demonstrated. Recent work by Morris et al. using longitudinal twin modeling[34] found evidence that the increase in heritability of cognitive performance across development is due to pervasive genetic innovation (i.e., new genetic associations coming into play across time, resulting in genetic correlations between ages that are less than one). In contrast, our molecular genetic results based on PGIs suggest that amplification (i.e., identical genetic associations increasing proportionally with age) is the primary process. However, it is likely that these heterogeneous molecular across the IQ spectrum that we have observed violate the assumptions of the twin model of Morris et al., and may lead to the appearance of genetic innovation where none exists. The increasing direct effect of PGIs for educational

attainment and its cognitive component on IQ may be relevant to the so-called ‘Matthew effect’, the phenomenon whereby individual differences in ability compound over time and increase gaps in cognitive and academic outcomes as children age[35–38]. If this effect is particularly due to associations present in those with high cognitive performance, this could explain the increase in PGI association strength over time that we observe for the upper tail of the phenotype distribution. Several potential mechanisms could drive this, including evocative gene-environment correlations, such as high-performing children being selected into more from cognitively-stimulating environments, or active gene-environment correlations, such as high-performing children being more effective learners and hence improving their cognitive performance further relative to their peers[39].

References

1. Koko, M. *et al.* Exome sequencing of UK birth cohorts. *Wellcome Open Research* (2024).
2. Ramu, A. *et al.* DeNovoGear: de novo indel and point mutation discovery and phasing. *Nat. Methods* **10**, 985–987 (2013).
3. Robinson, J. T. *et al.* Integrative genomics viewer. *Nat. Biotechnol.* **29**, 24–26 (2011).
4. Samocha, K. E. *et al.* A framework for the interpretation of de novo mutation in human disease. *Nat. Genet.* **46**, 944–950 (2014).
5. Yang, J. *et al.* Genetic variance estimation with imputed variants finds negligible missing heritability for human height and body mass index. *Nat. Genet.* **47**, 1114–1120 (2015).
6. Bulik-Sullivan, B. *et al.* An atlas of genetic correlations across human diseases and traits. *Nat. Genet.* **47**, 1236–1241 (2015).
7. Demange, P. A. *et al.* Estimating effects of parents’ cognitive and non-cognitive skills on offspring education using polygenic scores. *Nat. Commun.* **13**, 4801 (2022).
8. Lee, J. J. *et al.* Gene discovery and polygenic prediction from a genome-wide association study of educational attainment in 1.1 million individuals. *Nat. Genet.* **50**, 1112–1121 (2018).
9. DDG2P (Version 3.79). https://panelapp.genomicsengland.co.uk/panels/484/.
10. Savage, J. E. *et al.* Genome-wide association meta-analysis in 269,867 individuals identifies new genetic and functional links to intelligence. *Nat. Genet.* **50**, 912–919 (2018).

11. Pardiñas, A. F. *et al.* Common schizophrenia alleles are enriched in mutation-intolerant genes and in regions under strong background selection. *Nat. Genet.* **50**, 381–389 (2018).

12. Munafò, M. R., Tilling, K., Taylor, A. E., Evans, D. M. & Davey Smith, G. Collider scope: when selection bias can substantially influence observed associations. *Int. J. Epidemiol.* **47**, 226–235 (2018).

13. Hastie, T., Mazumder, R., Lee, J. D. & Zadeh, R. Matrix Completion and Low-Rank SVD via Fast Alternating Least Squares. *J. Mach. Learn. Res.* **16**, 3367–3402 (2015).

14. Benyamin, B. *et al.* Childhood intelligence is heritable, highly polygenic and associated with FNBP1L. *Mol. Psychiatry* **19**, 253–258 (2014).

15. Mollon, J. *et al.* Genetic influence on cognitive development between childhood and adulthood. *Mol. Psychiatry* **26**, 656–665 (2021).

16. Demange, P. A. *et al.* Investigating the genetic architecture of noncognitive skills using GWAS-by-subtraction. *Nat. Genet.* **53**, 35–44 (2021).

17. Satterstrom, F. K. *et al.* Large-Scale Exome Sequencing Study Implicates Both Developmental and Functional Changes in the Neurobiology of Autism. *Cell* **180**, 568–584.e23 (2020).

18. Huang, Q. Q. *et al.* Dissecting the contribution of common variants to risk of rare neurodevelopmental conditions. *bioRxiv* (2024) doi:10.1101/2024.03.05.24303772.

19. Li, M. *et al.* Integrative functional genomic analysis of human brain development and neuropsychiatric risks. *Science* **362**, (2018).

20. Kong, A. *et al.* Rate of de novo mutations and the importance of father's age to disease risk. *Nature* **488**, 471–475 (2012).

21. Jónsson, H. *et al.* Parental influence on human germline de novo mutations in 1,548 trios from Iceland. *Nature* **549**, 519–522 (2017).

22. McElroy, E. *et al.* Feasibility of retrospectively harmonising cognitive measures in five

British birth cohort studies. *UK: CLOSER* (2021).

23. Noghanibehambari, H., Salari, M. & Tavassoli, N. Maternal human capital and infants' health outcomes: Evidence from minimum dropout age policies in the US. *SSM Popul Health* **19**, 101163 (2022).
24. Granés, L., Torà-Rocamora, I., Palacio, M., De la Torre, L. & Llupià, A. Maternal educational level and preterm birth: Exploring inequalities in a hospital-based cohort study. *PLoS One* **18**, e0283901 (2023).
25. Baranowska-Rataj, A., Barclay, K., Costa-Font, J., Myrskylä, M. & Özcan, B. *Preterm Births and Educational Disadvantage: Heterogeneous Effects Across Families and Schools*. (CESifo, Center for Economic Studies & Ifo Institute, 2019).
26. Husby, A., Wohlfahrt, J. & Melbye, M. Gestational age at birth and cognitive outcomes in adolescence: population based full sibling cohort study. *BMJ* **380**, e072779 (2023).
27. Eika, L., Mogstad, M. & Zafar, B. Educational assortative mating and household income inequality. *J. Polit. Econ.* **127**, 2795–2835 (2019).
28. McCall, R. B. Childhood IQ's as Predictors of Adult Educational and Occupational Status. *Science* **197**, 482–483 (1977).
29. Keage, H. A. d. *et al.* Age 7 intelligence and paternal education appear best predictors of educational attainment: The Port Pirie Cohort Study. *Aust. J. Psychol.* **68**, 61–69 (2016).
30. Trzaskowski, M., Yang, J., Visscher, P. M. & Plomin, R. DNA evidence for strong genetic stability and increasing heritability of intelligence from age 7 to 12. *Mol. Psychiatry* **19**, 380–384 (2014).
31. Valstad, M. *et al.* Indirect genetic effects of siblings. *PsyArXiv* (2025) doi:10.31234/osf.io/b4c76_v1.
32. Plomin, R. & Deary, I. J. Genetics and intelligence differences: five special findings. *Mol. Psychiatry* **20**, 98–108 (2015).

33. Durvasula, A. & Price, A. L. Distinct explanations underlie gene-environment interactions in the UK Biobank. *Am. J. Hum. Genet.* **112**, 644–658 (2025).

34. Morris, D. M., Ritchie, S. J. & Plomin, R. Mechanisms of developmental change in genetic and environmental influences on intelligence. *PsyArXiv* (2024) doi:10.31234/osf.io/uqybg.

35. Bast, J. & Reitsma, P. Analyzing the development of individual differences in terms of Matthew effects in reading: results from a Dutch Longitudinal study. *Dev. Psychol.* **34**, 1373–1399 (1998).

36. Shaywitz, B. A. *et al.* A Matthew Effect for IQ but Not for Reading: Results from a Longitudinal Study. *Read. Res. Q.* **30**, 894–906 (1995).

37. Stanovich, K. E. Matthew Effects in Reading: Some Consequences of Individual Differences in the Acquisition of Literacy. *Journal of Education* **189**, 23–55 (2009).

38. Duff, D., Tomblin, J. B. & Catts, H. The Influence of Reading on Vocabulary Growth: A Case for a Matthew Effect. *J. Speech Lang. Hear. Res.* **58**, 853–864 (2015).

39. Tucker-Drob, E. M. & Harden, K. P. Early childhood cognitive development and parental cognitive stimulation: evidence for reciprocal gene-environment transactions. *Dev. Sci.* **15**, 250–259 (2012).

Lay summary and frequently asked questions about the paper

This document was co-developed with the ALSPAC Participant and Public Advisory Panel, particularly Kevin Hebditch and Alistair Byrne.

Part 1 - Lay Summary

Introduction

Our genetic makeup influences many of our traits, from our eye and hair colour to our height and personality. In this study, we looked at how our genetic makeup may affect our *cognitive ability* across early life. Cognitive ability is how well someone solves problems, spots patterns, and uses memory. Cognitive ability influences educational outcomes, and can be used to predict economic and health outcomes later in life.

The aim of our research

We wanted to understand how differences in genetic makeup between children relate to their cognitive ability as they grow, and how this compares with other early-life factors. This knowledge can help explain why some children struggle at school and why some genetic differences increase risk for neurodevelopmental conditions.

What we studied

We analysed data from children aged 4 to 16 years in a UK cohort. We looked at two broad types of genetic differences between people (variants):

1. Rare damaging variants: uncommon changes that are predicted to disrupt how a gene works. (A gene is a section of DNA that encodes a specific protein.)
2. Common variants: millions of small differences spread across the genome. Most don't directly damage genes, but together they can influence how active genes are.

Our results

Our research found that:

- Certain common and rare genetic variants are associated (correlated) with cognitive ability across childhood.
- The association of common variants with cognitive ability increased from early childhood into adolescence.
- The association of rare damaging variants with cognitive ability was strongest in early childhood and decreased with age. This may help explain why some children who carry a rare damaging variant do not develop a neurodevelopmental condition—they may, in part, "grow out of" its early effects.
- Common variants were more important for distinguishing children with high cognitive ability, whereas rare damaging variants were more strongly associated with low cognitive ability.

Genes are one part of a bigger picture. We compared genetic factors with other factors known to affect children's cognitive ability, such as being born early (preterm), the mother's health in pregnancy (for example anaemia, pre-eclampsia or diabetes), and parents' educational qualifications.

- The association between the child's cognitive ability and their common genetic variants was similar in size to the association with their parents' education.
- The association between the child's cognitive ability and their rare damaging variants was similar in size to the association with being born prematurely or with maternal health conditions during pregnancy.

Part 2: Frequently asked questions

Section 1: Introduction to genetics of cognitive ability and neurodevelopmental conditions

This section contains an introduction to general concepts which are relevant to this paper.

What is cognitive ability/IQ?

"Cognitive ability" usually refers to the general capacity to reason, solve problems, think abstractly, comprehend complex ideas, learn efficiently, and otherwise engage in cognitively demanding tasks. Psychologists have developed a variety of tests to try to measure cognitive ability. The usefulness of these tests depends on what one intends to use them for. The most widely-used test developed to measure cognitive ability is the Intelligence Quotient (IQ) test. IQ tests examine various components of cognition including one's ability to complete patterns when shown an incomplete series of images, or identify words that are most similar in meaning to each other from a list of words. A person's IQ (intelligence quotient) is then a summary of how well they performed on these tests.

While the questions asked in an IQ test may not seem relevant to most daily activities, higher IQ is a strong predictor of many life outcomes including the attainment of educational degrees (for example, probability of obtaining an undergraduate degree), lifetime earnings and occupation choice. IQ is also used to help assess whether an individual has intellectual disability, a condition that involves significant impairments in mental functioning.

Cognitive ability is influenced by one's genetics as well as various environmental exposures and events. The best understood environmental factors include brain injuries, premature birth, malnutrition, stroke, a lack of oxygen at birth, and certain medications. The extent to which education, parenting behaviors, and other social factors causally influence cognitive ability (as opposed to just being correlated with them) is an active area of research. We go into more detail on the genetic factors affecting cognitive ability below.

What are direct and indirect genetic effects?

Although people often discuss “nature” (the influence of genetics on traits) versus “nurture” (the influence of environment on traits) as two different things, the relationship between them is complex and separating the two is not straightforward. One reason for this complexity is that a person’s parents and other family members typically provide much of their ‘environment’ (at least in early life), and the behavior of those relatives is partly influenced by their genetics. This brings us to the concept of *direct* versus *indirect* genetic effects, which we explain below.

<u>Direct genetic effects</u>
Direct genetic effects refer to the influence of an individual’s own genes on their traits. For example, if a person has genetic variants that influence their height due to direct genetic effects, these variants contribute to how tall they grow through intrinsic molecular mechanisms. Similarly, certain genetic variants may directly affect personality traits, cognitive abilities, or health outcomes. Direct effects are straightforward in that they arise from how a person’s genetic code influences their own biology and behavior.

<u>Indirect genetic effects</u>
Indirect genetic effects occur when a person’s traits are influenced by the genetics of others in their environment, such as their parents or siblings. These effects are sometimes called “genetic nurture”. Genetic nurture can arise, for example, through social or environmental interactions. For instance, a parent’s genes might influence how nurturing or structured their parenting style is, which in turn affects the child’s development. In this case, the parent’s genes are indirectly shaping the child’s traits. Similarly, a sibling’s genetic predisposition for sociability might create a more stimulating social environment, indirectly influencing another sibling’s social skills. Genetic nurture can also manifest through the prenatal environment - a mother’s genes influence aspects of the environment that a baby experiences *in utero*, which could influence later-life traits.

This nuanced perspective of “nature” and “nurture” provides a more accurate picture of human development and individual differences.

What are neurodevelopmental conditions and what causes them?

Neurodevelopmental conditions are conditions that affect the growth and development of the brain and nervous system. These conditions can lead to various outcomes including developmental delay, intellectual disability, and seizures. People with neurodevelopmental conditions typically require special care so it is important that these conditions are identified early in an individual’s life. These conditions are frequently due to genetic causes, but this is not always the case. Finding the cause for these conditions can help doctors determine the best treatment options and helps parents understand the cause for these conditions for future family planning. Genetic studies that study neurodevelopment in “neurotypical” individuals without such conditions, such as our current study, can help scientists understand these conditions better.

Scientists have found that at least 50% of people with rare neurodevelopmental conditions have one or two rare genetic variants in a single gene causing their condition. Most commonly, these are new genetic variants that arise in the egg or sperm (called *de novo* mutations) and which affect just one of the two copies of a gene. Sometimes, the conditions can be caused by having two different genetic variants in the same gene, each inherited from a different parent. However, common and rare inherited genetic variants distributed across many genes also predispose to neurodevelopmental conditions.

Section 2: Background to the study

What was the motivation for this study?

A key observation that motivated this study is that sometimes rare genetic variants can cause neurodevelopmental conditions in only a subset of the individuals who carry them. This phenomenon is called "incomplete penetrance". Some rare variants can be incompletely penetrant within a family - they may be inherited by an affected child from a parent who appears to be unaffected. One particular class of incompletely penetrant variants explain about 10% of children with neurodevelopmental conditions. We suspected that at least some of these variants might actually have larger effects in childhood than adulthood, so the parent carrying the variant may have had cognitive difficulties when they were a child but then 'grown out of them'. Thus, a major motivation for this study was to study the effects of these rare variants in a set of people whose cognitive ability had been measured repeatedly, in order to test whether these effects were changing as people aged.

Who conducted this study?

This study was conducted by researchers at the Wellcome Sanger Institute. The data used in the research was collected through three major cohort studies: the Avon Longitudinal Study of Parents and Children (ALSPAC), the UK Millennium Cohort Study (MCS), and the UK Biobank (UKB), described further below.

What were their key research questions?

The researchers addressed several questions in this study:

- Do rare genetic variants that predispose to neurodevelopmental conditions influence cognitive ability in the general population in early childhood, and do their genetic effects change as children age?
- What about the effects of common variants on childhood cognitive ability - how do these compare to the effects of rare variants, and do their effects change with age?
- Do different types of genetic variants have a greater influence on high versus low cognitive ability?
- How do these genetic influences compare to the influences of environmental exposures such as premature birth?

What was already known about the genetics of cognitive ability?

There are several well-replicated results regarding the genetics of cognitive ability that can be summarized as follows:

1. The influence of genetic variation on cognitive ability is substantial regardless of how it is measured.
2. The total influence of genetic variation on cognitive ability increases from childhood to early adulthood and then remains largely stable across adulthood.
3. The genetic variants influencing cognitive ability primarily act by influencing neurodevelopment and brain function and also modulate risk for neurodevelopmental, neurological, and psychiatric conditions.

Genetic studies using various study designs (e.g., looking at similarities between family members) have consistently shown that cognitive ability, as measured by IQ and related tests, is appreciably influenced by one's genes. However, this influence is significantly lower in childhood (when 20-30% of the variation in cognitive ability is due to variation in genes), and gradually increases from early childhood to adulthood. Once established in early adulthood, it remains relatively stable throughout later life. This phenomenon is one of the most well-replicated findings in psychology, confirmed across different time periods and geographic locations.

The paper talks about the "population model" and the "trio model" - what are these?

The population model looks at the correlation between someone's genetic variants and their own traits, such as cognitive ability - we call these "*population effects*". However, it doesn't separate the direct genetic effects of those variants (described above) from other influences that are correlated with those variants. For example, it can't distinguish between the direct impact of a person's variants on their cognitive ability and the effects of those same genetic variants in their parents, which may have influenced parental behaviours and hence the environment in which they grew up (a concept called "genetic nurture", described above). As a result, the population model doesn't give a clear, causal estimate of how a person's own genes affect their cognitive ability.

The trio model, on the other hand, uses information about the genetics of both parents to remove nearly all sources of confounding, including genetic nurture. By controlling for the parents' genetics, the trio model isolates the *direct effects* of a person's genes on their cognitive ability, providing a more accurate, causal estimate.

What data were used in this study and how/why were they collected?

This study used data from three major sources: the Avon Longitudinal Study of Parents and Children (ALSPAC), the Millennium Cohort Study (MCS), and the UK Biobank.

ALSPAC

This is a long-term study based in the Bristol area of the UK. It began in the early 1990s by recruiting over 14,000 pregnant women and has followed their children through childhood, adolescence, and beyond. Researchers collected data at multiple time points, tracking changes in cognitive performance, health, and environment. This type of sampling is called longitudinal sampling, where the same individuals are studied repeatedly over time to observe how they change and develop. We specifically considered measurements of cognitive performance (IQ) from ages 4, 8, and 16 and school grades from ages 11 and 14.

MCS

This is another long-term UK study, but it focuses on children born around 2000-2002. It includes families from across the UK and is designed to represent the diversity of the population. Like ALSPAC, MCS follows children as they grow, collecting data on their health, education, and other factors at regular intervals.

UK Biobank

This is a large resource of genetic and health data from adults in the UK. While it doesn't follow individuals from birth, it provides a wealth of genetic information to compare with the findings from ALSPAC and MCS.

With this combination of datasets, the study was able to observe how genetic and environmental factors influence cognitive performance at different ages and also how these early influences carry through to later life.

Section 3: Study design and results

What are polygenic indices and rare variant burden scores, and how were they used in this study?

A polygenic index for a trait is a value that sums up the effects of common genetic variants (e.g. those present in more than 1% of the population) on the trait. This value can be thought of as the average trait value we would expect among a group of people with the same polygenic index. For example, a polygenic index for height is the predicted value for a person's height given their common genetic variants. Polygenic indices are sometimes called "polygenic scores".

Rare variant burden scores also sum up the effects of variants across the genome (in this case, rare variants present in e.g. less than 0.1% of the population), but are different from the polygenic indices in that they are not created to predict any specific trait. Instead, the rare variant burden score is a measure of the total number of rare genetic variants a person has that damage the function of their genes. However, instead of each variant contributing equally to this score, damaging variants in some genes count more than those in other genes. Specifically, this score up-weights damaging variants in genes in which such variants have been removed from the population by natural selection throughout human history ("constrained genes").

In this study, we wanted to see how well rare variant burden scores and polygenic indices for various traits predict cognitive ability in children across development. We were specifically interested in understanding whether they predict cognitive ability better or worse at different ages.

How useful are these genetic scores for predicting cognitive ability?

Polygenic indices are meaningfully associated with cognitive ability - the most predictive one explained between 10-15% of the variation in IQ between individuals. However, they are very poor at predicting cognitive ability at the individual level. Many people with polygenic indices that predict low cognitive ability turn out to perform well on the cognitive ability tests and vice versa. We are only able to say that, across many individuals with similar polygenic indices, the polygenic indices can predict their average cognitive ability with some degree of accuracy, and that there is a lot of variability in what any one of these individuals' cognitive ability is.

For example, the plot below shows the measured IQ for a few hundred people on the vertical axis, and the IQ scores predicted from their polygenic indices on the horizontal axis. The red line indicates their average relationship. While a correlation is clearly present, there remains a lot of uncertainty in any one individual's IQ given the IQ predicted from their polygenic index.

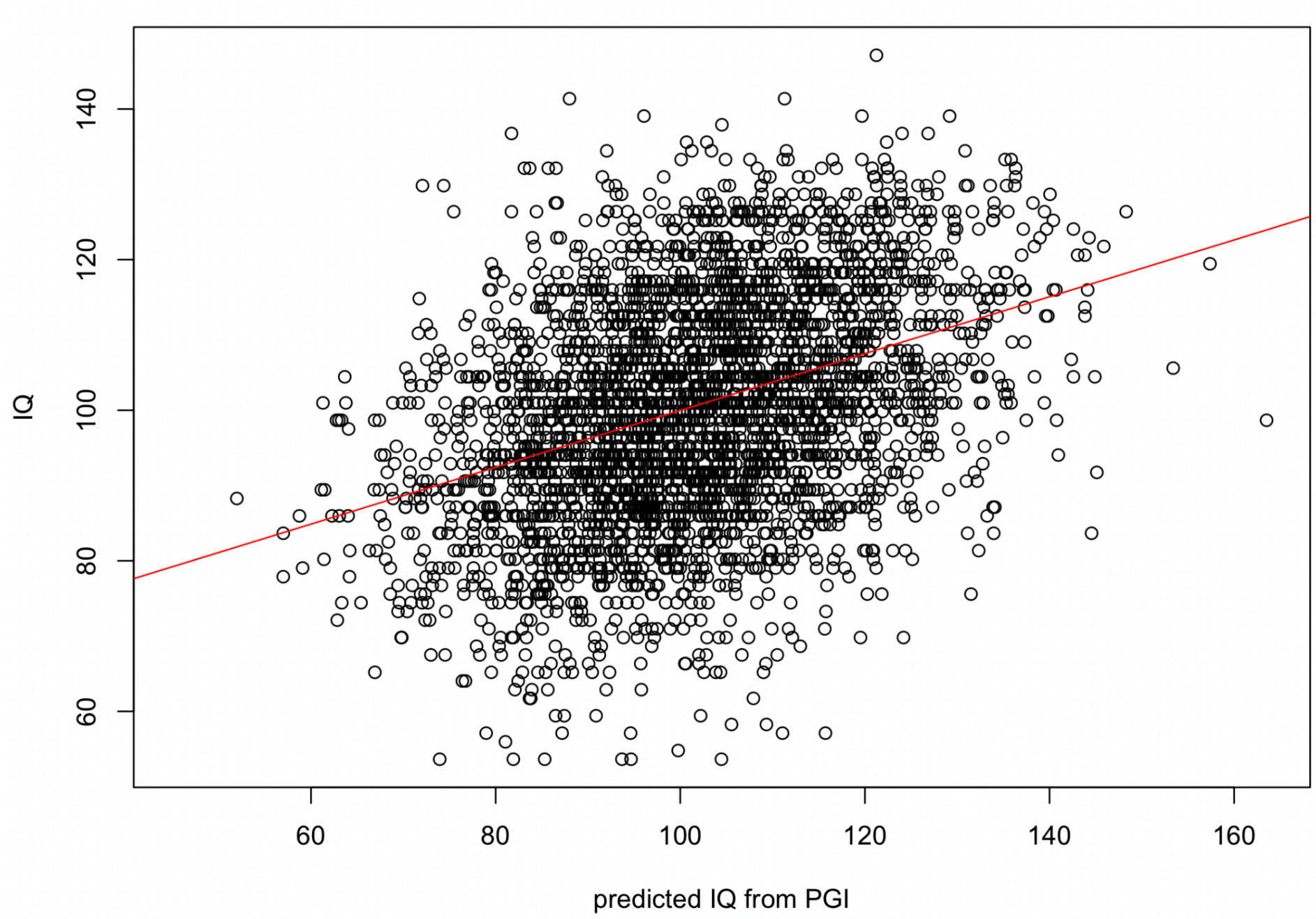

Similarly, rare variant burden does not perfectly predict IQ, as indicated by the plot below. For example, among the children in the top fifth percentile of rare variant burden (the children with the highest rare variant burden relative to their peers), 7% have IQs below 70 at age 8, whereas among the other children the rate is around 3%.

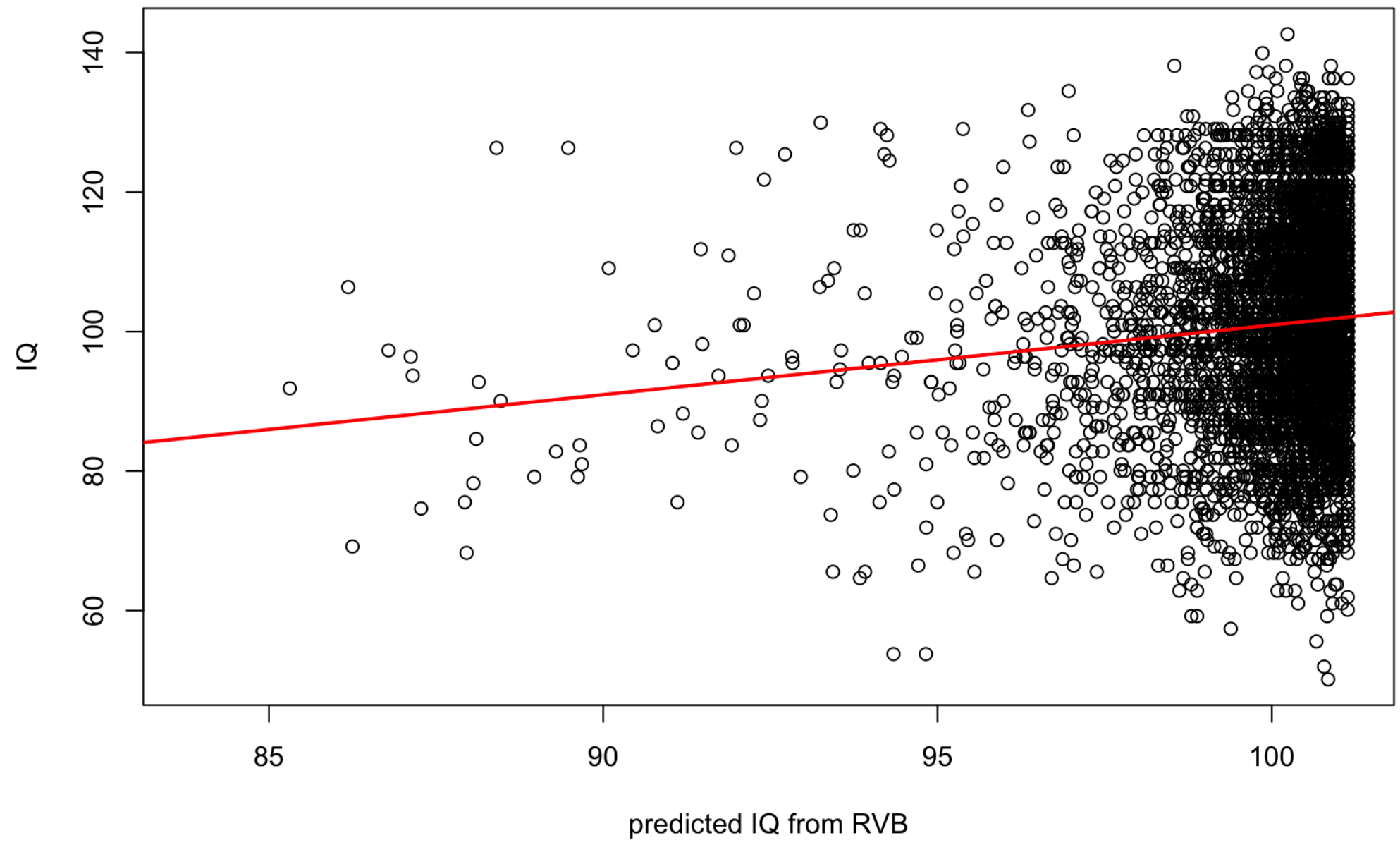

What did you learn about the contribution of common genetic variants to cognitive ability across childhood and adolescence?

We found that the contribution of common genetic variants (as measured by polygenic indices for cognitive ability and related traits) increases with age, with the percent of the variance explained by the polygenic indices increasing from 11% at age 4 to 14% at age 16. This finding suggests that the influence of genetic effects captured by the polygenic indices become more important for cognitive ability with age. Importantly, we found that this is true for both the population and direct effect (explained above) - that this is due to causal effects of these genetic variants.

We also found that the influence of common variants was stronger at the higher end of the cognitive ability distribution, meaning that individuals with very high cognitive ability tended to have particularly high polygenic scores. This suggests that the cumulative effect of common genetic variants plays a larger role in distinguishing individuals at the upper extreme of cognitive ability.

What did you learn about the contribution of rare genetic variants?

The rare variant burden scores were associated with lower cognitive ability at all ages but only explained a small amount of variance (less than 3% of the variation in IQ between individuals at any given age). In contrast to the effects of common variants captured by polygenic indices (described above), the influence of these rare variant burden scores decreases with age. This finding suggests that the rare variants have a stronger effect on childhood cognitive ability in

early childhood that gets weaker as they age. This is potentially due to biological changes in the brain that "make up" for the negative impact of these variants, or because children with these variants are identified early in life and some intervention they experience (for example, additional support in school) counteracts the effect of the damaging rare variants. As for the common variants, we observed that this changing genetic effect with time is due to direct genetic effects, suggesting it is a causal association between the child's rare variants and their cognitive ability.

We found that the association between rare damaging variants and lower cognitive ability was more pronounced at the lower end of the cognitive ability distribution. That is, individuals with the lowest cognitive ability scores were disproportionately affected by rare damaging genetic variants. This aligns with previous research suggesting that rare, deleterious variants contribute significantly to neurodevelopmental conditions and intellectual disability.

What did you learn about the contribution of other factors to childhood cognitive ability?

The study found that parental education, premature birth, and maternal health during pregnancy are all correlated with childhood cognitive ability. Parental education emerged as a particularly strong likely influence, with children of more highly educated parents tending to perform better on cognitive tests. This association remained consistent throughout childhood and adolescence. Parental education explained a similar amount of variation to the direct genetic effects of the children's polygenic indices on their cognitive performance. Being born prematurely and maternal illness during pregnancy were associated with lower cognitive abilities early in life. However, the impact of these factors lessened as children grew older, suggesting that developmental processes or supportive environments may help mitigate these early disadvantages.

The study was unable to demonstrate the extent to which parental education and these prenatal factors actually *causally* influence children's cognitive ability - it was only able to look at correlations. However, previous work with other study designs has shown that they do have causal influences on children's neurodevelopmental outcomes. This study showed that, in aggregate, about 25% of the variation in children's IQ at age 16 is explained by parental education, these prenatal factors, and the parents' and children's genetic scores. Although parental education and prenatal factors are often considered "environmental factors", they are not strictly independent of genetics. The parents' educational attainment is influenced by their genetics, and the prenatal factors are also likely to be influenced by the mother's genetics to some extent.

What are the limitations of your study?

The study had several limitations. First, the main findings about how genetic effects change over time were based on data from a single cohort (ALSPAC), which measured IQ at only three points in childhood and adolescence. While the results were supported by findings from two

other cohorts (Millenium Cohort Study and UK Biobank), the limited number of time points and reliance on one primary cohort could affect the generalizability of the findings.

Additionally, the genetic data used in the study only partially captures the total influence of genetic variants. This is because the study was limited by affordable technologies, so it only considered common genetic variants plus rare genetic variants in parts of the genome that encode genes, which make up only ~1% of the entire genome. It is likely that rare genetic variants across the rest of the genome also have an important influence on cognitive ability, and so far we know very little about these.

Finally, there is the potential for biases due to missing data and attrition (participants dropping out of the study) over time, which are common in long-term studies like ALSPAC and MCS. Despite attempts to address these issues through statistical methods, such biases may still impact the results.

Section 4: Implications of the findings

Could people be identified from these findings?

No, people cannot be identified from these findings. While the study does use individual-level genetic data, all of this information is anonymized, meaning that researchers cannot link the data back to any specific person. Additionally, the paper only presents results in a summarized or grouped form, so no individual-level data is shared or made public. Strict privacy measures are in place to ensure that the data is stored securely, and only authorized researchers can access it under strict ethical guidelines. This ensures that everyone’s privacy is fully protected throughout the study.

My child has a neurodevelopmental condition. What do the findings of this study mean for my child and our family?

At this stage, we don’t know enough to answer this question. The results from this study hint that certain developmental delays may resolve or lessen over time. However, this research was conducted in a relatively healthy group of children, not in children with diagnosed genetic neurodevelopmental conditions, particularly those with more complex or severe needs. As such, we cannot assume these findings apply to all children, especially those with significant intellectual or developmental disabilities. More research is needed in cohorts of families with neurodevelopmental conditions to confirm whether this finding is relevant for them. Those future studies should try to explore whether genetic variants in particular genes influence how children’s learning abilities change as they grow older, and the factors that modify these effects.

Why does this study focus on individuals with White British ancestry ?

In this study, we focus on individuals of White British ancestry to ensure that our genetic analyses are as accurate and meaningful as possible. This decision is based on key scientific considerations:

1. **Avoiding bias from genetic and environmental differences between populations**: Genetic studies rely on statistical models that link genetic variants to traits, but these models assume that genetic and environmental influences are independent. In reality, systematic differences in environment (such as access to education, healthcare, or socioeconomic conditions) often correlate with genetic differences between ancestry groups simply due to historical population structure. If we combined multiple ancestry groups in our analysis, we could accidentally attribute differences in cognitive ability to genetic factors when they are actually due to environmental disparities. By focusing on a single ancestry group, we reduce the risk of these biases and make our genetic findings more interpretable.

2. **Polygenic Indices were developed using European Samples**: Our analysis includes polygenic indices (PGIs), which summarize the combined effects of many common genetic variants on cognitive ability. These PGIs were originally developed using large studies conducted in European ancestry populations. Because the frequencies and effect sizes of genetic variants can vary across ancestry groups, PGIs "trained" in one group tend to perform poorly in others. Applying a PGI trained in Europeans to individuals of non-European ancestry could lead to misleading or inaccurate predictions, making it difficult to draw meaningful conclusions.

3. **Future research and the need for diverse studies**: While this study focuses on White British individuals, the underlying biological mechanisms of cognitive ability are expected to be broadly similar across human populations. However, because specific genetic variants and their effects may differ, future research will be needed to determine whether these findings generalize to other ancestry groups. Increasing the diversity of genetic studies is a major goal for the field, as it will help ensure that research benefits all populations equitably.

By restricting our study to a single ancestry group, we improve the reliability of our findings. However, we acknowledge the importance of expanding genetic research to include more diverse populations in future studies.

Could these findings or the data used in this study be misused?

Like any research, these findings could theoretically be misused, or the data could be misused if it fell into the wrong hands - for example, by those seeking to justify discrimination or make unfounded claims that misrepresent the results. However, several factors make such misuse difficult and illegal.

- **Legal safeguards:** In the UK, the Equality Act 2010 prohibits discrimination on "protected characteristics" including genetics, and the Human Tissue Act 2004 regulates the collection, storage and use of human DNA; all research using identifiable samples must have licences and participant consent. EU & UK GDPR (Data Protection

Act 2018) requires explicit consent for processing people's genetic data, as well as mandating secure storage and restricting sharing. Breaches of these can incur large fines.

- Ethical review and data governance: Research ethics committees oversee all genetic studies, ensuring protocols specify how data are collected, stored and shared, and how participants' rights are protected. Genetic and health data in ALSPAC and other research cohorts are anonymised before being shared with researchers, and must be held on secure computer servers; only accredited researchers gain access, via a strict Data Access Committee process.
- **Professional codes of conduct:** The research community actively monitors for misuse of genetic findings and has strong professional standards against using genetics for discriminatory purposes. For example, international standards such as the Declaration of Helsinki and the Human Genome Organisation's (HUGO) Statement on the Ethical, Legal and Social Implications of Genomics forbid using genetics to stigmatize or disenfranchise groups.
- **Scientific limitations:** Importantly, this study only examined White British ancestry individuals and it provides no basis for comparison to other groups - any claims about group differences based on this work would be scientifically baseless. Furthermore, the effect sizes of genetic scores used in this study explain only a small fraction of individual variation and cannot predict outcomes deterministically—environmental, educational and social factors remain critically important.

While vigilance is always needed, these legal, practical, and scientific barriers provide substantial protection against those who might seek to misuse genetic research for harmful ends.

How might the results of this study or the information in this document be misinterpreted and why are these interpretations incorrect?

The results of this study, like many in genetics and developmental science, could be misinterpreted in ways that oversimplify or exaggerate their implications. One common misunderstanding might be the idea that the findings have clear, actionable implications for any one individual. However, this study focuses on group-level patterns and developmental trajectories, which provide insights into populations, not specific predictions for an individual child. Cognitive development is influenced by a complex interplay of genetic and environmental factors, and the results should not be used to make deterministic claims about any one person's future outcomes.

Another potential misinterpretation could involve drawing conclusions about differences between ethnic groups. Such an interpretation would be entirely misguided. The study does not address or provide evidence for such differences, since it focuses only on people who are inferred to have white British ancestry based on their genetics. Thus, it says nothing about any genetic or

cognitive disparities between groups. Misusing the findings to support unfounded claims about group differences ignores the limitations of the research and the broader context in which cognitive traits develop.

Ultimately, the study emphasizes developmental mechanisms and trajectories rather than fixed outcomes or group comparisons. Misinterpretations arise when findings are taken out of this context or used to make oversimplified claims that the data do not support. It's essential to approach the results with an understanding of their nuance and limitations, recognizing that they are a step toward understanding complex processes, not definitive answers about individuals or groups.

Core concepts and terminology used above

What are rare neurodevelopmental conditions?

These are a group of conditions that affect the growth and development of the brain and are first noticed during childhood. Common features of these conditions include intellectual disability, delays in achieving developmental milestones such as walking and talking, seizures and a small head size. Children with these conditions often need educational, health and social support throughout their lives. Cumulatively, rare neurodevelopmental conditions affect ~1% of newborns, but this includes many individually much rarer conditions.

What is a genetic "variant"?

We can think of our DNA as a long sequence of letters, and genetic variants as changes in those letters. Everyone has millions of genetic variants in their DNA, and these are partly what makes people different from one another. These changes can involve just a single letter, which may be changed, deleted or added. They can also involve large sections of DNA being deleted, duplicated or rearranged.

What are some different types of genetic variants, and which genetic variants are considered in this study?

Some genetic variants are commonly seen in the general population - "common variants" - while some are rarely seen in the general population - "rare variants". A variant that is seen in more than 1% of people is usually considered to be "common", and one that is seen in less than 1% of people is usually considered "rare".

This study considers two types of genetic variants. The first includes common single-letter changes across the whole genome ("single nucleotide polymorphisms", or SNPs). The second includes rare variants that change the sequence of proteins encoded by genes - these are referred to as "rare coding variants" or "rare damaging variants".

What do you mean by “phenotype distribution”?

The phenotype distribution refers to the range (i.e. maximum and minimum) and frequency of observed values for a particular trait (“phenotype”) within a population (i.e. how many of each value is observed). In this study, the phenotype of interest is cognitive ability, which varies across individuals, so we talk about the “cognitive ability distribution”. Some people score very high, some very low, and most fall somewhere in between, forming a distribution that can often be visualized as a bell curve (normal distribution, see here for a further explanation of the distribution of IQ). Studying the entire distribution allows researchers to understand how genetic and environmental influences contribute to differences in cognitive ability across individuals, rather than just focusing on an "average" effect.

Glossary

DNA – The molecule that carries genetic information in all living organisms. DNA is made up of sequences of four chemical bases (A, T, C, and G) that encode instructions for building and maintaining the body.

Genome – The complete set of DNA in an organism, including all of its genes. The human genome contains about 20,000 genes and all the genetic information needed for development and function.

Gene – A section of DNA that contains instructions for building proteins, which carry out many essential functions in the body. Genes influence traits such as height, eye color, and cognitive ability. The human genome contains about 20,000 genes.

Genetic variant – A difference in the DNA sequence between individuals. Some variants are common in the population, while others are rare. These differences can influence traits or have no noticeable effect at all.

Single nucleotide polymorphism (SNP) – A type of genetic variant that involves a change in a single "letter" of DNA. SNPs are the most common type of genetic variation in humans and can contribute to differences in traits like cognitive ability, personality, and health.

Polygenic index (PGI) – A number that summarizes the combined effect of many common genetic variants (SNPs) on a particular trait. For example, a PGI for cognitive ability estimates the genetic predisposition for higher or lower cognitive ability based on many small genetic differences.

Rare variant burden – A measure of how many rare, damaging genetic variants a person carries in important genes. A higher rare variant burden means a person has more rare genetic changes that could negatively affect neurodevelopment.

Distribution – The range of values that a trait can take in a population. For example, cognitive ability varies between individuals, with most people falling near the average and fewer people at the very high or very low extremes.

Neurodevelopmental condition – A condition that affects the growth and development of the brain and nervous system. These conditions can lead to cognitive, motor, or social impairments. Examples include autism, intellectual disability, and certain forms of epilepsy. People with neurodevelopmental conditions often require specialized support in education, healthcare, or daily life.

Incomplete penetrance – A situation where a genetic variant that causes a condition in some people does not cause it in others. This means someone can carry a genetic variant linked to a neurodevelopmental condition but still develop typically. The reasons for incomplete penetrance are not always clear but may involve other genetic factors or environmental influences.

Trio model – A genetic study design that analyzes DNA from both parents and their child to isolate direct genetic effects. By comparing the child's genetic variants to those of their parents, researchers can separate the child's own genetic influences from indirect effects coming from the parents' genetics (such as genetic nurture).

Direct genetic effect – The influence of a person's own genetic variants on their traits. For example, if someone's genes contribute to their cognitive ability by directly affecting brain development, that is a direct genetic effect.

Indirect genetic effect (genetic nurture) – When a person's traits are influenced by the genetics of others in their environment, such as their parents. For example, a parent's genetic predisposition to enjoy reading might lead them to create a more enriching learning environment for their child, indirectly boosting the child's cognitive ability.
