# Supplementary material for: Common and rare genetic variant associations with cognitive performance across development in British birth cohorts
Source: Nat Hum Behav. 2026 Jul 10;10(9):1841–54. doi: 10.1038/s41562-026-02491-8 (PMC13590407; doi:10.1038/s41562-026-02491-8)
Supplement: Supplementary file 2 [file 41562_2026_2491_MOESM2_ESM.pdf]

nature portfolio

Corresponding author(s): Daniel Malawsky and Hilary Martin

Last updated by author(s): Mar 7, 2026

Reporting Summary

Nature Portfolio wishes to improve the reproducibility of the work that we publish. This form provides structure for consistency and transparency in reporting. For further information on Nature Portfolio policies, see our Editorial Policies and the Editorial Policy Checklist.

Statistics

For all statistical analyses, confirm that the following items are present in the figure legend, table legend, main text, or Methods section.

n/a	Confirmed	
☐	☒	The exact sample size (n) for each experimental group/condition, given as a discrete number and unit of measurement
☐	☒	A statement on whether measurements were taken from distinct samples or whether the same sample was measured repeatedly
☐	☒	The statistical test(s) used AND whether they are one- or two-sided<br>*Only common tests should be described solely by name; describe more complex techniques in the Methods section.*
☐	☒	A description of all covariates tested
☐	☒	A description of any assumptions or corrections, such as tests of normality and adjustment for multiple comparisons
☐	☒	A full description of the statistical parameters including central tendency (e.g. means) or other basic estimates (e.g. regression coefficient) AND variation (e.g. standard deviation) or associated estimates of uncertainty (e.g. confidence intervals)
☐	☒	For null hypothesis testing, the test statistic (e.g. F, t, r) with confidence intervals, effect sizes, degrees of freedom and P value noted<br>*Give P values as exact values whenever suitable.*
☒	☐	For Bayesian analysis, information on the choice of priors and Markov chain Monte Carlo settings
☐	☒	For hierarchical and complex designs, identification of the appropriate level for tests and full reporting of outcomes
☐	☒	Estimates of effect sizes (e.g. Cohen's d, Pearson's r), indicating how they were calculated

Our web collection on statistics for biologists contains articles on many of the points above.

Software and code

Policy information about availability of computer code

Data collection

GATK v4.2 was used to call variants (SNVs and indels) from whole exome sequencing data. Quality control of variants involved bcftools trio-dnm2 for detecting Mendelian inheritance violations, and a random forest classifier implemented in Python for filtering putative de novo mutations.

Data analysis

Genetic analyses and polygenic score calculations were conducted using PLINK v1.9, while relatedness inference was performed with KING v2.3.1 and population structure analysis with EIGENSOFT v7.2.1. Statistical analyses were conducted in R v4.6.0 statistical software, utilizing the lme4 v2.0-1 package for mixed effects modeling, quantreg v6.1 and rqpd v0.6 packages for quantile regression, and factanal v3.6.2 for factor analysis. Polygenic score weights were generated using LDpred2-auto v1.12.20, and Mendelian imputation v0.0.23 of parental genotypes was performed using snipar. Missing IQ values were imputed using SoftImpute v1.4-3. Heritability and genetic correlation analyses were conducted using GCTA v1.95.1 and LDSC v1.0.1.

For manuscripts utilizing custom algorithms or software that are central to the research but not yet described in published literature, software must be made available to editors and reviewers. We strongly encourage code deposition in a community repository (e.g. GitHub). See the Nature Portfolio guidelines for submitting code & software for further information.

Data

Policy information about availability of data

All manuscripts must include a data availability statement. This statement should provide the following information, where applicable:

- Accession codes, unique identifiers, or web links for publicly available datasets
- A description of any restrictions on data availability
- For clinical datasets or third party data, please ensure that the statement adheres to our policy

Researchers can apply to access data from ALSPAC (https://www.bristol.ac.uk/alspac/researchers/access/), MCS (https://cls.ucl.ac.uk/dataaccess-training/data-access/), and UK Biobank (https://www.ukbiobank.ac.uk/enable-your-research/apply-for-access).

Research involving human participants, their data, or biological material

Policy information about studies with human participants or human data. See also policy information about sex, gender (identity/presentation), and sexual orientation and race, ethnicity and racism.

Reporting on sex and gender	Sex was determined genetically using chromosomal variants detected in genotype microarray data. Individuals were classified as having either XX or XY chromosomes. Sex was included as a covariate in all regression analyses. All analyses were performed in a non sex-specific manner.
Reporting on race, ethnicity, or other socially relevant groupings	Analyses were restricted to individuals whose genetic ancestry clustered with European reference samples from the 1000 Genomes Project, as determined through principal component analysis of genome-wide genetic variation. This analytical choice limits the generalizability of our findings to populations of European genetic ancestry.
Population characteristics	We utilized three population-based cohorts from the UK, recruited between 1990 and 2011, encompassing ages 0–69 at enrollment. The Avon Longitudinal Study of Parents and Children (ALSPAC) cohort was recruited in the Bristol area (formerly Avon county) during the early 1990s, targeting women in early pregnancy. The Millennium Cohort Study (MCS) was recruited across the entire UK in the early 2000s, similarly focusing on early pregnancy. The UK Biobank (UKB) cohort consisted of adults aged 40–69 years, recruited nationwide between 2006 and 2011.
Recruitment	The ALSPAC cohort was recruited in the early 1990s from pregnant women in the Bristol area, potentially introducing regional bias. The MCS cohort and ALSPAC cohort recruited mothers in hospitals/clinics, which may lead to bias in under-representing mothers that did not attend these visits. The UK Biobank cohort recruited adults aged 40–69 between 2006 and 2011, with participants self-selecting into the study, potentially introducing healthy volunteer bias and under-representing younger and more socioeconomically deprived individuals.
Ethics oversight	Cambridge South Research Ethics Committee<br>Republic of Ireland Research Ethics Committee<br>East of England-Cambridge Central Research Ethics Committee<br>North West Multi-centre Research Ethics Committee (MREC)

Note that full information on the approval of the study protocol must also be provided in the manuscript.

Field-specific reporting

Please select the one below that is the best fit for your research. If you are not sure, read the appropriate sections before making your selection.

☒ Life sciences ☐ Behavioural & social sciences ☐ Ecological, evolutionary & environmental sciences

For a reference copy of the document with all sections, see nature.com/documents/nr-reporting-summary-flat.pdf

Life sciences study design

All studies must disclose on these points even when the disclosure is negative.

Sample size	Sample sizes were determined at the inceptions of the cohort to ensure statistical power to identify genetic influences on human traits and diseases. We used the maximal cohort size possible for each analysis from a given cohort.
Data exclusions	Restrictions based on genetic ancestry and sex determination were done as described above. No further data exclusions were conducted.
Replication	We replicated results directly or indirectly by using ALSPAC as our discovery cohort and MCS and UK Biobank as replication cohorts. Results were highly concordant across cohorts, and we highlight throughout the manuscript the extent of replication for each analysis. We controlled for sex, age, and genetic PCs throughout.
Randomization	No experimental groups were assigned.
Blinding	No experimental groups were assigned.

Reporting for specific materials, systems and methods

We require information from authors about some types of materials, experimental systems and methods used in many studies. Here, indicate whether each material, system or method listed is relevant to your study. If you are not sure if a list item applies to your research, read the appropriate section before selecting a response.

Materials & experimental systems

n/a	Involved in the study
☒	☐ Antibodies
☒	☐ Eukaryotic cell lines
☒	☐ Palaeontology and archaeology
☒	☐ Animals and other organisms
☒	☐ Clinical data
☒	☐ Dual use research of concern
☒	☐ Plants

Methods

n/a	Involved in the study
☒	☐ ChIP-seq
☒	☐ Flow cytometry
☒	☐ MRI-based neuroimaging

Plants

Seed stocks	*Report on the source of all seed stocks or other plant material used. If applicable, state the seed stock centre and catalogue number. If plant specimens were collected from the field, describe the collection location, date and sampling procedures.*
Novel plant genotypes	*Describe the methods by which all novel plant genotypes were produced. This includes those generated by transgenic approaches, gene editing, chemical/radiation-based mutagenesis and hybridization. For transgenic lines, describe the transformation method, the number of independent lines analyzed and the generation upon which experiments were performed. For gene-edited lines, describe the editor used, the endogenous sequence targeted for editing, the targeting guide RNA sequence (if applicable) and how the editor was applied.*
Authentication	*Describe any authentication procedures for each seed stock used or novel genotype generated. Describe any experiments used to assess the effect of a mutation and, where applicable, how potential secondary effects (e.g. second site T-DNA insertions, mosiacism, off-target gene editing) were examined.*
