# Supplementary material for: Common and rare genetic variant associations with cognitive performance across development in British birth cohorts
Source: Nat Hum Behav. 2026 Jul 10;10(9):1841–54. doi: 10.1038/s41562-026-02491-8 (PMC13590407; doi:10.1038/s41562-026-02491-8)
Supplement: Supplementary file 3 [file 41562_2026_2491_MOESM3_ESM.pdf]

nature portfolio

Peer Review File

Common and rare genetic variant associations with cognitive performance across development in British birth cohorts

Corresponding Author: Dr Daniel Malawsky

Version 0:

Decision Letter:

11th March 2025

Dear Mr Malawsky,

Thank you once again for your manuscript, entitled "The differential effects of common and rare genetic variants on cognitive performance across development", and for your patience during the peer review process.

Your Article has now been evaluated by 3 referees. You will see from their comments copied below that, although they find your work of potential interest, they have raised quite substantial concerns. In light of these comments, we cannot accept the manuscript for publication, but would be interested in considering a revised version if you are willing and able to fully address reviewer and editorial concerns.

We hope you will find the referees' comments useful as you decide how to proceed. If you wish to submit a substantially revised manuscript, please bear in mind that we will be reluctant to approach the referees again in the absence of major revisions. We are committed to providing a fair and constructive peer-review process. Do not hesitate to contact us if there are specific requests from the reviewers that you believe are technically impossible or unlikely to yield a meaningful outcome.

To guide the scope of the revisions, the editors discuss the referee reports in detail within the team, including with the chief editor, with a view to (1) identifying key priorities that should be addressed in revision and (2) overruling referee requests that are deemed beyond the scope of the current study. We hope that you will find the prioritised set of referee points to be useful when revising your study. Please do not hesitate to get in touch if you would like to discuss these issues further.

Specifically, please address the following:

- Editorially we are concerned that you use causal language throughout your work, and discuss policy implications, yet report only correlational findings, and this concern is also emphasised by both Reviewer #1 and Reviewer #3. Please remove all causal language, and please also remove all policy implications that stem from a causal interpretation of your results (e.g. genetic screening), since these do not apply here. When interpreting your findings, please be clear that you can speak to association only and not causality. Please also note this in the Limitations section of your Discussion
- Reviewer #1 raised major concerns regarding the effect of attrition on your results, as well as insufficient information on your imputation methods. Please clarify these issues explicitly as suggested by Reviewer #1
- Reviewer #1 also pointed out potential issues with heteroscedasticity. Please respond to these comments by clarifying how you accounted for heteroscedasticity in your primary analyses, or conduct relevant analyses as robustness check if you have not done so.
- Reviewer #1 and #3 have raised further concerns on your analytical approach and interpretation of results, such as underpowered interaction (Reviewer #1) and inadequate comparison of effect patterns between PGI-based associations and RVB scores. Please respond to these comments and revise your manuscript appropriately.

Finally, your revised manuscript must comply fully with our editorial policies and formatting requirements. Failure to do so will result in your manuscript being returned to you, which will delay its consideration. To assist you in this process, I have attached a checklist that lists all of our requirements. If you have any questions about any of our policies or formatting, please don't hesitate to contact me.

If you wish to submit a suitably revised manuscript, we would hope to receive it within 4 months. I would be grateful if you could contact us as soon as possible if you foresee difficulties with meeting this target resubmission date.

With your revision, please:

• Include a “Response to the editors and reviewers” document detailing, point-by-point, how you addressed each editor and referee comment. If no action was taken to address a point, you must provide a compelling argument. When formatting this document, please respond to each reviewer comment individually, including the full text of the reviewer comment verbatim followed by your

response to the individual point. This response will be used by the editors to evaluate your revision and sent back to the reviewers along with the revised manuscript.

• Highlight all changes made to your manuscript or provide us with a version that tracks changes.

Please use the link below to submit your revised manuscript and related files:

Link Redacted

Note: This URL links to your confidential home page and associated information about manuscripts you may have submitted, or that you are reviewing for us. If you wish to forward this email to co-authors, please delete the link to your homepage.

Thank you for the opportunity to review your work. Please do not hesitate to contact me if you have any questions or would like to discuss the required revisions further.

Sincerely,

[redacted]
Nature Human Behaviour

Reviewer expertise:

Reviewer #1: Genetics of cognitive performance

Reviewer #2: Cognitive genetics, common and rare genetic variation

Reviewer #3: Common and rare genetic variation; Biostatistics

REVIEWER COMMENTS:

Reviewer #1 (Remarks to the Author):

Dear authors and editor, thank you for the opportunity to review this manuscript, which I found really interesting and well written. Please find a few queries and comments below, some minor ones and some possibly important ones, e.g. attrition.

Heritablity estimates. Please provide confidence intervals in text around heritability estimates at all ages (e.g. line 119). Estimates of IQ heritability are quite high. Often, different estimation approaches give different results. E.g. S-LDSC method seem to provide more accurate and lower estimates. Are the authors able to apply alternative methods to estimate how their estimate of heritability (and thus change over time) is dependent (or not) on the choice of method.

Box 1 definitions.
Direct effect. “This effect isolates the influence of the child’s own genetic score on their own phenotype and has a causal interpretation”. This causal interpretation has been questioned recently in articles by Valler and Coop e.g. https://journals.plos.org/plosbiology/article?id=10.1371/journal.pbio.3002511

Please add reference for following assertion: “the coefficients on the parents’ scores are mathematically equivalent to the effect of the non-transmitted alleles in the parents”

Convergence between GWAS and rare variant findings. Is there any way of quantifying or conveying how strong this convergence is? E.g. should we conclude that rare variant analysis is not useful if we find that it only identifies signal already identified by GWAS?

Attenuation. There is a very substantial attenuation of effects for PGI in trio models, often interpreted as indicative of indirect genetic effects (which as the authors note are non trivial to interpret). But there is no attenuation for rare variants. If there were true indirect effects, shouldn’t these be observed for both common and rare variants? Can the authors discuss this discrepancy further.

Accounting for heteroscedasticity. The authors test whether genetic effects differ at the ends of the distribution, which is really interesting. My understanding is that this goes hand in hand with heteroscedasticity. Typical analyses require homoscedasticity as an assumption. If this is violated, i.e. there is heteroscedasticity in the genetic associations tested by the authors, then statistical models must be adapted (e.g. GLS rather than OLS) to avoid, for example, inadequate standard errors. So the question is: how did the authors account for heteroscedasticity in their primary analyses?
Interaction. The power to detect interactions is typically small. So what is the power to detect interaction at the 5th percentile for ex? Isn’t this the equivalent to testing interactions is a very small subset of an already small sample? And if this is the case, the authors should be careful when commenting on the absence of evidence of interaction, which does not mean much in terms of whether a true interaction is present or no.

Figure 3. The authors show an illustration of their approach in panel A. I think this is welcome. If anything, I would want the authors to put more effort into explaining the quantile regression to readers as most will be much less familiar with this than with typical

linear regression. The panel A was somewhat confusing to me. In particular because the Y axis is the outcome variable IQ, whereas for panel B, the Y axis is effect sizes, so there is no immediate equivalence between them although they look visually similar. So my understanding is that in panel A there is just one genetic effect on IQ, estimated by the regression slope, and the effect is similar across lest and right panels, just with homoscedasticity (left) vs heteroscedasticity (right). So the title of those panels ("uniform effects") is confusing as there is only one effect represented? And also, the link between this illustration of variance and panel B that represents different effect sizes is not clear. Again, this may stem from my lack of knowledge of quantile regression but this is an invitation to clarify further the presentation of those analyses. Still regarding figure 3, the text says "… but this is driven by there being more people with lower rather than higher IQ, illustrated in the right-hand plot in Figure 3A". But this seems reverse on the illustration 3Aright: median seems to be closer to 5% rather than 95%, so lower rather than higher variance for people with lower IQ?

Multiple analyses. In joint analyses, effects remained fairly similar except the effect of parental PGIEA which became not significant. This is similar to the 2018 Bates paper on genetic nurture where when they control for parental education the genetic nurture effect completely attenuates which is consistent with total mediation of that effect via phenotypic education (https://pubmed.ncbi.nlm.nih.gov/29530109/).

Measurement error. The authors show that several genetic effects seem to change with age. As the authors say, IQ measurement is less reliable early on. That, in itself could be sufficient to explain why genetic associations change with age. In the limitation section, the authors say on this topic that their findings "…replicate trends in other cohorts and with other cognitive performance measures". I would consider removing that argument. Attrition is common to the vast majority if not all cohorts. And most cognitive or noncognitive measures are less reliable at earlier ages. So, unless you can show that these results stand on a cohort with no attrition or on a measure that is demonstrated to have the same reliability, then I would remove. And maybe make clearer why you think that effects in opposite directions for common and rare variants is not compatible with measurement error.

Attrition. If I understand findings properly, rare variants may substantially reduce IQ and therefore increase variance in the lower tail of IQ and lead to stronger genetic effects in that tail of the distribution. However, I find quite puzzling that the size of effect would attenuate with age for rare variants? As per the authors' own discussion of the Matthew effect, initial individual differences in ability tend to compound over time, so, if anything, we should expect a strengthening of the effects. And there does not seem to be any good reason why this effect would only exist for phenotypic individual differences that are due to common variants vs rare variants. The authors mention some sort of compensation to explain this change: "due to acute, time-limited effects during early neurodevelopment that can be attenuated by later plasticity, potentially in response to environmental influence". If indirect genetic effects capture some of those environmental effects (i.e. if they are not entirely pop strat and assort mating), shouldn't they index this type of environmental compensation? Can the authors discuss this point and to which extent there findings of indirect effects are consistent or not with compensation occurring with age (e.g. increasing indirect effects with age?).
Instead, a technical artefact like attrition may explain those findings (as I assume there is no excess mortality in those children?). Attrition is typically (much) higher for children with difficulties (parents probably have other things to do than to respond to questionnaires). We can imagine that children with rare variants substantially affecting their development, might be more susceptible to attrition. And if there was such attrition, we would lose variance in that low tail region the authors focus on, as well as probably reduce the size of the genetic association. And given that these are the individuals at the end of the distribution, loosing just a few might make a substantial difference (analogous to outliers). This is all the more important as the N at age 4 is a small fraction of what is available at ages and then you have more classical attrition between 8 and 16.

Characterizing attrition. The question of missingness and attrition is thus particularly important for the interpretation of results. For that reason, I think that additional information should be included in the manuscript rather than just in the supplement (line 110). At the very least to clarify how many individuals were imputed. The authors say: "Our primary analyses were based on measures of IQ from ALSPAC that were collected at ages 4 (n=1,012), 8 (n=7,347), and 16 (n=5,270)". Can you please clarify explicitly in the manuscript: 1. What is the rule for imputation, I assume at least one IQ measure across the 3 ages; 2. What is the final sample size after imputation, which I assume will be > 7347 because of imperfect overlap across ages. 3. Specify explicitly for the reader how many IQ values were imputed at the different ages (i.e; basically total n for imputed sample minus observed values at each age). 4. Provide in the manuscript more descriptives to characterize attrition, e.g. is there a difference in average genetic burden scores at the different time points? Any difference in polygenic scores?

In brief, I would expect the authors to better characterize attrition and describe imputation in the manuscript and acknowledge more in depth the possibility that some of the core findings might results from such technical artefacts such as measurement error and attrition.

Minor:
L 138: sentence starting by "Importantly, we found that common…" is long and could be clearer.
Can't see titles for figures.

Reviewer #2 (Remarks to the Author):

This paper by Maawsky and colleagues pursues a very topical idea in neurodevelopmental genetics, namely that the genetic contribution to phenotypic variation depends not only on genetic variation, but expression across the lifespan. In particular, dynamic changes in gene expression chimes with the idea of sensitive periods of development for language and cognition and for neurodevelopmental disorders. In the complex series of analysis presented in this paper, based on public datasets (ALSPAC, UKB) the authors show that the effects of common and rare variants on cognition vary across childhood. Specifically, they report evidence that the effects of common variants strengthen with age, whereas as the effects of rare variants attenuate with age. Importantly, they also provide evidence that these dynamic effects also depend on position in the phenotypic distribution, with

genetic effects becoming either weaker or more penetrant towards either end of the distribution.

By considering the two axis of time (aim 1) and relative phenotypic distribution position (aim 2), the authors provide important evidence of the dynamic effects of common and rare genetic variation, advancing on previous studies in this area. This is important, for example, because of the studies relevance to understanding the incomplete penetrance of rare (deleterious) variants.

The authors also include (aim 3) consideration of a number of factors that are likely to impact on these relationships, including environmental factors (e.g. parental education) and differences in phenotypic expression between those with a familial relationships (e.g. to comment on ‘genetic nurture’). While this is a strength of the paper in my view, this it also made the results section of the paper long and quite challenging to read. This was further complicated by multiple phenotypes being used to index cognition and multiple samples. This complexity is illustrated e.g. in the 8-panel figure (Fig 1 and 2) and 10-panel figures (fig 3) which are difficult to interpret. I suspect these could be simplified, for example putting the non-imputed panels into supp data.

The authors discuss a number of factors that might explain the increase of heritability of cognitive performance up to age 18 (including genetic innovation, amplification, and the compounding effects of educational experience). Surprisingly the authors don’t explicitly discuss dynamic changes in gene expression or the pattern of differential gene expression at different stages in development. Neither do they link their findings to likely neurobiological changes in brain structure and functon(e.g. synaptic pruning).

Finally, the authors conclude that the future work suggested by their study should be to identify children at risk of poor clinical outcomes. I did not find this a compelling conclusion. Related to the point above, the next steps would I believe be about link these findings to longitudinal changes in brain development. If the authors do feel that their study has implications for genetic screening, these need to be made more explicit and concrete than the conjectures currently offered.

Reviewer #3 (Remarks to the Author):

In this paper, Malawsky et al. present a detailed analysis of common and rare-variant genetic influences on cognitive performance across development, using data from the ALSPAC, MCS, and UK Biobank studies. The primary genetic tool for common variant analysis is polygenic indices (PGIs) for EA, EA-Cog, and EANonCog. Rare variant burden (RVB), weighted using RGC-ME (Ref. 46), is used for rare variant analysis. The paper is well-structured and presents a clear and logical narrative; however, I have several major comments and concerns for the authors to consider.

(1) “Genetic effects” is a tricky term to use in the title and throughout the main text. In the context of PGI analysis for common variants, the authors interpret the associations between EA-PGI (or EA-Cog and EANonCog) and IQ as “population effects” from the PGI and IQ, with the “direct effects” inferred after adjusting for parental genetic scores. However, these are merely associations derived from mixed-effects models and do not necessarily imply a directional effect from EA-PGI to IQ, even after adjusting for parental genetic scores. In many genetic studies, “genetic effects” typically refer to causal relationships from genes or variants to phenotypes, which may lead to confusion if it is used to interpret PGI-based associations. A more precise framing of these associations could improve clarity.

(2) One key finding is that the association between EA-PGI and IQ varies across the three developmental time points. Since EA-PGIs are derived from GWAS on adult education attainment (EA), their predictive power may be stronger for age 16 IQ than for earlier ages, simply due to the nature of the discovery GWAS sample. As the authors briefly acknowledged (Line 384), there are multiple potential explanations for this pattern, beyond the “changing genetic effects”. For example, if phenotypic IQ consists of both a genetic component (IQ-G, which is heritable) and a non-genetic component (IQ-nonG), then the EA-PGI and IQ association could be decomposed to “EA-PGI and IQ-G” (e.g., genetic correlation between the two phenotypes) and “EA-PGI and IQ-nonG” (e.g., potential confounding effects). Changes in either of these parts could drive changes in the observed EA-PGI and IQ total association, making it difficult to interpret these results solely as shifts in “genetic effects” of EA-PGI on IQ.

(3) Another key finding is the difference in the role of common versus rare variants. I feel this may not be entirely surprising given their distinct definitions and estimation methods. RVB scores are additive burden scores weighted by evolutionary constraint/negative selection, making them conceptually closer to traditionally defined “genetic effects”. Therefore, it is unclear whether the changing effect patterns of these burden scores are comparable to the changing patterns of PGI-based associations with IQ, as they may measure fundamentally different concepts. Comparing PGI association results between two phenotypes to RVB results of one phenotype may be conceptually similar to comparing apples to oranges, the interpretation may require careful consideration.

(4) A more direct approach to assessing common vs. rare variant effects on cognitive performance would be to estimate variant/gene-level genetic effects in a large cohort and compare them. However, current sample sizes may be too limited to allow for such direct comparisons, especially for rare variant analysis. Instead, here the authors compare them using PGI and RVB tools, but as outlined in previous comments, the interpretation of their differences as “different genetic effects” may be challenging.

Version 1:

Decision Letter:

3rd September 2025

Dear Mr Malawsky,

Thank you once again for your revised manuscript, entitled "Differential associations of common and rare genetic variants with cognitive performance across development," and for your patience during the re-review process.

Your manuscript has now been evaluated by the same reviewers who evaluated your original manuscript. All reviewer feedback is included at the end of this letter. Although the reviewers found your manuscript to have improved during revision, they also raise some important outstanding concerns. We remain interested in the possibility of publishing your study in Nature Human Behaviour, but would like to consider your response to these outstanding concerns in the form of a revised manuscript before we make a decision on publication.

In particular, Reviewer #3 remains concerned about the direct comparison between PGI and RVB based analyses and is not convinced that your additional analyses addressed the issue of insufficient power of rare variants. In your revision, please fully address these concerns by carefully reframing your work, consider the fundamental differences between PGI and RVB constructs, and avoid making direct comparisons especially in your Discussion section.

Besides, Reviewer 3 reiterates their concerns about the use of "genetic effects". Editorially I'd agree: the word "effect" implies causality, but the evidence presented is correlational, since regression-based models do not provide evidence for causality. Throughout the paper, please replace "genetic effects" with "genetic associations" or "genetic variants associated with XXX"

Finally, your revised manuscript must comply fully with our editorial policies and formatting requirements. Failure to do so will result in your manuscript being returned to you, which will delay its consideration. To assist you in this process, I have attached a checklist that lists all of our requirements. If you have any questions about any of our policies or formatting, please don't hesitate to contact me.

In sum, we invite you to revise your manuscript taking into account all reviewer and editor comments. We are committed to providing a fair and constructive peer-review process. Do not hesitate to contact us if there are specific requests from the reviewers that you believe are technically impossible or unlikely to yield a meaningful outcome.

We hope to receive your revised manuscript within 4-8 weeks. I would be grateful if you could contact us as soon as possible if you foresee difficulties with meeting this target resubmission date.

With your revision, please:

• Include a “Response to the editors and reviewers” document detailing, point-by-point, how you addressed each editor and referee comment. If no action was taken to address a point, you must provide a compelling argument. This response will be used by the editors and reviewers to evaluate your revision.

• Highlight all changes made to your manuscript or provide us with a version that tracks changes.

Please use the link below to submit your revised manuscript and related files:

Link Redacted

Note: This URL links to your confidential home page and associated information about manuscripts you may have submitted, or that you are reviewing for us. If you wish to forward this email to co-authors, please delete the link to your homepage.

We look forward to seeing the revised manuscript and thank you for the opportunity to review your work. Please do not hesitate to contact me if you have any questions or would like to discuss these revisions further.

Sincerely,

[redacted]
Nature Human Behaviour

Reviewer expertise:

Reviewer #1: Genetics of cognitive performance

Reviewer #2: Cognitive genetics, common and rare genetic variation

Reviewer #3: Common and rare genetic variation; Biostatistics

REVIEWER COMMENTS:

Reviewer #1 (Remarks to the Author):

The authors have comprehensively addressed my comments and, even when they did not follow my recommendations, their arguments are appropriately justified.

Reviewer #2 (Remarks to the Author):

The authors have adequately responded to my queries and are to be commended on an interesting manuscript which will contribute to the field.

Reviewer #3 (Remarks to the Author):

(1) I agree that the term "genetic effects" is often used in the literature; however, in my view, its appropriateness largely depends on the context and the specific phenotype pairs being studied and thus needs careful consideration. In particular, for genetic associations between EA and IQ, the relationships can be very complex, and using the term "genetic effects" may be easily misleading when others interpret the findings.

(2) I appreciate the authors' efforts to clarify their reasoning regarding the varying association between EA-PGI and IQ. Their current argument, which combines multiple lines of evidence, is stronger than previous ones. I would encourage the authors to explicitly draw on these converging pieces of evidence when interpreting their findings in the main text. This would provide a more systematic explanation and justification for the age-related increases in EA-PGI and IQ association.

(3) I thank the authors for addressing my question about the "common/rare comparisons". After reviewing the responses, I am afraid my original concerns still remain.
I am not fully convinced that the argument directly addresses my question: "Both scores are constructed to model an individual's additive genetic liability to IQ. The PGI does so in the part of the allele-frequency spectrum where single-variant effect estimates are reliable..." In this study, the authors are using a PGI for EA, not IQ. The PGI therefore reflects additive genetic effects on EA, and the major analysis focuses on the genetic association strength between this EA-PGI and IQ, which is interpreted as common genetic effects on IQ. While the authors explain their use of the shet score in rare variant analysis by including the new Extended Data Figure 3, this does not explicitly address my question. My concerns about comparing the "EA-IQ genetic association pattern" with the RVB results remain. I find it difficult to support drawing strong (!) conclusions about differences in the roles of common versus rare variants based on the current evidence.

(4) Thank you to the authors for their efforts in conducting new analyses. These results further support my original view that the current datasets may lack sufficient power to address this research question. However, this does not justify interpreting the current findings as evidence of "different genetic effects".
While I appreciate the interesting research question this study seeks to address, I really find it difficult to get behind the concept of this comparison and support the way to interpret their difference, given the fundamental differences between "EA-IQ genetic associations" and RVB burden tests of IQ.

Version 2:

Decision Letter:

17th December 2025

Dear Mr Malawsky,

Thank you once again for your revised manuscript, entitled "Common and rare genetic variant associations with cognitive performance across development," and for your patience during the re-review process.

Your manuscript has now been evaluated by Reviewer #3 from the previous round of review. All feedback is included at the end of this letter. Although the reviewer found your manuscript to have improved during revision, they also raise some important outstanding concerns. We remain interested in the possibility of publishing your study in Nature Human Behaviour, but would like to consider your response to these outstanding concerns in the form of a revised manuscript before we make a decision on publication.

In particular, please respond to the reviewer's remaining concern by adding additional analysis using a PGI constructed for cognitive performance as Reviewer #3 suggests.

Finally, your revised manuscript must comply fully with our editorial policies and formatting requirements. Failure to do so will result in your manuscript being returned to you, which will delay its consideration. To assist you in this process, I have attached a

checklist that lists all of our requirements. If you have any questions about any of our policies or formatting, please don't hesitate to contact me.

In sum, we invite you to revise your manuscript taking into account all reviewer and editor comments. We are committed to providing a fair and constructive peer-review process. Do not hesitate to contact us if there are specific requests from the reviewers that you believe are technically impossible or unlikely to yield a meaningful outcome.

We hope to receive your revised manuscript within 4-8 weeks. I would be grateful if you could contact us as soon as possible if you foresee difficulties with meeting this target resubmission date.

With your revision, please:

• Include a “Response to the editors and reviewers” document detailing, point-by-point, how you addressed each editor and referee comment. If no action was taken to address a point, you must provide a compelling argument. This response will be used by the editors and reviewers to evaluate your revision.

• Highlight all changes made to your manuscript or provide us with a version that tracks changes.

Please use the link below to submit your revised manuscript and related files:

Link Redacted

Note: This URL links to your confidential home page and associated information about manuscripts you may have submitted, or that you are reviewing for us. If you wish to forward this email to co-authors, please delete the link to your homepage.

We look forward to seeing the revised manuscript and thank you for the opportunity to review your work. Please do not hesitate to contact me if you have any questions or would like to discuss these revisions further.

Sincerely,

[redacted]
Nature Human Behaviour

Reviewer expertise:

Reviewer #3: Genetics; Biostatistics

REVIEWER COMMENTS:

Reviewer #3 (Remarks to the Author):

I thank the authors for their efforts in addressing my previous comments. I have some follow-up questions and suggestions regarding comment (3) on the “EA–IQ genetic associations” and the RVB burden tests of IQ. I appreciate that the authors now have a clearer understanding of this question and that they have more carefully expanded the discussion, and I find that the revised interpretations have been improved.

(i) The authors note that “We apologise for our carelessness in claiming previously that the EA-PGI was constructed to model an individual’s additive genetic liability IQ, which is of course incorrect. We do note, however, that in our main analyses we used not only a PGI for EA, but also a PGI for the cognitive component of EA (from Demange et al., Nature Genetics, 2021), which is effectively a PGI for adult cognitive performance (with some caveats/nuances, of course)...”

Could the authors use the most highly powered, publicly available GWAS of **real** cognitive performance/IQ phenotype to construct a **real** PGI for cognitive performance (CP-PGI), redo the association analyses, and examine whether they obtain similar conclusions to those based on the EA-PGI and the PGI for the cognitive component of EA, particularly with respect to their main conclusion comparing the “polygenic indices association" and the "RVB association”?

If the EA-PGI and CP-PGI yield similar conclusions, this would substantially strengthen the authors’ arguments. In that case, it would also be helpful to explain why a CP-PGI was not used from the outset, and whether this motivates any proper revision of the analysis plan or framing.

If, however, the CP-PGI and EA-PGI lead to different conclusions, this would suggest that the current reasoning may not fully hold and would require more careful investigation.

(ii) Unless the authors decide to use a real cognitive performance GWAS to construct CP-PGI as their primary analysis, it is important to specify the source of the “polygenic indices consistently”. In particular, whenever “polygenic indices” are mentioned,

please clarify that they are derived from EA (or from the cognitive component of EA). Otherwise, readers may assume they come from a cognitive performance GWAS.

For example, in the abstract, where the text currently refers to “between polygenic indices and” and “associations with polygenic indices,” it should be made explicit that these are EA-based polygenic indices; otherwise, this wording is somewhat misleading.

Version 3:

Decision Letter:

Our ref: NATHUMBEHAV-25010040C

20th January 2026

Dear Dr. Malawsky,

Thank you for submitting your revised manuscript "Common and rare genetic variant associations with cognitive performance across development" (NATHUMBEHAV-25010040C). We have editorially evaluated your revision find that the paper has improved in revision. We will be happy in principle to publish it in Nature Human Behaviour, pending minor revisions to satisfy some final requests and to comply with our editorial and formatting guidelines.

We are now performing detailed checks on your paper and will send you a checklist detailing our editorial and formatting requirements within two weeks. Please do not upload the final materials and make any revisions until you receive this additional information from us.

Please do not hesitate to contact me if you have any questions.

Sincerely,

[redacted]
Nature Human Behaviour

Version 4:

Decision Letter:

Dear Dr Malawsky,

We are pleased to inform you that your Article "Common and rare genetic variant associations with cognitive performance across development in British birth cohorts", has now been accepted for publication in Nature Human Behaviour.

Authors may need to take specific actions to achieve compliance with funder and institutional open access mandates. If your research is supported by a funder that requires immediate open access (e.g. according to Plan S principles or the NIH public access policy) then you should select the gold OA route, and we will direct you to the compliant route where possible. Because authors warrant under our subscription licensing terms that they haven’t committed to licensing any version of their article under a licence inconsistent with the terms of our agreement – including the applicable embargo period – publication under the subscription model isn’t suitable for authors whose funders require no embargo.

Once your manuscript is typeset and you have completed the appropriate grant of rights, you will receive a link to your electronic proof via email with a request to make any corrections within 48 hours. If, when you receive your proof, you cannot meet this deadline, please inform us at rjsproduction@springernature.com immediately. Once your paper has been scheduled for online publication, the Nature press office will be in touch to confirm the details.

Acceptance of your manuscript is conditional on all authors' agreement with our publication policies (see http://www.nature.com/nathumbehav/info/gta). In particular your manuscript must not be published elsewhere and there must be no announcement of the work to any media outlet until the publication date (the day on which it is uploaded onto our web site).

If you have posted a preprint on any preprint server, please ensure that the preprint details are updated with a publication

reference, including the DOI and a URL to the published version of the article on the journal website.

An online order form for reprints of your paper is available at https://www.nature.com/reprints/author-reprints.html. All co-authors, authors' institutions and authors' funding agencies can order reprints using the form appropriate to their geographical region.

We welcome the submission of potential cover material (including a short caption of around 40 words) related to your manuscript; suggestions should be sent to Nature Human Behaviour as electronic files (the image should be 300 dpi at 210 x 297 mm in either TIFF or JPEG format). Please note that such pictures should be selected more for their aesthetic appeal than for their scientific content, and that colour images work better than black and white or grayscale images. Please do not try to design a cover with the Nature Human Behaviour logo etc., and please do not submit composites of images related to your work. I am sure you will understand that we cannot make any promise as to whether any of your suggestions might be selected for the cover of the journal.

You can now use a single sign-on for all your accounts, view the status of all your manuscript submissions and reviews, access usage statistics for your published articles and download a record of your refereeing activity for the Nature journals.

To assist our authors in disseminating their research to the broader community, our SharedIt initiative provides you with a unique shareable link that will allow anyone (with or without a subscription) to read the published article. Recipients of the link with a subscription will also be able to download and print the PDF.

As soon as your article is published, you will receive an automated email with your shareable link.

In approximately 10 business days you will receive an email with a link to choose the appropriate publishing options for your paper and our Author Services team will be in touch regarding any additional information that may be required.

If you have any questions about our publishing options, costs, Open Access requirements, or our legal forms, please contact ASJournals@springernature.com

We look forward to publishing your paper.

With best regards,

[redacted]
Nature Human Behaviour

P.S. Click on the following link if you would like to recommend Nature Human Behaviour to your librarian http://www.nature.com/subscriptions/recommend.html#forms

** Visit the Springer Nature Editorial and Publishing website at www.springernature.com/editorial-and-publishing-jobs for more information about our career opportunities. If you have any questions please click here.**

Open Access This Peer Review File is licensed under a Creative Commons Attribution 4.0 International License, which permits use, sharing, adaptation, distribution and reproduction in any medium or format, as long as you give appropriate credit to the original author(s) and the source, provide a link to the Creative Commons license, and indicate if changes were made.
In cases where reviewers are anonymous, credit should be given to 'Anonymous Referee' and the source.
The images or other third party material in this Peer Review File are included in the article's Creative Commons license, unless indicated otherwise in a credit line to the material. If material is not included in the article's Creative Commons license and your intended use is not permitted by statutory regulation or exceeds the permitted use, you will need to obtain permission directly from the copyright holder.
To view a copy of this license, visit https://creativecommons.org/licenses/by/4.0/

Editor’s comments

- Editorially we are concerned that you use causal language throughout your work, and discuss policy implications, yet report only correlational findings, and this concern is also emphasised by both Reviewer #1 and Reviewer #3. Please remove all causal language, and please also remove all policy implications that stem from a causal interpretation of your results (e.g. genetic screening), since these do not apply here. When interpreting your findings, please be clear that you can speak to association only and not causality. Please also note this in the Limitations section of your Discussion

We have removed all causal language and policy implications in the main text and in Box 1, reframing our findings strictly as associations (e.g. “within-family prediction”). We have also removed any discussion of genetic screening or other policy applications based on causality. We have modified the title of the article to reflect the observational nature of the dataset in response to Reviewer 3. In Box 1, we have removed the claim that “direct genetic effects” have a causal interpretation and instead now say: “This effect quantifies within-family prediction of the score, independently of genetic nurture, parental assortment, and uncontrolled population stratification.” We also added a sentence at the end of the Limitations paragraph of the Discussion: “Finally, because our study is based on observational data, our results should be viewed as identifying genetic associations, both between- and within-family predictions, rather than as causal estimates of genetic effects.” We hope these changes fully address the editors’ and reviewers’ concerns.

- Reviewer #1 raised major concerns regarding the effect of attrition on your results, as well as insufficient information on your imputation methods. Please clarify these issues explicitly as suggested by Reviewer #1

We have now characterized attrition patterns across age measurements (new additions to Supplementary Note 1, Supplementary Table 10, Extended Data Figure 1C). We found significant but very weak associations between IQ missingness patterns and PGIs (explaining 0.55%-1.9% of variance) and no associations with rare variant burden scores. We also conducted various robustness analyses (new Supplementary Note 8) to test whether attrition could have created spurious genetic effects. In brief, these analyses were:
- We repeated analyses excluding age 4 IQ measurements entirely, using only ages 8 and 16, and found concordant age interaction estimates (Supplementary Table 12).

- We also performed simulation studies testing whether observed attrition patterns could induce spurious genetic effects and found that attrition produced minimal bias and is highly unlikely to account for our findings.

Furthermore, we also emphasized the fact that we had replicated findings using school grades at ages 11-14, where dropout was <3%.
We enhanced the description of imputation methods in the Results section, clarifying imputation rules, final sample sizes after imputation, number of imputed values at each age, and descriptive statistics characterizing attrition patterns. These analyses collectively demonstrate that attrition is highly unlikely to have driven the observed differential developmental trajectories of common versus rare variant effects on cognition, addressing the reviewer's concerns about the validity of our core findings.

- Reviewer #1 also pointed out potential issues with heteroscedasticity. Please respond to these comments by clarifying how you accounted for heteroscedasticity in your primary analyses, or conduct relevant analyses as robustness check if you have not done so.

To address Reviewer #1's concerns about heteroskedasticity, we acknowledged that while mixed-effects models produce unbiased effect size estimates under heteroskedasticity, standard errors can be biased if residual variances differ across observations. We re-estimated all key mixed-effect models using parametric bootstrap of fixed-effect coefficients and now report bootstrapped standard errors and p-values in Supplementary Tables 4 and 6. Across PGI analyses, 50% of bootstrap p-values were smaller than the originals; across RVB analyses, 59% were smaller. Bootstrap-based inference did not alter our conclusions using identical p-value thresholds, indicating that heteroskedasticity did not systematically lead to under-estimated standard errors or anti-conservative p-values in our analyses. We added text to the Discussion noting heteroskedasticity had minimal impact on our conclusions.

- Reviewer #1 and #3 have raised further concerns on your analytical approach and interpretation of results, such as underpowered interaction (Reviewer #1) and inadequate comparison of effect patterns between PGI-based associations and RVB scores. Please respond to these comments and revise your manuscript appropriately.

To address concerns about analytical approach and interpretation, we clarified several interpretations and approaches. For example, we addressed concerns regarding interpretation of the quantile regression, noting that quantile regression uses all observations to estimate effects at specific quantiles rather than subsetting data, providing comparable statistical power to detect effects at the 5th and 95th percentiles when the distribution is symmetric. The differential patterns we observe (PGI x age interactions significant at 95th percentile but not 5th, with RVB x age interactions showing the opposite pattern) suggest genuine heterogeneity rather than power artifacts. We acknowledged that sample size may limit power to detect subtle interaction effects and revised language to avoid implying absence of evidence equals evidence of absence.

Regarding PGI-RVB comparisons, we clarified that both scores model additive genetic liability to IQ across different parts of the allele frequency spectrum. We demonstrated that evolutionary constraint scores (S_{het}) used for RVB weighting correlate linearly with rare variant effects on fluid intelligence in UK Biobank (new Extended Data Figure 3), validating our weighting approach. We also conducted analyses attempting to use direct estimates of a gene’s effect of cognitive ability as weights for constructing the rare variant burden score, but found it underpowered compared to our S_{het}-based approach.

We also changed the title to replace "effects" with "associations" and added clarification in Box 1 that "genetic effect" terminology follows field standards without implying causality. These revisions address concerns about comparing different genetic scoring methodologies and interpretation of interaction analyses.

Reviewer #1 (Remarks to the Author):

Dear authors and editor, thank you for the opportunity to review this manuscript, which I found really interesting and well written. Please find a few queries and comments below, some minor ones and some possibly important ones, e.g. attrition.

Heritability estimates. Please provide confidence intervals in text around heritability estimates at all ages (e.g. line 119). Estimates of IQ heritability are quite high. Often, different estimation approaches give different results. E.g. S-LDSC method seem to provide more accurate and lower estimates. Are the authors able to apply alternative methods to estimate how their estimate of heritability (and thus change over time) is dependent (or not) on the choice of method.

We thank the reviewer for their thoughtful comments on our heritability estimates. We have now added confidence intervals for all heritability estimates at each age point in the text as suggested (line 102).

Regarding the suggestion to use S-LDSC as an alternative method, we respectfully disagree that it would provide more accurate estimates in our study, for several key reasons:

1. Ni et al. demonstrated that S-LDSC is a biased estimator of heritability, while GREML-LDMS provides more robust and accurate estimates.[1] They note that "LDSC and sLDSC estimates for SNP heritability were biased in the genome partitioning analysis" whereas "GREML estimates were close to the true value in estimation of SNP heritability."
2. The original authors of LDSC and S-LDSC specifically note that these methods require “strong assumptions about the effect sizes of rare variants”[2] and were "not recommended for SNP heritability by the original authors, but rather only for relative enrichment analysis"[1]. Despite this explicit limitation acknowledged by the method's developers, these approaches continue to be used for heritability estimation primarily due to convenience (in particular, the ability to apply them to GWAS summary statistics) rather than methodological superiority. However, given we have access to individual-level data, we were able to use the more robust GREML-LDMS methodology to estimate heritability in our study. Although we correct for genetic PCs in our relatively geographically and genetically homogenous sample and consider unrelated individuals, uncontrolled population stratification could still theoretically bias these estimates. However, the primary analysis of interest is that of changing variance explained with age in *the same individuals*, making uncontrolled population stratification less of a concern.
3. Similarly, the developers of S-LDSC themselves acknowledge significant limitations in heritability estimation at low sample sizes using their method (N<10,000). In their analysis of UK Biobank phenotypes, they identified a downward bias in their method’s heritability estimation at small sample sizes: "GWAS with small effective sample sizes have insufficient power for LDSR to detect polygenic effects, leading to near-zero estimates of heritability" with this issue particularly pronounced "below an effective sample size of 5,000-10,000"[3] . Given our sample size is in this range, it would be inappropriate to use LDSC for heritability estimation even if one believes it to be a reliable estimator of heritability.
4. Our heritability estimates are consistent with those from robust estimation methods using molecular genetic data obtained from sibling pairs. Recent work using sib regression, where sibling pairs’ phenotypic similarity is regressed on their realized genetic relatedness (a robust estimator of within-family heritability), found cognitive performance heritability estimates of approximately 0.75 (95% CI: 0.36-1) in an adult sample[4], which is comparable to our estimate of 0.56 (95% CI:0.40-0.71) at age 16 in ALSPAC. This convergence between our GREML-

LDMS estimates and those from methodologically distinct approaches provides additional validation for our findings.

5. Lastly, our results obtained via PGI and genetic correlation analyses imply that the common variant heritability is increasing with age. As derived in [5], since the genetic correlations across ages (or “environments” in their framework) were not significantly different from one, but the variance explained by the PGIs increased with age, this implies that the common variant heritability increased with age, providing complementary evidence to support the finding that (GREML-LDMS) heritability increases with age.

We acknowledge that different estimation approaches can yield different results, as the reviewer notes. However, the current evidence indicates that GREML-based methods provide more reliable absolute heritability estimates than S-LDSC, and our findings align with those from other robust and complementary methodological approaches in both our own and other cohorts. Our relatively high heritability estimates (ranging from 0.46-0.56 post-imputation) are not unusually high but are consistent with both previous twin studies[6,7] and more recent molecular approaches to estimating the heritability of cognitive performance, with one recent estimate using sib-regresion of 0.746 in a meta-analysis of adults cohorts [4].

To clarify the implications of the PGI results described in point #5 above, we added the following to the Supplementary Discussion (line 893 of Supplementary Text):

“Moreover, recent work has demonstrated that observing an increasing PGI effect with age while the genetic correlation does not significantly deviate from one implies that the common variant heritability increased with age, providing an additional line of evidence supporting the Wilson effect.”

Box 1 definitions.
Direct effect. “This effect isolates the influence of the child’s own genetic score on their own phenotype and has a causal interpretation”. This causal interpretation has been questioned recently in articles by Valler and Coop e.g. https://journals.plos.org/plosbiology/article?id=10.1371/journal.pbio.3002511 [journals.plos.org]

We thank the reviewer for this comment. Given the ongoing debates around the precise interpretation of effects estimated within-family, we have removed mentions of causality in Box 1 and throughout the text.

Please add reference for following assertion: “the coefficients on the parents’ scores are mathematically equivalent to the effect of the non-transmitted alleles in the parents”

Apologies for omitting the reference here. This notion was introduced by Young et al., Nature Genetics, 2022. We have now added a citation to it.

Convergence between GWAS and rare variant findings. Is there any way of quantifying or conveying how strong this convergence is? E.g. should we conclude that rare variant analysis is not useful if we find that it only identifies signal already identified by GWAS?

We thank the reviewer for this interesting question. Indeed, studying the quantitative properties of convergence on genes implicated by studies of common and rare variants is an active, ongoing area

of research. For example, recent preprints studying this question systematically across several phenotypes have found that common and rare variant studies will differentially prioritize genes based on various genic properties including their degree of pleiotropy as well as the gene's physical length [8], though the two approaches tend to converge on identifying genes in the same biological pathways[9]. However, we believe that such analyses are beyond the scope of our work, which is not looking to discover common and rare variant associations with a trait *de novo*, but rather to compare how the effects of common and rare variants already known to be associated with traits related to cognition change across ages.

Attenuation. There is a very substantial attenuation of effects for PGI in trio models, often interpreted as indicative of indirect genetic effects (which as the authors note are non trivial to interpret). But there is no attenuation for rare variants. If there were true indirect effects, shouldn't these be observed for both common and rare variants? Can the authors discuss this discrepancy further.

We thank the reviewer for drawing attention to this observation. We have added the paragraph below to the Discussion to describe plausible explanations:

"Notably, we observed no attenuation for rare variant direct effects relative to their population effects, in contrast to the substantial attenuation observed for PGIs. There are several potential explanations for this. For one, a key factor may be the differential age-related dynamics we observe for these two classes of variants. Given that rare variant effects attenuate with age while common variant effects strengthen, the cognitive ability of parents carrying deleterious rare variants is expected to be less impacted by these variants than it was in childhood, potentially resulting in minimal genetic nurture effects through parental phenotypes. In contrast, since the PGI effects become more pronounced in adulthood, they may result in genetic nurture effects by affecting parental adult traits. Additionally, the strength of assortative mating directly influences the within-family effect size attenuations in trio models, and we observed that assortative mating patterns differ substantially between common and rare variants: there was a notable difference in the within-partner correlations for PGI_{EA} (r = 0.34, 95% CI: 0.33-0.37) and PGI_{Cog} (0.14, [0.12-0.16]) versus RVB_{pLoF} (0.086, [0.02-0.15]) and $RVB_{Missense}$ (-0.02, [-0.07-0.5]). Additionally, the impact of assortative mating may differ between rare and common variant analyses as it has been observed that assortative mating may occur nonuniformly across the spectrum of cognitive abilities.[10] Lastly, we note that the wider confidence intervals for rare variant estimates in the trio models limit our statistical power to detect attenuation; the point estimates alone cannot rule out modest indirect effects. Nonetheless, our findings suggest that indirect genetic effects may not operate uniformly across all types of genetic variation and may depend critically on the developmental timing of genetic effects and their influence on the phenotypic spectrum for traits relevant to partner selection."

Accounting for heteroscedasticity. The authors test whether genetic effects differ at the ends of the distribution, which is really interesting. My understanding is that this goes hand in hand with heteroscedasticity. Typical analyses require homoscedasticity as an assumption. If this is violated, i.e. there is heteroscedasticity in the genetic associations tested by the authors, then statistical models must be adapted (e.g. GLS rather than OLS) to avoid, for example, inadequate standard errors. So the question is: how did the authors account for heteroscedasticity in their primary analyses?

We thank the reviewer for this important point. While the mixed- effects models produce unbiased estimates of effect sizes even under heteroskedasticity, we agree that the typical analytic standard errors (SEs) can be biased if residual variances differ across observations. To address this, we have re-estimated all of our key mixed-effect models using a parametric bootstrap of the fixed-effect coefficients (1000 replicates per model), and report those SEs and p-values in Supplementary Tables S4 and S6.

Across the PGI analyses, 50% of the bootstrap p-values were smaller than the originals; across the RVB analyses, 59% were smaller. In no case did the bootstrap- based inference alter our conclusions using identical p-value thresholds. This pattern indicates that any heteroskedasticity in our data does not systematically lead to under-estimated SEs or anti- conservative p-values in our original analyses.

We modified the Results to highlight that bootstrap-estimated p values did not change any conclusions as follows:
"We note that our results here imply that there is heteroskedasticity (i.e. the variance of the phenotype varies with genotype), which could violate assumptions regarding the estimation of standard errors in our mixed-effects models in Figures 1 and 2. However, re-estimating several of our key models with bootstrap-derived standard errors produced virtually identical results (Tables S4 and S6), suggesting that any heteroskedasticity had minimal impact on our earlier analyses."

We also added the following to the Methods section:

"For our primary models, in addition to Wald- test p- values, we performed a parametric bootstrap of the fixed- effect estimates (1,000 replicates) to ensure our inference was robust to the heteroskedasticity we observed in the quantile regression models described later. To do this, we used bootMer (type = "parametric") in lme4. Bootstrap standard errors and p- values are reported in Tables S4 and S6."

Interaction. The power to detect interactions is typically small. So what is the power to detect interaction at the 5th percentile for ex? Isn't this the equivalent to testing interactions is a very small subset of an already small sample? And if this is the case, the authors should be careful when commenting on the absence of evidence of interaction, which does not mean much in terms of whether a true interaction is present or no.

It's important to clarify that quantile regression does not subset the data to only those individuals at a given percentile. Rather, it uses all observations to estimate effects at specific quantiles of the outcome distribution. With a relatively symmetric distribution like that of IQ, we should have comparable statistical power to detect effects at the 5th and 95th percentiles. The differential patterns we observe (i.e. PGI X age interactions being significant at the 95th percentile but not the 5th, and RVB X age interactions showing the opposite pattern) are therefore unlikely to be artifacts of differential power across quantiles. Instead, these results suggest genuine heterogeneity in how genetic effects change with age across the phenotypic distribution. Nevertheless, we acknowledge that our overall sample size, particularly in trio analyses, may limit power to detect more subtle interaction effects. We also note that, in showing the 95% confidence intervals, we are accurately representing the uncertainty with which we estimated the interaction effects at a given quantile. We also reviewed the language to ensure we do not imply that the absence of significant interaction does not necessarily imply none exist (e.g. in line 221 we write "There was no evidence for significant age interactions at the 5th percentile for either PGI.").

Figure 3. The authors show an illustration of their approach in panel A. I think this is welcome. If anything, I would want the authors to put more effort into explaining the quantile regression to readers as most will be much less familiar with this than with typical linear regression. The panel A was somewhat confusing to me. In particular because the Y axis is the outcome variable IQ, whereas for panel B, the Y axis is effect sizes, so there is no immediate equivalence between them although they look visually similar. So my understanding is that in panel A there is just one genetic effect on IQ, estimated by the regression slope, and the effect is similar across left and right panels, just with homoscedasticity (left) vs heteroscedasticity (right). So the title of those panels ("uniform effects") is confusing as there is only one effect represented? And also, the link between this illustration of variance and panel B that represents different effect sizes is not clear. Again, this may stem from my lack of knowledge of quantile regression but this is an invitation to clarify further the presentation of those analyses. Still regarding figure 3, the text says "… but this is driven by there being more people with lower rather than higher IQ, illustrated in the right-hand plot in Figure 3A". But this seems reverse on the illustration 3Aright: median seems to be closer to 5% rather than 95%, so lower rather than higher variance for people with lower IQ?

We apologize for the lack of clarity in the initial captioning of the figure. We have now split out the schematic to a separate Extended Data Figure 7 and expanded the caption to include a more thorough caption for the figure (See below). Crucially, we explain that in the case we illustrated on the right, the genetic measure is meant to reflect the association we observed with RVB_{pLoF}, where a higher value is associated with lower IQ, and the effect is primarily driven by a stronger effect on the 5th percentile of the IQ distribution.

Extended Data Figure 7

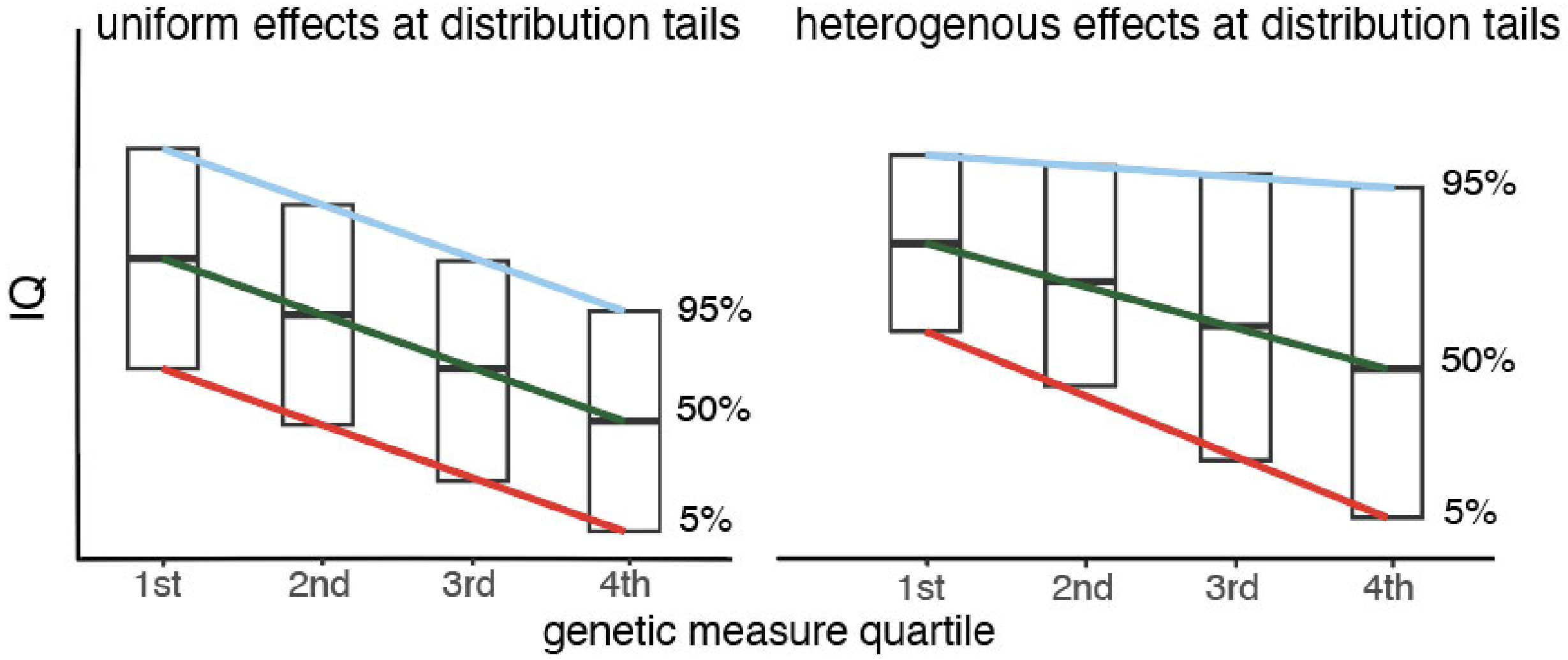

Schematic illustrating the difference between homoskedasticity and heteroskedasticity in terms of their effects on the relationship between a phenotype (IQ) and a genetic measure with which it is associated. The boxes show the distribution of the phenotype for individuals in the indicated quartile of the genetic measure (with the three bars representing the 5th, 50th and 95% percentile respectively), and the red, green and blue lines indicate the association between the genetic measure and the phenotype at those three quantiles. In the lefthand scenario, the genetic measure has uniform effects across the IQ distribution, and hence the coloured lines are parallel. In the righthand scenario, the genetic measure has heterogeneous effects on IQ across the

IQ distribution, with a larger effect on the 5th percentile than the 50th or 95th. This righthand scenario is what we observed for RVB_{pLoF} and $RVB_{Missense}$ at age 4 (assuming that the 4th quartile represents the highest RVB_{pLoF} score), which show larger effects on the 5th than 50th or 95th percentile (Figure S4).

-

Multiple analyses. In joint analyses, effects remained fairly similar except the effect of parental PGIEA which became not significant. This is similar to the 2018 Bates paper on genetic nurture where when they control for parental education the genetic nurture effect completely attenuates which is consistent with total mediation of that effect via phenotypic education (https://pubmed.ncbi.nlm.nih.gov/29530109/ [pubmed.ncbi.nlm.nih.gov]).

Thank you for pointing this out. We have now added a comment about this, and cited Bates et al. [line 274] We note that actually when fitting the quantile regressions (Figure S10B, formerly Figure 4B), some maternal PGIs are still nominally significant for the 5th and 50th percentile in the joint model after controlling for parental EA, and paternal PGI is still nominally significant for the 95th quantile. If replicated in other datasets, it could potentially point at some differences in the effects of maternal versus paternal nurture at different points in the IQ distribution.

Measurement error. The authors show that several genetic effects seem to change with age. As the authors say, IQ measurement is less reliable early on. That, in itself could be sufficient to explain why genetic associations change with age. In the limitation section, the authors say on this topic that their findings "…replicate trends in other cohorts and with other cognitive performance measures". I would consider removing that argument. Attrition is common to the vast majority if not all cohorts. And most cognitive or noncognitive measures are less reliable at earlier ages. So, unless you can show that these results stand on a cohort with no attrition or on a measure that is demonstrated to have the same reliability, then I would remove. And maybe make clearer why you think that effects in opposite directions for common and rare variants is not compatible with measurement error.

We thank the reviewer for the opportunity to clarify why measurement error alone could not drive the opposing age interaction effects. Classical measurement error takes the form of introducing uncorrelated noise to the IQ measurement, which would bias estimates of the variance explained between a given genetic score and IQ measurement towards 0. Thus, if the variance explained by each genetic score remained constant and the only difference across ages was a change in measurement error, we would expect all genetic measures to explain more variance with age, i.e., age interactions that increase the absolute value of the estimated genetic effects with age. However, we observe that, while the variance in IQ explained by the PGIs increased with age, the variance explained by RVBs *decreased* with age. Thus, these opposing directions rule out classical measurement error as driving these patterns of associations.

In our original submission, we showed that we could replicate the age interaction using school grades at ages 11 and 14 (Extended Data Figures 2, 4 and 8), which have very low attrition. We have now also replicated it in a new set of analyses where we only considered IQ at ages 8 and 16 (described in a new Supplementary Note 8), which should also have minimal differences in measurement error[11]. We additionally conducted several robustness analyses to assess the impact of attrition on the observed interaction effects and show that the attrition patterns are highly unlikely to induce an artefactual interaction where none exists, as described further below.

We added the following text to the Discussion to clarify the above explanation [line 383]:

"If the test reliability differed between the tests used at different ages, this may have induced spurious interaction effects between the genetic scores and age. However, this does not seem a likely scenario given that we replicate trends in other cohorts and with other cognitive performance measures, and we see age interaction effects in opposite directions for common versus rare variants for the same trait; if the only change across development were decreasing measurement error with age, we would expect to the association for both genetic scores to increase with age."

Attrition. If I understand findings properly, rare variants may substantially reduce IQ and therefore increase variance in the lower tail of IQ and lead to stronger genetic effects in that tail of the distribution. However, I find quite puzzling that the size of effect would attenuate with age for rare variants? As per the authors' own discussion of the Matthew effect, initial individual differences in ability tend to compound over time, so, if anything, we should expect a strengthening of the effects. And there does not seem to be any good reason why this effect would only exist for phenotypic individual differences that are due to common variants vs rare variants.

We agree that this is indeed a counterintuitive finding in light of the Matthew effect, though we note it is not necessarily inconsistent with it, as the effect may very well display quantile dependency and may not hold under certain conditions. It may be that the quantile-dependent rare variant effect attenuation is due to both social and biological mechanisms. We have added the following text to the Discussion to explore these:

"We found that the effect of parental EA on average IQ does not change across development, whereas the direct effect of EA-associated common variants increases (Figure 4), suggesting a cumulative influence of the latter (Supplementary Discussion). In contrast, the effects of both rare damaging variants and perinatal factors on cognitive ability attenuate as children age (Figure 4). *This may be due to acute, time-limited effects during early neurodevelopment that can be attenuated by later plasticity, potentially in response to environmental influences. There may be social and/or biological explanations for our observation that rare variant effects attenuate with age whereas common variants effects do not. For example, unlike common variants, rare damaging variants associated with neurodevelopmental disorders often also cause congenital anomalies of other organ systems; it may be that, in a subset of children with such variants, some of the cognitive delay is due to early-life physical health challenges (e.g., hospital admission for congenital heart disease, vision or hearing impairments) which resolve as children recover and receive targeted interventions. If this is true, one might predict that the attenuation of rare variant effects on cognition with age will be strongest in children from less deprived households, which could be tested in larger datasets. Another important difference between the rare and common variant-based scores we have considered is that, in any given individual, the RVB will be heavily driven by one or a small number of genes, whereas the PGI is composed of the small effects of thousands of genes. It may be that, for at least a subset of these single genes impacted by the rare variant/s, there are biological compensatory mechanisms that can attenuate the deleterious effect across development.*"

The authors mention some sort of compensation to explain this change: "due to acute, time-limited effects during early neurodevelopment that can be attenuated by later plasticity, potentially in response to environmental influence". If indirect genetic effects capture some of those environmental effects (i.e. if they are not entirely pop strat and assort mating), shouldn't they index this type of

environmental compensation? Can the authors discuss this point and to which extent their findings of indirect effects are consistent or not with compensation occurring with age (e.g. increasing indirect effects with age?).

We thank the reviewer for this interesting thought, that if environmental influences are changing with age and driving the changing effects of genetics, this should be reflected by changes in the indirect genetic effects with age. This is an appealing idea but we believe it may not be correct for two reasons. Firstly, these environmental influences could originate from non-parental sources, including during schooling, so be uncorrelated with the parents' genetics. Secondly, due to the limitations of the trio model, we cannot separate true indirect genetic effects from confounders such as assortative mating and population stratification - we can only estimate the association between non-transmitted alleles (which may reflect any or all of these factors) and the child's phenotype. Thus, changes in the non-transmitted coefficients with age may not necessarily reflect changes in indirect genetic effects. Having said that, we did observe nominally significant, positive age interactions between parental PGI_{NonCog} and IQ, which may possibly be indexing indirect genetic effects influencing the environment. However, given the caveats mentioned above, we do not wish to make any firm claims regarding the precise mechanisms driving any compensation for genetic effects with time.

Instead, a technical artefact like attrition may explain those findings (as I assume there is no excess mortality in those children?). Attrition is typically (much) higher for children with difficulties (parents probably have other things to do than to respond to questionnaires). We can imagine that children with rare variants substantially affecting their development, might be more susceptible to attrition. And if there was such attrition, we would lose variance in that low tail region the authors focus on, as well as probably reduce the size of the genetic association. And given that these are the individuals at the end of the distribution, loosing just a few might make a substantial difference (analogous to outliers). This is all the more important as the N at age 4 is a small fraction of what is available at ages and then you have more classical attrition between 8 and 16.

We thank the reviewer for this important comment. We have now included analyses to characterise the nature of the nonrandom missingness we observed in ALSPAC across ages. These are now described in Supplementary Note 1, reproduced here:

"To characterize the extent of nonrandom missingness among children with at least one IQ measurement, we compared the distribution of PGIs and RVBs among the different combinations of missingness by coding each individual's missingness pattern as categorical variable (e.g., "IQ measured only age 8", "IQ measured at age 4 and 8", etc.). We specifically assessed nonrandom missingness with respect to the genetic scores since collider bias[12] would only be induced if the variable of interest (in this case PGIs and RVB) is associated with ascertainment. We found significant associations between the IQ missingness pattern and all PGIs ($p = 1x10^{-16}$, $9x10^{-7}$, and $7x10^{-5}$ for PGI_{EA}, PGI_{Cog}, and PGI_{NonCog}, respectively, ANOVA). However, the variance explained in the PGIs by IQ missingness pattern was minimal, explaining 1.9%, 0.75%, and 0.55% PGI_{EA}, PGI_{Cog}, and PGI_{NonCog}, respectively. In general, those with fewer tests had lower mean PGIs than those with more than one test completed, and those with only age 8 measurements had the lowest mean PGI_{EA} and PGI_{Cog} (Supplementary Table 10). In contrast, we found no association between missingness pattern and any RVB ($p = 0.064$, 0.76, and 0.32, for RVB_{pLoF}, $RVB_{Missense}$, $RVB_{Synonymous}$, respectively, approximative K-Sample Fisher-Pitman permutation test for difference in means). However, it is possible that we were underpowered to detect associations between RVB and missingness due to the relatively low variance explained by RVB on relevant phenotypes. These results suggest that, although subtle, nonrandom missingness may influence our findings via collider bias especially in

the pre-imputation analyses. To estimate the extent of this bias, we conducted various simulation studies and robustness analyses (e.g., Supplementary Note 8).
”

We now address the question about whether the pattern of attrition could have driven the observed differential genetic effects across development. Firstly, we would like to draw the reviewer’s attention to our initial results in Extended Data Figures 2,4 and 8A; here, we recapitulated evidence for the age-interactions using school grades measured at ages 11 and 14, where there was minimal drop-out between time points (<3% individuals).

To add to this, we conducted several robustness analyses to address the reviewer’s point. These have been added to the new Supplementary Note 8, with key results shown in Supplementary Table 11, and methods described in Supplementary Methods. We reproduce those here for convenience:

Supplementary Note 8: Assessing the robustness of age interaction findings to non-random attrition

As there is nonrandom missingness in the ascertainment of IQ measures (Supplementary Note 1), we sought to assess the extent to which it may bias our pre-imputation results. To do so, we conducted several simulation studies and robustness analyses.

In the first simulation study (the “constant effects model”, see Supplementary Methods), we simulated IQ using RVBpLoF and PGIEA at three time points using the true genetic scores of individuals. We modelled each IQ variable as a standard normally distributed variable, having a partial correlation with PGIEA of 0.3 and with RVBpLoF of -0.1 (both constant with age), to mimic the effect sizes we empirically observed. We then masked individual IQ scores to mirror exactly the missingness pattern observed in the real data. Following masking, we then restandardized each IQ measure to have a mean of 0 and standard deviation of 1 and fit the mixed-effect regression models as we did in the main analyses in Figures 1 and 2. We did not find any evidence to suggest that, under this data-generating model, the age interaction with PGI or RVB (which should be zero here) would be estimated with meaningful bias given the observed pattern of missingness (Table S11).

In the second simulation study, we used the exact same parameters as before though we simulated a scenario where the effect of PGIEA increased linearly with age at a rate of 0.1 per 10 years to mimic the empirical observation. We again found minimal evidence for bias (Table S11), and found we had 58.3% power to detect the PGIEA age interaction in the pre-imputation sample.

Given that we observed RVB associations at the 5th percentile attenuating with age, one might be concerned that a higher rate of attrition at the lower end of the IQ distribution could be producing an artefactual age interaction. To assess this possibility, we simulated a scenario where there is a strong effect of RVB on the lower half of the IQ distribution that is constant ages. Specifically, we modified the prior simulation to make RVBpLoF have an effect of -.2 but only in individuals whose IQ value, prior to considering the RVB effect, is in the lower half of the IQ distribution. We found little evidence that the missingness pattern induced meaningful bias in either the PGIEA or the RVBpLoF interaction term. On average, the RVBpLoF x age interaction term was slightly upward biased, but the magnitude of the bias was much lower than the empirically estimated effect sizes; in the simulations, the average RVBpLoF x age interaction was 0.009 per 10 years, whereas the empirically observed RVBpLoF x age interaction pre-imputation was 0.063 (Table S11). Additionally, false positive rates were well-controlled, as is evidenced by the fact that only 2% of simulations had significant RVBpLoF

x age interactions. Thus, it is unlikely that, if there were true, quantile-dependent effects of RVBpLoF but no age interaction effect, attrition alone could have produced the age interaction we observed.

Returning to the real data, we also refitted the mixed-effects models after excluding age 4 as that was the age with the highest missingness (Extended Data Figure 1). We found that the point estimates were largely consistent with those obtained when including age 4 both pre- and post-imputation, with most estimates remaining statistically significant despite reduced power (Table S12). Of the nine nominally significant age interactions we observed when including all three ages, two became non-significant when restricting to ages 8 and 16, which seems likely to be due to the loss of power. These results are consistent with the replication of the age interactions we observed in school grades which were ascertained at ages 11 and 14 (Extended Data Figure 2).

Collectively, these results suggest it is highly unlikely that the differential age interaction trends observed for PGIs and RVBs are due to the ascertainment patterns of IQ measures but rather likely reflect true differential trajectories of effects across development.

Supplementary Methods: Sensitivity analyses using simulated IQ measures

We carried out three Monte Carlo simulation scenarios to evaluate the impact of nonrandom missingness and age-dependent genetic effects on the estimated associations between IQ and two predictors, PGIEA and RVBpLoF. All simulations used the real missing data patterns observed on IQ at ages 4, 8 and 16 in ALSPAC.

1. Constant effects across age (constant effects model)

For each subject, we standardized PGIEA and RVBpLoF to mean=0 and SD=1. At each age, simulated IQ = 0.30xPGI + (–0.10)xRVB + ε, where $\varepsilon \sim N(0, \sigma^2)$ and σ^2 was chosen so that Var(IQ)=1. The effect sizes were chosen to approximate those observed empirically (Figure 1 and 2). The observed missingness mask was then applied, and estimated the mixed-effects regressions as done previously:

$$IQ_{i,t} \sim age_t + PGI_{EA,i} + RVB_{pLoF,i} + age_{i,t}*PGI_{EA,i} + age_{i,t}*RVB_{pLoF,i} + PC1_i + .. + PC10_i + sex_i + C_i$$

2. Increasing PGI effect with age (Positive PGIEA x age interaction model)

This was the same model as above, except that the PGI coefficient grew linearly by 0.01 per year after age 4:
IQ = (0.30 + 0.01x[age - 4])xPGI + (-0.10)xRVB + ε.

3. Age-varying PGI plus heterogenous RVB effect (Positive PGIEA x age interaction + RVBpLoF heterogenous effect)

Building on scenario 2, we first computed a subject-level “baseline age 4 IQ” = 0.30xPGI + v where ($v \sim N(0, \sigma^2)$), then identified the bottom 50% of that distribution. Then, only those in the lower half received the RVB effect (–0.20xRVB); all others had no RVB contribution:

IQ = (0.30 + 0.01x[age - 4])xPGI + (-0.20)xRVB + v if baseline age 4 IQ below 50th percentile
IQ = (0.30 + 0.01x[age - 4])xPGI + v otherwise

The PGI effect continued to increase by 0.01 per year. This was intended to simulate an extreme case scenario, where the effects of RVB were only present in the lowest half of the baseline scores, though we note we empirically observed a much more subtle gradation of effects.

We repeated each scenario 1,000 times, refitting the mixed model to each simulated dataset to obtain distributions of the PGIEA × age and RVBpLoF × age slopes under each data-generating process.

Characterizing attrition. The question of missingness and attrition is thus particularly important for the interpretation of results. For that reason, I think that additional information should be included in the manuscript rather than just in the supplement (line 110). At the very least to clarify how many individuals were imputed. The authors say: "Our primary analyses were based on measures of IQ from ALSPAC that were collected at ages 4 (n=1,012), 8 (n=7,347), and 16 (n=5,270)". Can you please clarify explicitly in the manuscript:

1. What is the rule for imputation, I assume at least one IQ measure across the 3 ages;
2. What is the final sample size after imputation, which I assume will be > 7347 because of imperfect overlap across ages.
3. Specify explicitly for the reader how many IQ values were imputed at the different ages (i.e; basically total n for imputed sample minus observed values at each age).
4. Provide in the manuscript more descriptives to characterize attrition, e.g. is there a difference in average genetic burden scores at the different time points? Any difference in polygenic scores?

We thank the reviewer for this point. We have now added in the start of the Results section a more in-depth explanation of the imputation and attrition as follows:

"We observed that IQ missingness was nonrandom with respect to individuals' polygenic indices (PGIs) for educational attainment, particularly among individuals with measures at age 4 and 16 (Supplementary Note 1, Extended Data Figure 1C, Supplementary Table 10). This suggests subtle biases in which individuals did the IQ tests at the different ages, addressed further below. Since phenotypic missingness can induce ascertainment biases and reduce power, we imputed missing IQ values across ages among participants who had at least one IQ measure at any time point (n=8,207) (Supplementary Note 1; Extended Data Figure 1). Briefly, we imputed missing IQ measures using SoftImpute37, which leverages correlations with other variables. These included cognitive and behavioral tests, birthweight, and demographic variables (see Methods)."

As noted above, we have included a section in Supplementary Note 1 about differences in average polygenic indices and rare variant burden scores for individuals with observed IQ at different ages. We have also added a new panel C to the Extended Data Figure 1 (see below; data underlying the figure in Supplementary Table 10) to show the average polygenic scores and average rare variant burden scores in the participants with observed versus imputed IQ values at each age. We observed relatively subtle differences in mean PGIs between people with observed versus imputed IQ across genetic measures, which are not significantly different at any age. In the legend of this figure, we give the final sample size used pre- and post-imputation and the number of imputed individuals at each age. Recognizing the nonrandom missingness with respect to PGIs especially (Supplementary Note 1), we additionally added several robustness analyses in a new Supplementary Note 8 to assess the impact of attrition on inferences made on our mixed-effects modelling as described in above.

Finally, we have now also simplified the main text throughout, making it easier to track so that sample sizes for different analyses are easier to track. For example, the first sentence in the section on

common variants begins with with "We first considered the heritabilities and genetic correlations of IQ measured at different ages in ALSPAC, using **6,495** unrelated children with SNP genotype data, genetically-inferred European ancestry and observed or imputed IQ values." We later specify for the PGI trio analysis, "We repeated the above analyses adding parental PGIs as covariates in a subset of **4,968** unrelated parent-offspring trios" and for the RVB analysis we now clarify "We first used these to assess the associations between IQ and rare variant burden (RVB) in ALSPAC (n=**6,495**)." followed by "We then estimated the direct genetic effects of deleterious rare variants on IQ across development in **958** unrelated exome-sequenced trios" (bolding added for clarity here, but not in the text). We also included the sample sizes in the figures themselves, in their legends, and indicate the number of observed or imputed individuals in the legend of Extended data Figure 1 (See above).

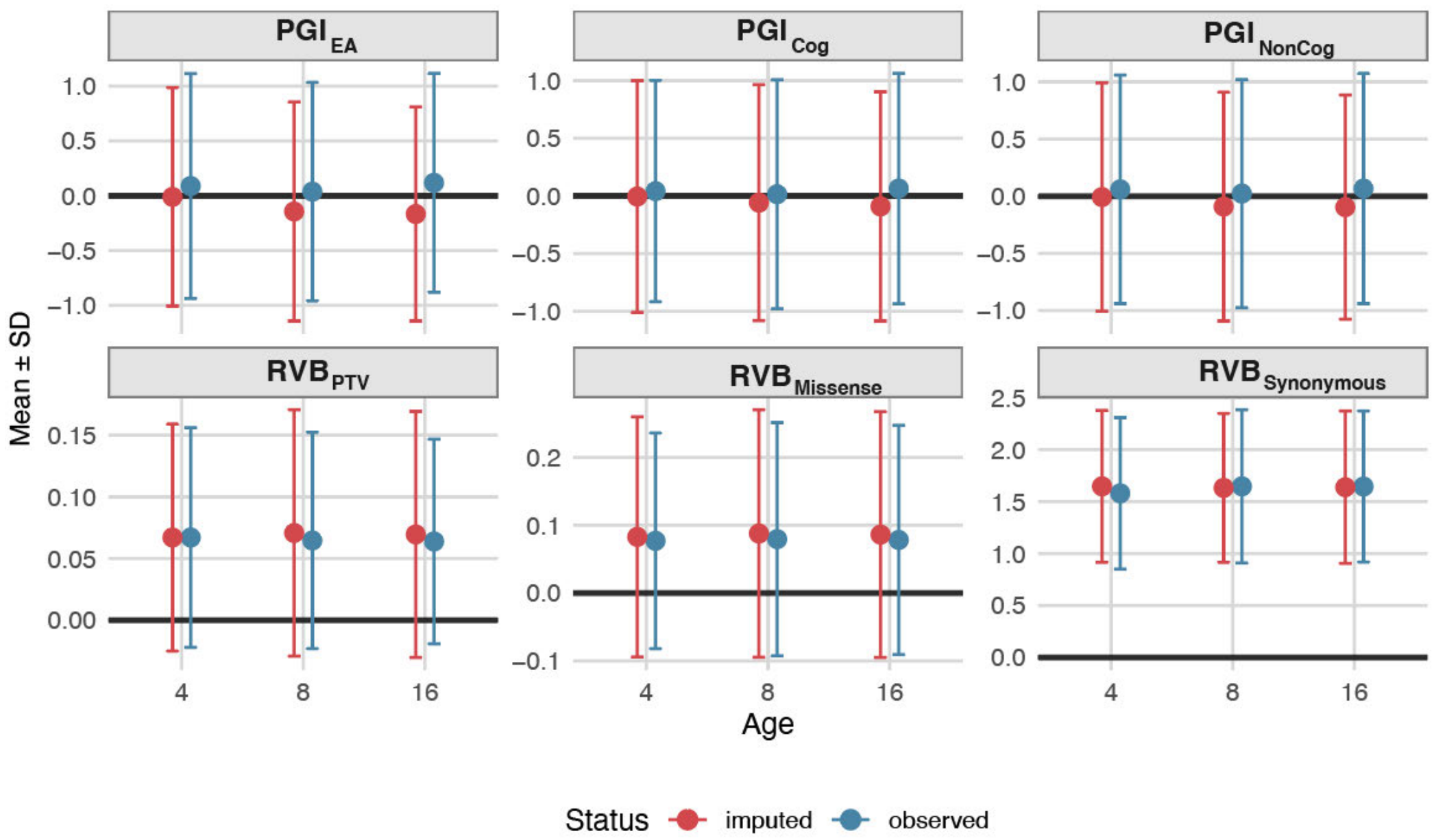

New Extended Data Figure Panel 1C. Mean and standard deviation of genetic scores for individuals where an IQ measurement was either observed (red) or imputed (blue) at ages 4 (n observed = 701, imputed = 5794), 8 (5134, 1361), and 16 (3774, 2721) among those with genetic data.

In brief, I would expect the authors to better characterize attrition and describe imputation in the manuscript and acknowledge more in depth the possibility that some of the core findings might results from such technical artefacts such as measurement error and attrition.

Minor:
L 138: sentence starting by "Importantly, we found that common…" is long and could be clearer.

We have now rewritten this sentence: "Thus, our results suggest that common variants ascertained for their association with cognitive performance and final educational attainment in adults explain increasingly more variance in IQ as children age."

Can't see titles for figures.

We have now added and bolded figure titles.

Reviewer #2 (Remarks to the Author):

This paper by Maawsky and colleagues pursues a very topical idea in neurodevelopmental genetics, namely that the genetic contribution to phenotypic variation depends not only on genetic variation, but expression across the lifespan. In particular, dynamic changes in gene expression chimes with the idea of sensitive periods of development for language and cognition and for neurodevelopmental disorders. In the complex series of analysis presented in this paper, based on public datasets (ALSPAC, UKB) the authors show that the effects of common and rare variants on cognition vary across childhood. Specifically, they report evidence that the effects of common variants strengthen with age, whereas as the effects of rare variants attenuate with age. Importantly, they also provide evidence that these dynamic effects also depend on position in the phenotypic distribution, with genetic effects becoming either weaker or more penetrant towards either end of the distribution.

By considering the two axis of time (aim 1) and relative phenotypic distribution position (aim 2), the authors provide important evidence of the dynamic effects of common and rare genetic variation, advancing on previous studies in this area. This is important, for example, because of the studies relevance to understanding the incomplete penetrance of rare (deleterious) variants.

The authors also include (aim 3) consideration of a number of factors that are likely to impact on these relationships, including environmental factors (e.g. parental education) and differences in phenotypic expression between those with a familial relationships (e.g. to comment on 'genetic nurture'). While this is a strength of the paper in my view, this it also made the results section of the paper long and quite challenging to read. This was further complicated by multiple phenotypes being used to index cognition and multiple samples. This complexity is illustrated e.g. in the 8-panel figure (Fig 1 and 2) and 10-panel figures (fig 3) which are difficult to interpret. I suspect these could be simplified, for example putting the non-imputed panels into supp data.

We thank the reviewer for these suggestions. We have now reduced the length of the final results section (addressing aim 3) by about a third, in particular the part about the quantile regression, and we have moved the previous Figure 4B panel illustrating the latter analysis to Figure S10. The results from this analysis are described in more detail in Supplementary Note 6. We have also simplified Figures 1 and 2, limiting the main text figures to post-imputation analyses, and moving the pre- and post-imputation analysis figures to the Supplementary Figures. We have also moved panel A from Figure 3 to Extended Data Figure 6, and rewrote the caption to more clearly describe the schematic used to explain quantile regression. Lastly, we have reduced the complexity and amount of text throughout, moving several minor points to the supplementary sections.

The authors discuss a number of factors that might explain the increase of heritability of cognitive performance up to age 18 (including genetic innovation, amplification, and the compounding effects of educational experience). Surprisingly the authors don't explicitly discuss dynamic changes in gene expression or the pattern of differential gene expression at different stages in development. Neither do they link their findings to likely neurobiological changes in brain structure and function(e.g. synaptic pruning).

The reviewer makes a very interesting suggestion. We suspect that what they're asking is for us to test whether the increasing heritability with age is driven by changing gene expression patterns which contribute to neurobiological changes. This is an appealing suggestion that seems very plausible from a biological point of view. However, if this were true, we would probably expect the relative effect sizes of SNPs affecting different biological pathways/expression modules to change with age, which is inconsistent with our observation that the common variant-based genetic correlations between the ages are not significantly different from 1 (Table S2). Having said that, the error bars are such that we cannot rule out that the true genetic correlations are slightly less than 1 (e.g. r_g in IQ between age 4 and age 16 is 0.96 with 95% confidence interval [0.913,1.007]), although our estimates suggest the true r_g is likely still very high (>0.9). Hence it's certainly possible that the phenomenon the reviewer is hinting at may be at play.

In theory, we could explore this using partitioned heritability methods to test whether different gene expression modules (e.g. genes peaking in expression in early childhood versus adolescence from Brainspan[13]) or in particular neurodevelopmental pathways (e.g., synaptic-pruning vs. myelination) contribute disproportionately to the rising heritability curve. However, given that the age interaction effects estimated using genome wide analyses are relatively modest (age interaction using GREML p=0.053, age interaction using PGIs in the range of 10^{-2}-10^{-4} depending on the analysis), it is unlikely that analyses stratifying the effects further will have sufficient power. It will be extremely interesting to undertake these analyses in further if we can collect much larger sample sizes of children with longitudinal cognitive measures, but we don't feel that such analyses would be useful at our current modest sample size.

We have added the following sentence to the final paragraph of the Discussion to reflect the reviewer's suggestion: "Larger cohorts would allow us to test whether particular neurobiological processes disproportionately contribute to the increasing heritability of cognitive ability e.g. using partitioned heritability approaches[59]."

Finally, the authors conclude that the future work suggested by their study should be to identify children at risk of poor clinical outcomes. I did not find this a compelling conclusion. Related to the point above, the next steps would I believe be about link these findings to longitudinal changes in brain development. If the authors do feel that their study has implications for genetic screening, these need to be made more explicit and concrete than the conjectures currently offered.

We have now removed the point about identifying children at risk of poor outcomes from the final paragraph of the Discussion and replaced it with this sentence: "Larger cohorts would allow us to test whether particular neurobiological processes disproportionately contribute to the increasing heritability of cognitive ability e.g. using partitioned heritability approaches[59]."

Reviewer #3 (Remarks to the Author):

In this paper, Malawsky et al. present a detailed analysis of common and rare-variant genetic influences on cognitive performance across development, using data from the ALSPAC, MCS, and UK Biobank studies. The primary genetic tool for common variant analysis is polygenic indices (PGIs) for EA, EA-Cog, and EANonCog. Rare variant burden (RVB), weighted using RGC-ME (Ref. 46), is used for rare variant analysis. The paper is well-structured and presents a clear and logical narrative; however, I have several major comments and concerns for the authors to consider.

(1) “Genetic effects” is a tricky term to use in the title and throughout the main text. In the context of PGI analysis for common variants, the authors interpret the associations between EA-PGI (or EA-Cog and EANonCog) and IQ as “population effects” from the PGI and IQ, with the “direct effects” inferred after adjusting for parental genetic scores. However, these are merely associations derived from mixed-effects models and do not necessarily imply a directional effect from EA-PGI to IQ, even after adjusting for parental genetic scores. In many genetic studies, “genetic effects” typically refer to causal relationships from genes or variants to phenotypes, which may lead to confusion if it is used to interpret PGI-based associations. A more precise framing of these associations could improve clarity.

We thank the reviewer for the opportunity to clarify our interpretation of polygenic score associations. We have changed the title to replace "effects" with "associations" to prevent misinterpretation and better reflect the observational nature of our findings. While we appreciate the reviewer's concern about potential confusion, we prefer to retain the standard terminology of "population genetic effects" and "direct genetic effects" in the main text, as this language is well-established in the human genetics literature and is used consistently in papers, including in recent publications in Nature Human Behaviour.[14–16] To ensure clarity, we have provided explicit definitions of these terms in Box 1, and added a sentence to this explaining why we use the term “genetic effect”: “We note that our use of the term “genetic effect” does not (necessarily) imply causality, but we have used this terminology as it is a standard way of describing genetic associations in the field”. Additionally, we have carefully reviewed and removed all claims of causality from the manuscript, ensuring that our interpretation focuses appropriately on the associational nature of the findings. We believe this approach balances methodological precision with consistency within the field, while clearly communicating the limitations of our observational design to readers. If the reviewer or editor feels strongly, we could change this terminology but we believe this would lead to confusion due to inconsistency with other papers.

(2) One key finding is that the association between EA-PGI and IQ varies across the three developmental time points. Since EA-PGIs are derived from GWAS on adult education attainment (EA), their predictive power may be stronger for age 16 IQ than for earlier ages, simply due to the nature of the discovery GWAS sample. As the authors briefly acknowledged (Line 384), there are multiple potential explanations for this pattern, beyond the “changing genetic effects”. For example, if phenotypic IQ consists of both a genetic component (IQ-G, which is heritable) and a non-genetic component (IQ-nonG), then the EA-PGI and IQ association could be decomposed to “EA-PGI and IQ-G” (e.g., genetic correlation between the two phenotypes) and “EA-PGI and IQ-nonG” (e.g., potential confounding effects). Changes in either of these parts could drive changes in the observed EA-PGI and IQ total association, making it difficult to interpret these results solely as shifts in “genetic effects” of EA-PGI on IQ.

We appreciate the reviewer’s thoughtful comments and agree that several mechanisms could, in principle, generate the developmental pattern we report. However, converging evidence indicates that age- related increases in the PGI_{EA} association most plausibly reflect genuine changes in the strength of genetic effects rather than biases in the discovery GWAS sample or shifts in measurement or confounding processes.

1. An adult- based discovery GWAS could only explain stronger prediction at age 16 if genetic correlations between adult EA and early childhood IQ were lower than between adult EA and

adolescent IQ. Our data show uniformly high genetic correlations that do not vary significantly with age (Table S3), rendering this explanation unlikely. We make this point in the second paragraph of the Discussion (lines 307): "In theory, the increasing effect of polygenic indices for educational attainment and adult cognitive performance with age could simply be because the underlying SNP effects have been estimated on adult phenotypes which are better correlated with IQ in later childhood than early childhood54,55. However, since common variant genetic correlations between IQ measured at the different ages were not significantly different from 1 (Table S2), it is more likely that this effect is driven by a true increase in common variant heritability56 (Supplementary Discussion)."

2. IQ distributions are age- standardised so that total phenotypic variance is fixed at each wave. For the proportion of variance attributed to genetics to increase without a corresponding rise in the absolute genetic effect size, total variance would have to decrease, which contradicts the standardisation constraint. The genetic contribution must therefore increase with age for the observed effects to hold.

3. In the within-family analyses, the variance in IQ explained by the PGI continues to increase from age 4 to 16. If environmental confounding were driving the pattern, the interaction would weaken once between-family influences are removed. However, we see no such attenuation of the PGI-by-age interactions after controlling for parental PGIs.

Taken together, these findings support amplification of genetic signals captured by the EA- PGI rather than static effects masked by changing environments or measurement properties.

(3) Another key finding is the difference in the role of common versus rare variants. I feel this may not be entirely surprising given their distinct definitions and estimation methods. RVB scores are additive burden scores weighted by evolutionary constraint/negative selection, making them conceptually closer to traditionally defined "genetic effects". Therefore, it is unclear whether the changing effect patterns of these burden scores are comparable to the changing patterns of PGI-based associations with IQ, as they may measure fundamentally different concepts. Comparing PGI association results between two phenotypes to RVB results of one phenotype may be conceptually similar to comparing apples to oranges, the interpretation may require careful consideration.

The reviewer makes a reasonable point. We appreciate the opportunity to clarify why we compare the age-specific associations of the polygenic index (PGI) built from common variants with the rare-variant burden (RVB) score, although the two scores are constructed in different ways. Both scores are constructed to model an individual's additive genetic liability to IQ. The PGI does so in the part of the allele-frequency spectrum where single-variant effect estimates are reliable, while the RVB score aggregates the contributions of very low-frequency alleles whose individual effects cannot yet be estimated accurately with available sample sizes. Indeed, the effect sizes of rare PTVs on cognition when aggregated by gene can still not be estimated precisely using the largest available cohort with cognitive data (UK Biobank). We note that evolutionary constraint against heterozygous PTVs in a gene (i.e. the s_{het} score) is linearly correlated with the effect of those PTVs effect on fluid intelligence in UK Biobank (see new Extended Data Figure 3 below).[17] This suggests that using s_{het} as a weight for all rare PTVs in a gene is going to be reasonably correlated with a rare variant-based PGI constructed using explicitly-estimated variant-specific weights. Hence, we believe it is still legitimate to compare PGI association results with those observed for RVB as currently constructed.

We added the following figure as Extended Data Figure 3 and the following to the Limitations paragraph of the Discussion:

“Thirdly, the genetic scores we have used to capture the effects of common and rare variants under-estimate the total liability conferred by these variants *and were constructed using different methodologies, where we relied on selection coefficients as gene-level weights as opposed to the phenotype-driven association studies used for the PGIs. However, we note that there is a strong, linear relationship between a gene’s selection coefficient and the association between PTVs in the gene and fluid intelligence in UK Biobank[17] (Extended Data Figure 12), suggesting that using the more precise measurement afforded by the selection coefficient is a reasonable weighting strategy.”*

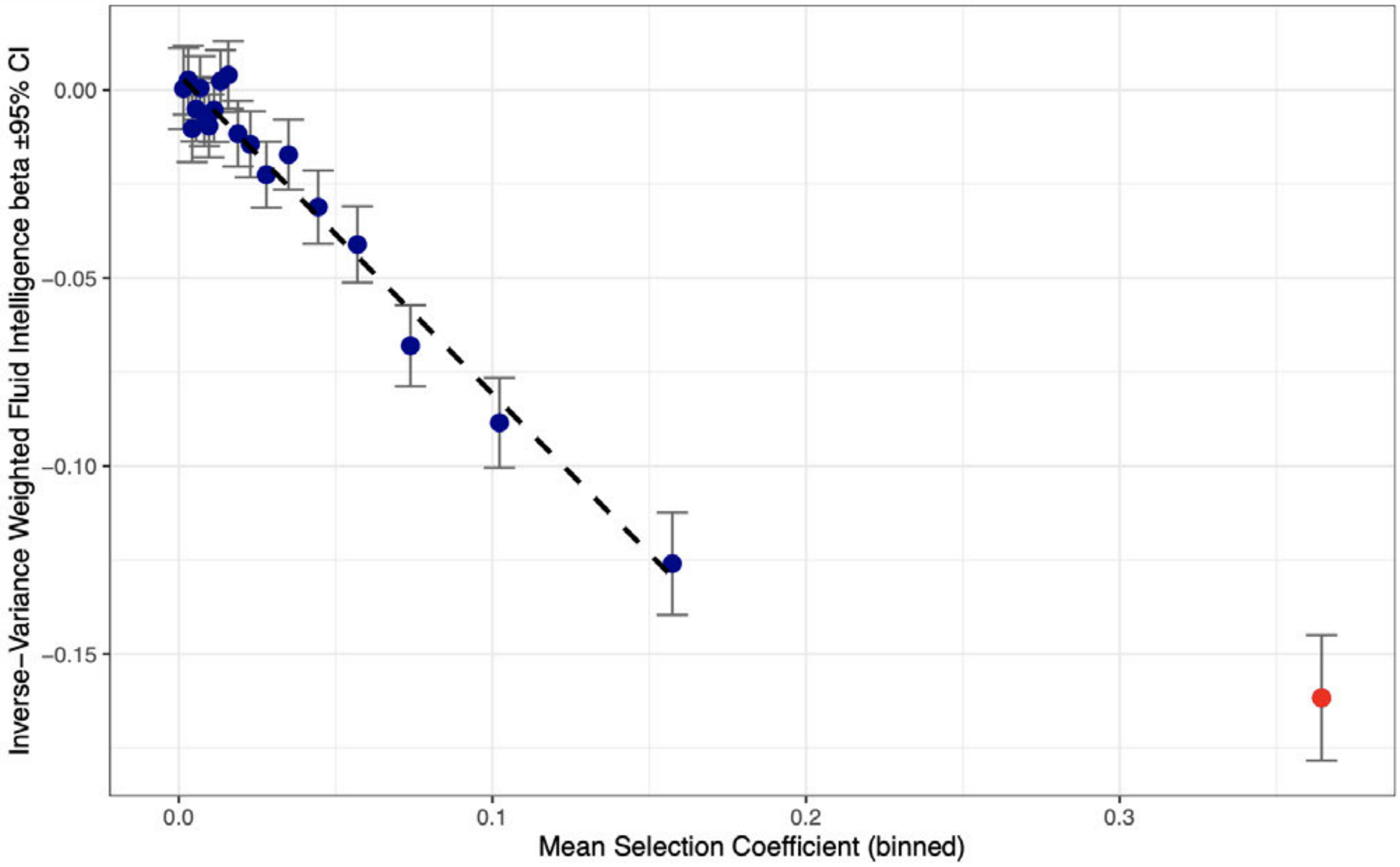

Extended Data Figure 3. Association between gene-level selection coefficients and predicted loss-of-function (pLoF) gene burden effect sizes on fluid intelligence. Shown are inverse-variance-weighted mean effect size estimates and 95% CI bands computed in 20 equal-count bins of genes. The bins were constructed using the gene selection coefficient estimates used as weights in the RVB measures, with each point representing one bin and the x-axis position being the mean selection coefficient for the genes in the given bin. The red dot marks the highest bin (excluded from the dashed regression line as it is an outlier), and the black dashed line is the best-fit linear trend across the remaining 19 bins. The red dot is likely an outlier because pLoF variants in highly constrained genes found in UK Biobank are enriched for technical or annotation artefacts[18], reducing their average effect size on cognition.

(4) A more direct approach to assessing common vs. rare variant effects on cognitive performance would be to estimate variant/gene-level genetic effects in a large cohort and compare them. However, current sample sizes may be too limited to allow for such direct comparisons, especially for rare variant analysis. Instead, here the authors compare them using PGI and RVB tools, but as outlined in previous comments, the interpretation of their differences as “different genetic effects” may be challenging.

We thank the reviewer for this suggestion. In response to this, we attempted to construct a rare variant score using gene burden effect size estimates from the paper by Chen et al. (Nature Genetics,

2023), who carried out an exome-wide association study on fluid intelligence in UK Biobank. To reduce the noise (given the limited power to estimate rare variant-based gene burden effects in UKB), we included only the 115 genes in which rare protein-truncating variants were associated with fluid intelligence with $p<0.005$ and beta<0 (assuming that genes with beta>0 would be false positives). We then used the effect sizes of these genes from Chen et al. to construct a weighted sum of rare protein-truncating variants in ALSPAC individuals. Hence, this is analogous to a common variant-based PGI except that the effects are estimated per gene rather than per variant. We observed much weaker associations using this scoring approach (main effect on IQ post-imputation = -0.038, p = 0.0012 ; pre-imputation = -0.050, p = 0.0027) compared to the approach we used in the paper, where main effects were more than twice as large. The age interaction effects were positive, as in our main results, but not significant, likely due to reduced power (per 10 years interaction effect post-imputation = 0.0075, p = 0.249 ; pre-imputation = 0.0026, p = 0.876).

Given that this UKB-derived score is clearly underpowered compared to the s_{het}-based RVB score we use in the main paper, we have not added this analysis in the manuscript although we could do if the editor or reviewers would like us to.

1. Ni, G., Moser, G., Schizophrenia Working Group of the Psychiatric Genomics Consortium, Wray, N. R. & Lee, S. H. Estimation of genetic correlation via linkage disequilibrium score regression and genomic restricted maximum likelihood. *Am. J. Hum. Genet.* **102**, 1185–1194 (2018).
2. Bulik-Sullivan, B. K. *et al.* LD Score regression distinguishes confounding from polygenicity in genome-wide association studies. *Nat. Genet.* **47**, 291–295 (2015).
3. Churchhouse, C. Insights from estimates of SNP-heritability for >2,000 traits and disorders in UK Biobank. *Neale lab* https://www.nealelab.is/blog/2017/9/20/insights-from-estimates-of-snp-heritability-for-2000-traits-and-disorders-in-uk-biobank (2017).
4. Markel, G. *et al.* Nature, nurture, and socioeconomic outcomes: New evidence from sib pairs and molecular genetic data. *Social Science Research Network* (2025) doi:10.2139/ssrn.5225447.
5. Durvasula, A. & Price, A. L. Distinct explanations underlie gene-environment interactions in the UK Biobank. *Am. J. Hum. Genet.* **112**, 644–658 (2025).
6. Trzaskowski, M., Yang, J., Visscher, P. M. & Plomin, R. DNA evidence for strong genetic stability and increasing heritability of intelligence from age 7 to 12. *Mol. Psychiatry* **19**, 380–384 (2014).

7. Haworth, C. M. A. *et al.* The heritability of general cognitive ability increases linearly from childhood to young adulthood. *Mol. Psychiatry* **15**, 1112–1120 (2010).
8. Spence, J. P. *et al.* Specificity, length, and luck: How genes are prioritized by rare and common variant association studies. *Genomics* (2024).
9. Wright, S. N., Yang, J. & Ideker, T. Common and rare genetic variants show network convergence for a majority of human traits. *medRxiv* 2025.06.27.25330419 (2025) doi:10.1101/2025.06.27.25330419.
10. Eika, L., Mogstad, M. & Zafar, B. Educational assortative mating and household income inequality. *J. Polit. Econ.* **127**, 2795–2835 (2019).
11. Breit, M., Scherrer, V., Tucker-Drob, E. M. & Preckel, F. The stability of cognitive abilities: A meta-analytic review of longitudinal studies. *Psychol. Bull.* **150**, 399–439 (2024).
12. Munafò, M. R., Tilling, K., Taylor, A. E., Evans, D. M. & Davey Smith, G. Collider scope: when selection bias can substantially influence observed associations. *Int. J. Epidemiol.* **47**, 226–235 (2018).
13. Kang, H. J. *et al.* Spatio-temporal transcriptome of the human brain. *Nature* **478**, 483–489 (2011).
14. Akimova, E. T., Wolfram, T., Ding, X., Tropf, F. C. & Mills, M. C. Polygenic prediction of occupational status GWAS elucidates genetic and environmental interplay in intergenerational transmission, careers and health in UK Biobank. *Nat. Hum. Behav.* **9**, 391–405 (2025).
15. Malanchini, M. *et al.* Genetic associations between non-cognitive skills and academic achievement over development. *Nat. Hum. Behav.* **8**, 2034–2046 (2024).
16. Gui, A. *et al.* Genome-wide association meta-analysis of age at onset of walking in over 70,000 infants of European ancestry. *Nat. Hum. Behav.* 1–18 (2025).
17. van den Berg, D. M. *et al.* Imputation of fluid intelligence scores reduces ascertainment bias and increases power for analyses of common and rare variants. *medRxiv* (2025) doi:10.1101/2025.06.18.25329418.
18. MacArthur, D. G. *et al.* A systematic survey of loss-of-function variants in human protein-coding genes. *Science* **335**, 823–828 (2012).

Responses

Reviewer #1 (Remarks to the Author):

The authors have comprehensively addressed my comments and, even when they did not follow my recommendations, their arguments are appropriately justified.

We thank the reviewer for their time and constructive comments.

Reviewer #2 (Remarks to the Author):

The authors have adequately responded to my queries and are to be commended on an interesting manuscript which will contribute to the field.

We thank the reviewer for their time and constructive comments.

Reviewer #3 (Remarks to the Author):

(1) I agree that the term “genetic effects” is often used in the literature; however, in my view, its appropriateness largely depends on the context and the specific phenotype pairs being studied and thus needs careful consideration. In particular, for genetic associations between EA and IQ, the relationships can be very complex, and using the term “genetic effects” may be easily misleading when others interpret the findings.

We understand the reviewers' concern regarding the use of the term “genetic effects”. We have now gone through the manuscript and removed references to “genetic effects” in most instances, including in the title. After consultation with the editor, we have retained the terms “population effect”, “main effect”, “direct genetic effect” and “indirect genetic effect” to be consistent with previous literature (including recent papers in *Nature Human Behaviour*), but we have explicitly defined these in Box 1, making clear that these reflect associations rather than necessarily causal effects.

(2) I appreciate the authors’ efforts to clarify their reasoning regarding the varying association between EA-PGI and IQ. Their current argument, which combines multiple lines of evidence, is stronger than previous ones. I would encourage the authors to explicitly draw on these converging pieces of evidence when interpreting their findings in the main text. This would provide a more systematic explanation and justification for the age-related increases in EA-PGI and IQ association.

We are glad the reviewer found our arguments compelling. Due to space constraints in the main text, we have instead added these notions to the second paragraph of the section

“Implications and interpretation of our findings of increasing strength of PGI associations with age” in the Supplementary Discussion:
“One might posit that the increasing variance explained by PGIs with age could simply be due to a decrease in the environmental effects on IQ (i.e. the heritability could be increasing with age simply because environmental variance decreases), rather than the absolute genetic effect size actually changing. However, we note that since IQ was standardized within age, for the proportion of variance attributed to genetics to increase without a corresponding rise in the absolute genetic effect size, total variance would have to decrease, which contradicts the standardization constraint. The *absolute* genetic contribution must therefore increase with age for the observed effects to hold. Furthermore, we note that in the within-family analyses, the variance in IQ explained by the PGI continues to increase from age 4 to 16. If environmental confounding were driving the pattern, the interaction would attenuate once between-family influences are removed. However, we see no such attenuation of the PGI-by-age interactions after controlling for parental PGIs, suggesting the direct genetic effects are driving the increased genetic contribution with age.”

(3) I thank the authors for addressing my question about the “common/rare comparisons”. After reviewing the responses, I am afraid my original concerns still remain.
I am not fully convinced that the argument directly addresses my question: “Both scores are constructed to model an individual’s additive genetic liability to IQ. The PGI does so in the part of the allele-frequency spectrum where single-variant effect estimates are reliable...” In this study, the authors are using a PGI for EA, not IQ. The PGI therefore reflects additive genetic effects on EA, and the major analysis focuses on the genetic association strength between this EA-PGI and IQ, which is interpreted as common genetic effects on IQ. While the authors explain their use of the shet score in rare variant analysis by including the new Extended Data Figure 3, this does not explicitly address my question. My concerns about comparing the “EA-IQ genetic association pattern” with the RVB results remain. I find it difficult to support drawing strong (!) conclusions about differences in the roles of common versus rare variants based on the current evidence.

We thank the reviewer for their thoughtful comment. We apologise for our carelessness in claiming previously that the PGI_{EA} was constructed to model an individual’s additive genetic liability IQ, which is of course incorrect. We do note, however, that in our main analyses we used not only a PGI for EA, but also a PGI for the cognitive component of EA (from Demage et al., *Nature Genetics*, 2021), which is effectively a PGI for adult cognitive performance (with some caveats/nuances, of course), and that the results for these two PGIs were highly concordant. Nevertheless, we have now replaced mentions of the effects of “common variants” and of “rare variants” with references to associations with PGIs and RVBs, to avoid implying that we can necessarily make general conclusions about common versus rare variants *per se*. For example, we have removed the statement “In contrast to the results for common variants” (line 182) in the Results section on RVB results to avoid making a direct comparison with the PGI results. In addition, we have added the following caveat to the second paragraph of the Discussion: “Since the construction of the common variant-based PGIs and the RVBs differ, the constructs are technically not directly comparable, so one should be cautious about drawing strong conclusions about differences between their patterns of association.” (line 318). We have also added a point in the final paragraph of the Discussion about future work: “Larger cohorts would allow us to construct more comparable scores for rare versus common variants, allowing for more direct comparison of their associations.” (line 427)

(4) Thank you to the authors for their efforts in conducting new analyses. These results further support my original view that the current datasets may lack sufficient power to address this research question. However, this does not justify interpreting the current findings as evidence of “different genetic effects”.
While I appreciate the interesting research question this study seeks to address, I really find it difficult to get behind the concept of this comparison and support the way to interpret their difference, given the fundamental differences between “EA-IQ genetic associations” and RVB burden tests of IQ.

We thank the reviewer for their comment. We hope our response to related comment #3 above will satisfy them on this point.

Reviewer #3 (Remarks to the Author):

I thank the authors for their efforts in addressing my previous comments. I have some follow-up questions and suggestions regarding comment (3) on the “EA–IQ genetic associations” and the RVB burden tests of IQ. I appreciate that the authors now have a clearer understanding of this question and that they have more carefully expanded the discussion, and I find that the revised interpretations have been improved.

(i) The authors note that “We apologise for our carelessness in claiming previously that the EA-PGI was constructed to model an individual’s additive genetic liability IQ, which is of course incorrect. We do note, however, that in our main analyses we used not only a PGI for EA, but also a PGI for the cognitive component of EA (from Demange et al., Nature Genetics, 2021), which is effectively a PGI for adult cognitive performance (with some caveats/nuances, of course)...”

Could the authors use the most highly powered, publicly available GWAS of **real** cognitive performance/IQ phenotype to construct a **real** PGI for cognitive performance (CP-PGI), redo the association analyses, and examine whether they obtain similar conclusions to those based on the EA-PGI and the PGI for the cognitive component of EA, particularly with respect to their main conclusion comparing the “polygenic indices association" and the "RVB association”?

If the EA-PGI and CP-PGI yield similar conclusions, this would substantially strengthen the authors’ arguments. In that case, it would also be helpful to explain why a CP-PGI was not used from the outset, and whether this motivates any proper revision of the analysis plan or framing.

If, however, the CP-PGI and EA-PGI lead to different conclusions, this would suggest that the current reasoning may not fully hold and would require more careful investigation.

We thank the reviewer for this suggestion. As requested, we have now constructed a PGI using the Savage et al. GWAS of measured intelligence (Nature Genetics, 2018) (PGI_{CP}), which represents the most highly powered, published, publicly available GWAS of directly measured cognitive performance/IQ at the time of our analysis. We did not use the Savage et al. PGI from the outset because PGI_{Cog} allowed us to simultaneously examine the cognitive and non-cognitive components of educational attainment within a unified analytical framework (following Demange et al.), which was a key aim of our study. Additionally, PGI_{Cog} was marginally more predictive of IQ than PGI_{CP} in the population models, showing slightly higher effect sizes (see Response Table 1 below). Nevertheless, we repeated all PGI analyses on IQ in ALSPAC using this intelligence PGI and present the results in new Supplementary Table 13.

The results are extremely similar to those obtained using PGI_{Cog} across key results (Response Table 1), with no conclusions changed. Specifically, we observe: (1) significant positive population and direct main effects on IQ; (2) significant positive population and direct age interaction effects (age interaction beta = 0.002 and 0.004, p =$7x10^{-4}$ and 0.0014, respectively), indicating that associations increase in magnitude as children age; and (3) concordant patterns across quantile regressions, demonstrating the age interaction effects

are stronger at the top than the bottom of the IQ distribution (5th and 95th percentile age interaction beta = -0.01 and 0.07 per ten years, p = 0.71 and 0.009, respectively). These findings confirm that our main conclusions hold when using a PGI derived from another GWAS of cognitive performance.

**Model**	**Effect**	**Sample**	**Outcome**	**PGI_{Cog} (cognitive component of EA)**		**PGI_{CP} (Savage et al. intelligence GWAS)**	
				**Beta**	**95% CI**	**Beta**	**95% CI**
Cross-sectional	Population	Full	IQ Age 4	0.269	[0.243, 0.296]	0.263	[0.239, 0.288]
Cross-sectional	Population	Full	IQ Age 8	0.283	[0.257, 0.310]	0.279	[0.255, 0.304]
Cross-sectional	Population	Full	IQ Age 16	0.307	[0.280, 0.334]	0.292	[0.268, 0.316]
Cross-sectional	Direct	Trio	IQ Age 4	0.131	[0.080, 0.182]	0.133	[0.082, 0.185]
Cross-sectional	Direct	Trio	IQ Age 8	0.146	[0.095, 0.197]	0.144	[0.093, 0.195]
Cross-sectional	Direct	Trio	IQ Age 16	0.188	[0.136, 0.240]	0.169	[0.118, 0.220]
Mixed-effects	Population	Full	Main effec	0.28	[0.256, 0.304]	0.266	[0.242, 0.289]
Mixed-effects	Population	Full	Age interaction	0.002	[0.001, 0.004]	0.002	[0.001, 0.004]
Mixed-effects	Direct	Trio	Main effec	0.129	[0.079, 0.179]	0.129	[0.080, 0.179]
Mixed-effects	Direct	Trio	Age interaction	0.005	[0.002, 0.008]	0.004	[0.001, 0.007]
Quantile (longitudinal)	Population	Full	0.05 main	0.282	[0.213, 0.351]	0.245	[0.181, 0.310]
Quantile (longitudinal)	Population	Full	0.05 age interaction	0.001	[-0.003, 0.005]	-0.001	[-0.009, 0.006]
Quantile (longitudinal)	Population	Full	0.5 main	0.282	[0.213, 0.351]	0.271	[0.245, 0.297]

Quantile (longitudinal)	Population	Full	0.5 age interaction	0.004	[0.002, 0.006]	0.004	[0.000, 0.007]
Quantile (longitudinal)	Population	Full	0.95 main	0.271	[0.205, 0.338]	0.23	[0.193, 0.267]
Quantile (longitudinal)	Population	Full	0.95 age interaction	0.008	[0.004, 0.012]	0.007	[0.002, 0.013]
Quantile (longitudinal)	Direct	Trio	0.05 main	0.104	[0.042, 0.166]	0.0156	[-0.068, 0.180]
Quantile (longitudinal)	Direct	Trio	0.05 age interaction	0.001	[-0.008, 0.010]	-0.006	[-0.021, 0.009]
Quantile (longitudinal)	Direct	Trio	0.5 main	0.137	[0.103, 0.170]	0.138	[0.084, 0.193]
Quantile (longitudinal)	Direct	Trio	0.5 age interaction	0.005	[0.001, 0.009]	0.002	[-0.006, 0.010]
Quantile (longitudinal)	Direct	Trio	0.95 main	0.146	[0.089, 0.203]	0.157	[0.083, 0.231]
Quantile (longitudinal)	Direct	Trio	0.95 age interaction	0.013	[0.006, 0.021]	0.007	[0.001, 0.013]

Response Table 1. Comparison of key results presented in the paper between PGI_{Cog} and a PGI_{CP} constructed from the Savage et al. intelligence GWAS.

This concordance is expected given that the GWAS summary statistics that were used to construct PGI_{Cog} have a genetic correlation not significantly different from 1 with the Savage et al. intelligence GWAS (LDSC r_g = 1.02, 95% CI [0.96–1.09]), which we now note in the manuscript at the point where we introduce the PGIs (line 115) “Notably, the cognitive component has a genetic correlation not significantly different from 1 with a GWAS meta-analysis of intelligence[9] (LDSC r_g = 1.02, 95% confidence interval [0.96-1.09])”. Nonetheless, we appreciate the reviewer prompting us to demonstrate empirically that our conclusions are robust to using a PGI derived from a direct cognitive phenotype GWAS.

We have added a sentence to the Supplementary Discussion noting this robustness analysis (line 917): "As a robustness analysis, we repeated all analyses of IQ in ALSPAC with a PGS constructed from another GWAS of cognitive performance (Savage et al. 2018), which yielded results with identical statistical interpretations to those for PGI_{Cog} (Supplementary Table 13)."

(ii) Unless the authors decide to use a real cognitive performance GWAS to construct CP-PGI as their primary analysis, it is important to specify the source of the “polygenic indices consistently”. In particular, whenever “polygenic indices” are mentioned, please clarify that they are derived from EA (or from the cognitive component of EA). Otherwise, readers may assume they come from a cognitive performance GWAS.

For example, in the abstract, where the text currently refers to “between polygenic indices and” and “associations with polygenic indices,” it should be made explicit that these are EA-based polygenic indices; otherwise, this wording is somewhat misleading.

We agree with the reviewer that clarity regarding the source of polygenic indices is important throughout the manuscript. We have now systematically revised the manuscript to explicitly specify the source of the PGIs wherever they are mentioned. Specifically, we have updated the following sections to clarify that the PGIs analyzed are derived from GWASs of educational attainment and its cognitive component (or, where relevant, its non-cognitive component):

- We changed the abstract to explicitly state the source of the PGIs mentioned in the main text: “we show that, as children age, the association between cognitive ability and polygenic indices **for educational attainment and its cognitive component** increases”
- The Introduction/aims paragraph now specifies "In this work, we sought to dissect the contribution of polygenic indices for educational attainment and its cognitive and noncognitive components"
- Throughout the Discussion, we have replaced generic references to "PGIs" with explicit descriptions (e.g., "PGIs for educational attainment and its cognitive component", “PGIs considered”)
- Figure 1 legend now clarifies the PGIs examined
- The Supplementary Discussion sections have been updated throughout

We believe these revisions ensure that readers will not mistakenly assume the PGIs were derived directly from a cognitive performance GWAS, while the change mentioned above also makes it clear that PGI_{Cog} has a genetic correlation indistinguishable from 1 with measured intelligence (line 115).
